# Supplementary material for: A unifying mechanism for the biogenesis of membrane proteins co-operatively integrated by the Sec and Tat pathways
Source: eLife. 2017 May 17;6:e26577. doi: 10.7554/eLife.26577 (PMC5449189; doi:10.7554/eLife.26577)
Supplement: Table 1—source data 1. — DOI: http://dx.doi.org/10.7554/eLife.26577.008 [file elife-26577-table1-data1.docx]

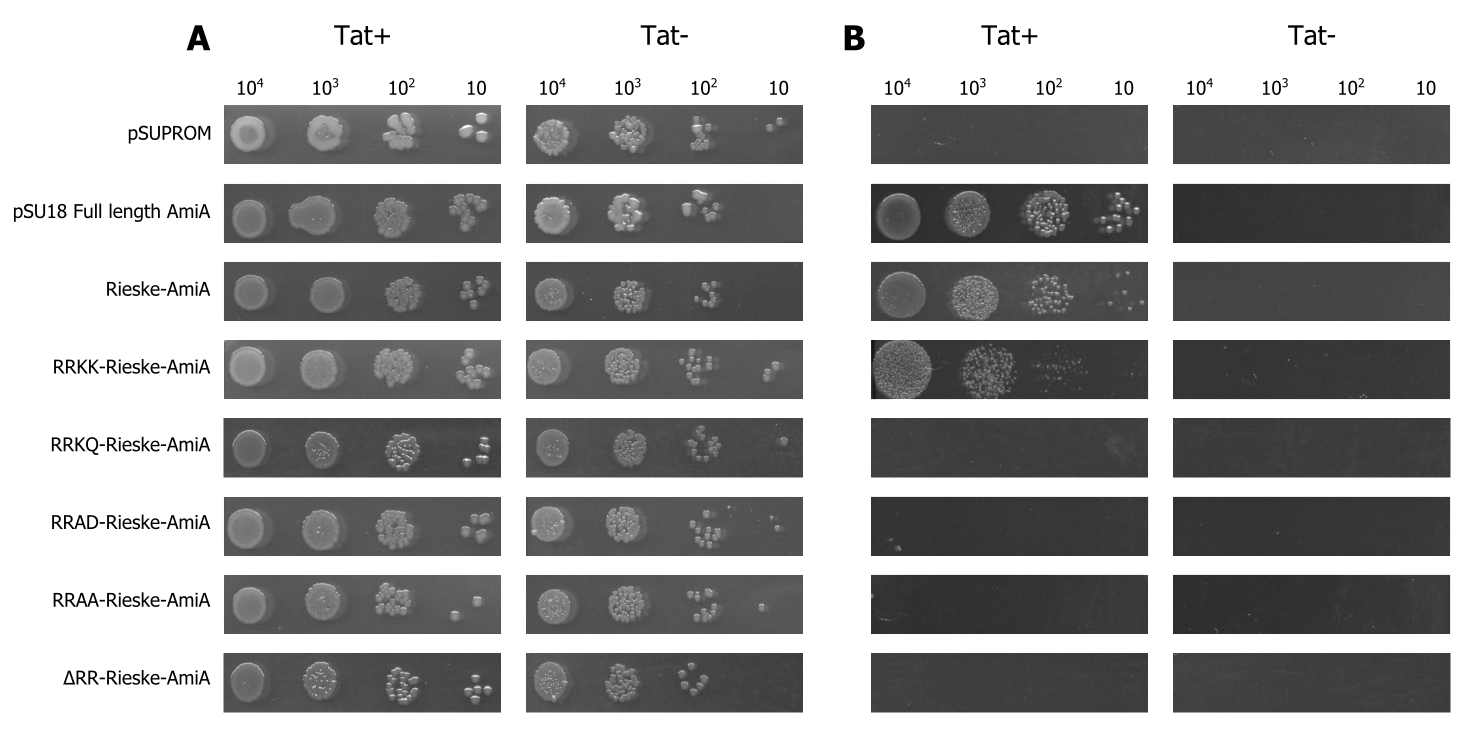


Representative spot tests of strains MCDSSAC (*tat^+^* strain) and MCDSSACΔtat (*tat^-^* strain) harbouring the pSUPROM vector encoding native (full length) AmiA, Rieske-AmiA or the indicated substitutions at the twin arginine motif of Rieske-AmiA. Strains were grown overnight in liquid media, diluted to give serial dilutions of 10, 10^2^, 10^3^ and 10^4^ cells per 5 µl aliquots which were spotted onto LB solid agar (A) and LB solid agar containing 1% (w/v) SDS (B). LB agar plates were incubated at 37°C for 16 h.


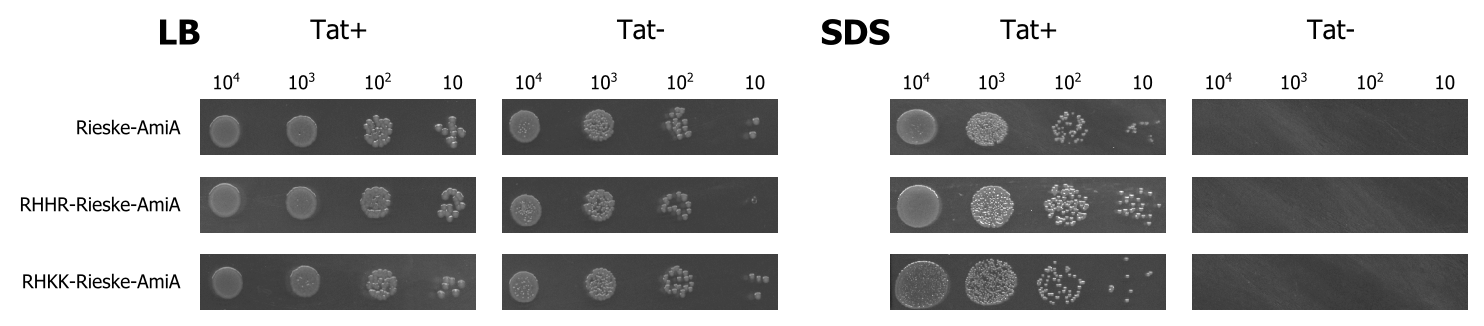


Representative spot tests of strains MCDSSAC (*tat^+^* strain) and MCDSSACΔtat (*tat^-^* strain) harbouring the pSUPROM vector encoding Rieske-AmiA or RH motif variants of Rieske-AmiA. Strains were grown overnight in liquid media, diluted to give serial dilutions of 10, 10^2^, 10^3^ and 10^4^ cells per 5 µl aliquots which were spotted onto LB solid agar (A) and LB solid agar containing 1% (w/v) SDS (B). LB agar plates were incubated at 37°C for 16 h.


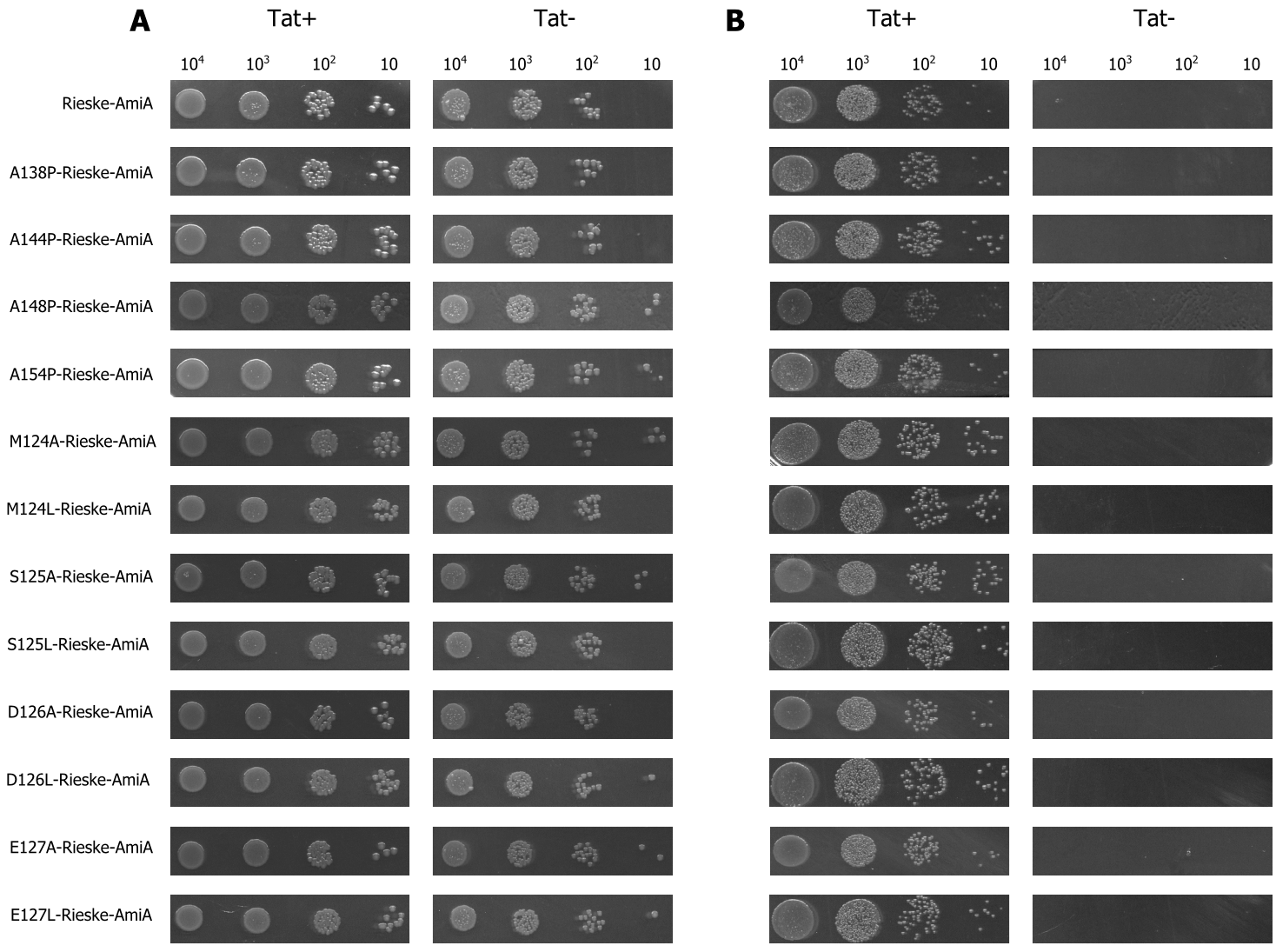


Representative spot tests of strains MCDSSAC (*tat^+^* strain) and MCDSSACΔtat (*tat^-^* strain) harbouring the pSUPROM vector encoding Rieske-AmiA or predicted α–helix fusion variants (A138P-Rieske-AmiA, A144P-Rieske-AmiA, A148P-Rieske-AmiA and A154P-Rieske-AmiA) or negative charge region fusion variants (M124A-Rieske-AmiA, M124L-Rieske-AmiA, S125A-Rieske-AmiA, S125A-Rieske-AmiA, D126A-Rieske-AmiA, D126A-Rieske-AmiA E127A-Rieske-AmiA and E127L-Rieske-AmiA). Strains were grown overnight in liquid media, diluted to give serial dilutions of 10, 10^2^, 10^3^ and 10^4^ cells per 5 µl aliquots which were spotted onto LB solid agar (A) and LB solid agar containing 1% (w/v) SDS (B). LB agar plates were incubated at 37°C for 16 h.


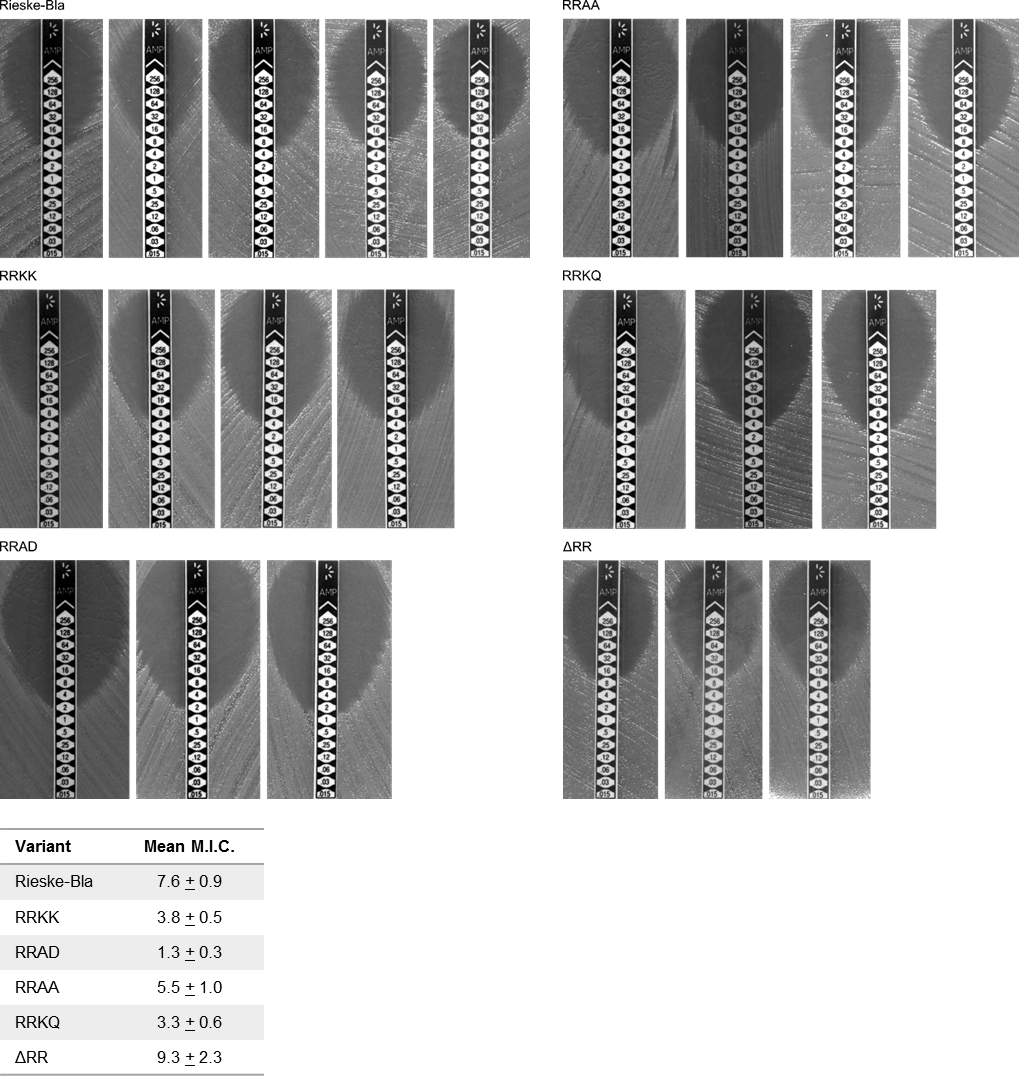


M.I.C.Evaluator^TM^ strip test of DADE (*tat*^-^) harbouring the pSUPROM vector encoding Rieske-Bla and twin‑arginine variants (RRKK-Rieske-Bla, RRAD-Rieske-Bla, RRAA-Rieske-Bla, RRKQ-Rieske-Bla and ΔRR-Rieske-Bla). Stationary phase cultures were diluted to OD_600_ 0.1 and a lawn of bacteria was spread onto LB agar plates, M.I.C.Evaluator^TM^ strips were placed on the lawn and the plate was incubated at 37°C for 18 h. The M.I.C. (μg/ml) for ampicillin is read at the intersection of the test strip and the clearing of bacteria. The table indicates the mean M.I.C. and ± s.d.


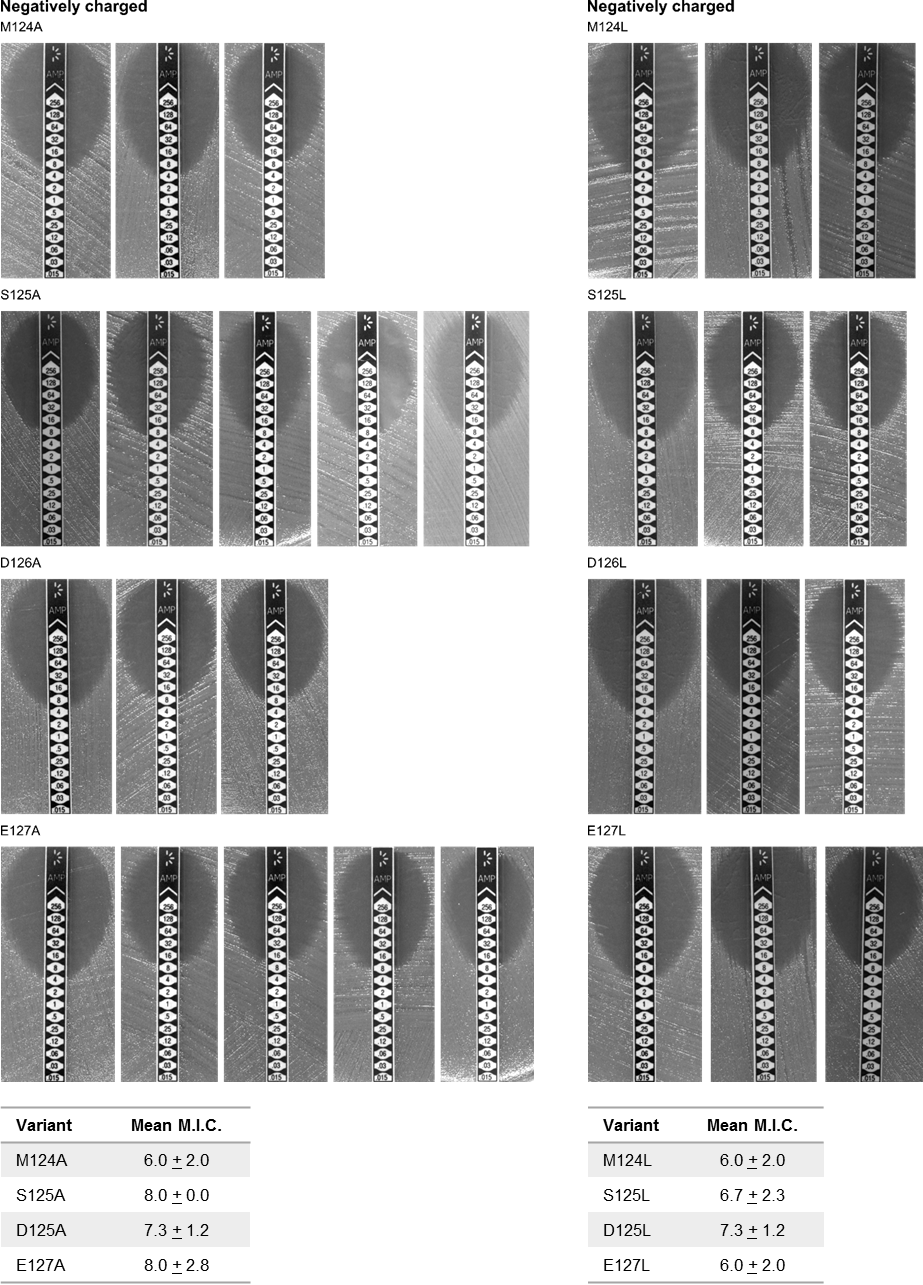


M.I.C.Evaluator^TM^ strip test of DADE (*tat*^-^) harbouring the pSUPROM vector encoding negatively charged region variants (M124A-Rieske-Bla, S125A-Rieske-Bla, D126A-Rieske-Bla, E127A-Rieske-Bla M124L-Rieske-Bla, S125L-Rieske-Bla, D126L-Rieske-Bla and E127L-Rieske-Bla). Stationary phase cultures were diluted to OD_600_ 0.1 and a lawn of bacteria was spread onto LB agar plates, M.I.C.EvaluatorTM strips were placed on the lawn and the plate was incubated at 37°C for 18 h. The M.I.C. (μg/ml) for ampicillin is read at the intersection of the test strip and the clearing of bacteria. The tables indicate the mean M.I.C. and ± s.d.


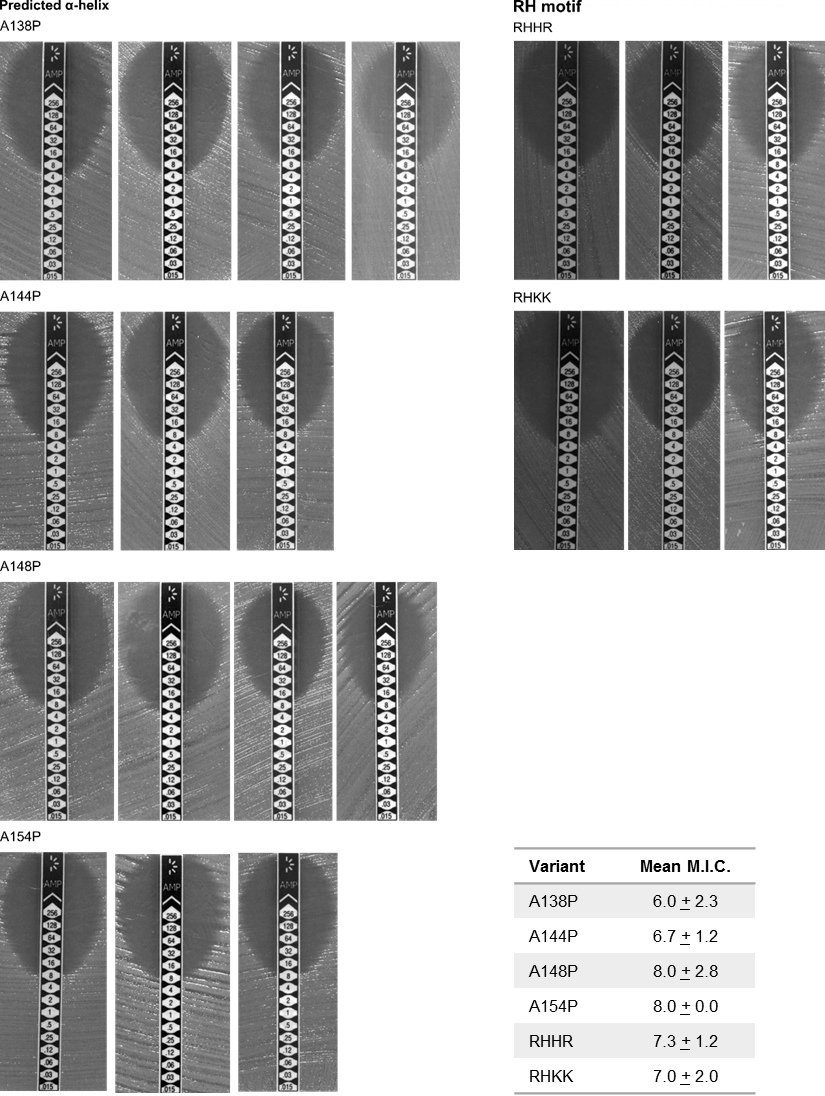


M.I.C.Evaluator^TM^ strip test of DADE (*tat*^-^) harbouring the pSUPROM vector encoding predicted α-helix (A138P-Rieske-Bla, A144P-Rieske-Bla, A148P-Rieske-Bla, A154P-Rieske-Bla) or RH variants (RHHR-Rieske-Bla, and RHKK-Rieske-Bla). Stationary phase cultures were diluted to OD_600_ 0.1 and a lawn of bacteria was spread onto LB agar plates, M.I.C.Evaluator^TM^ strips were placed on the lawn and the plate was incubated at 37°C for 18 h. The M.I.C. (μg/ml) for ampicillin is read at the intersection of the test strip and the clearing of bacteria. The tables indicate the mean M.I.C. and ± s.d.


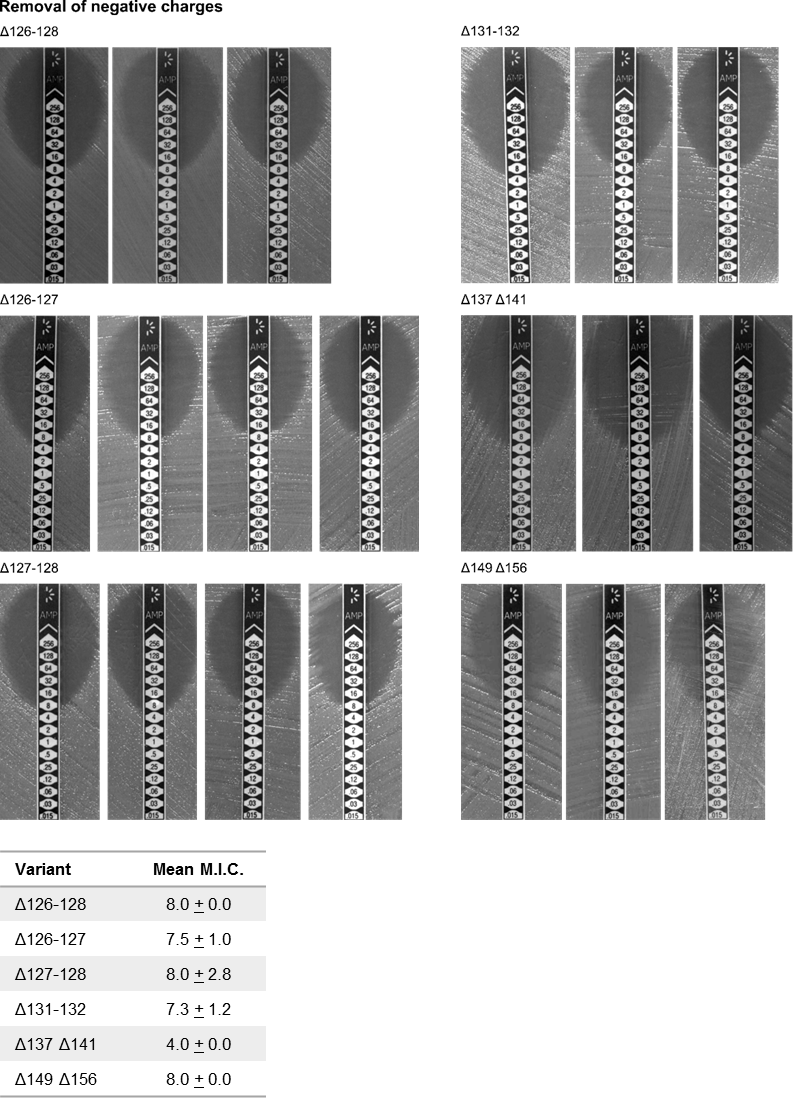


M.I.C.Evaluator^TM^ strip test of DADE (*tat*^-^) harbouring the pSUPROM vector encoding deletions of negatively charged variants (Δ126-128-Rieske-Bla, Δ126-127-Rieske-Bla, Δ127-128-Rieske-Bla, Δ131-132-Rieske-Bla, Δ137Δ141-Rieske-Bla and Δ149Δ156-Rieske-Bla). Stationary phase cultures were diluted to OD_600_ 0.1 and a lawn of bacteria was spread onto LB agar plates, M.I.C.Evaluator^TM^ strips were placed on the lawn and the plate was incubated at 37°C for 18 h. The M.I.C. (μg/ml) for ampicillin is read at the intersection of the test strip and the clearing of bacteria. The tables indicate the mean M.I.C. and ± s.d.


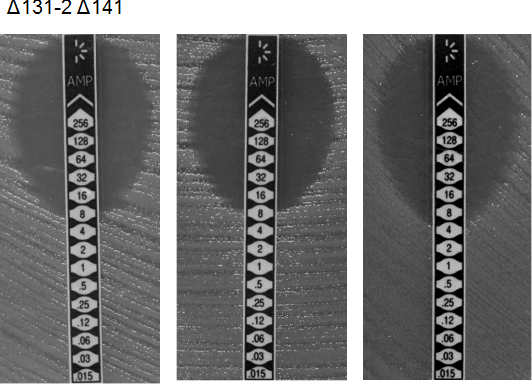


| Variant | Mean M.I.C. |
| --- | --- |
| Δ131-2 Δ141 | 6.0 (+2.0, n=3) |

M.I.C.Evaluator^TM^ strip test of DADE (*tat*^-^) harbouring the pSUPROM vector encoding deletions of negatively charged variant Δ131-132Δ141-Rieske-Bla. Stationary phase cultures were diluted to OD_600_ 0.1 and a lawn of bacteria was spread onto LB agar plates, M.I.C.Evaluator^TM^ strips were placed on the lawn and the plate was incubated at 37°C for 18 h. The M.I.C. (μg/ml) for ampicillin is read at the intersection of the test strip and the clearing of bacteria. The table indicates the mean M.I.C. and ± s.d.


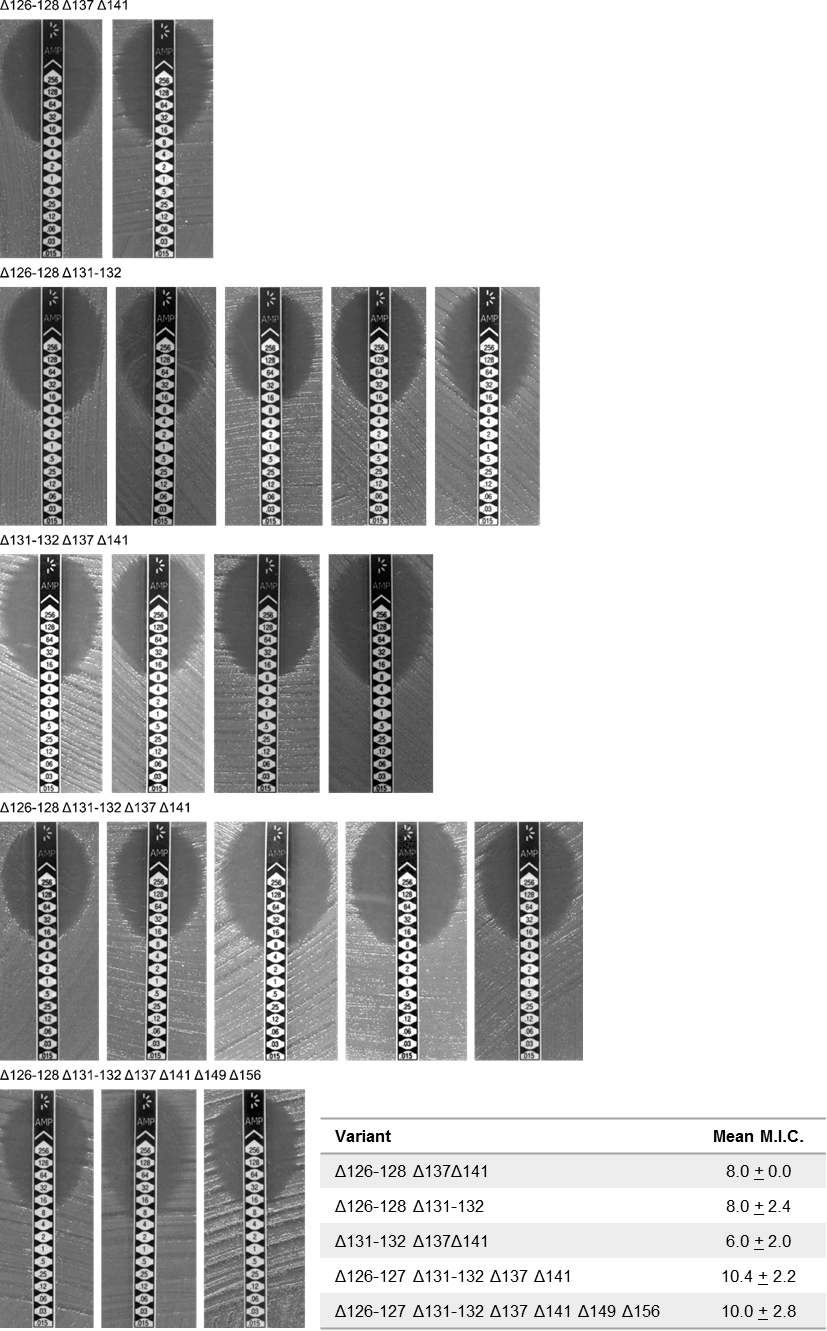


M.I.C.Evaluator^TM^ strip test of DADE (*tat*^-^) harbouring the pSUPROM vector encoding deletions of negatively charged variants (Δ128-128Δ137Δ141-Rieske-Bla, Δ126-128Δ131-132-Rieske-Bla, Δ131-132Δ137Δ141-Rieske-Bla, Δ126-128Δ131-132Δ137Δ141-Rieske-Bla and Δ126-128Δ131-132Δ137Δ141Δ149Δ156-Rieske-Bla). Stationary phase cultures were diluted to OD_600_ 0.1 and a lawn of bacteria was spread onto LB agar plates, M.I.C.Evaluator^TM^ strips were placed on the lawn and the plate was incubated at 37°C for 18 h. The M.I.C. (μg/ml) for ampicillin is read at the intersection of the test strip and the clearing of bacteria. The table indicates the mean M.I.C. and ± s.d.


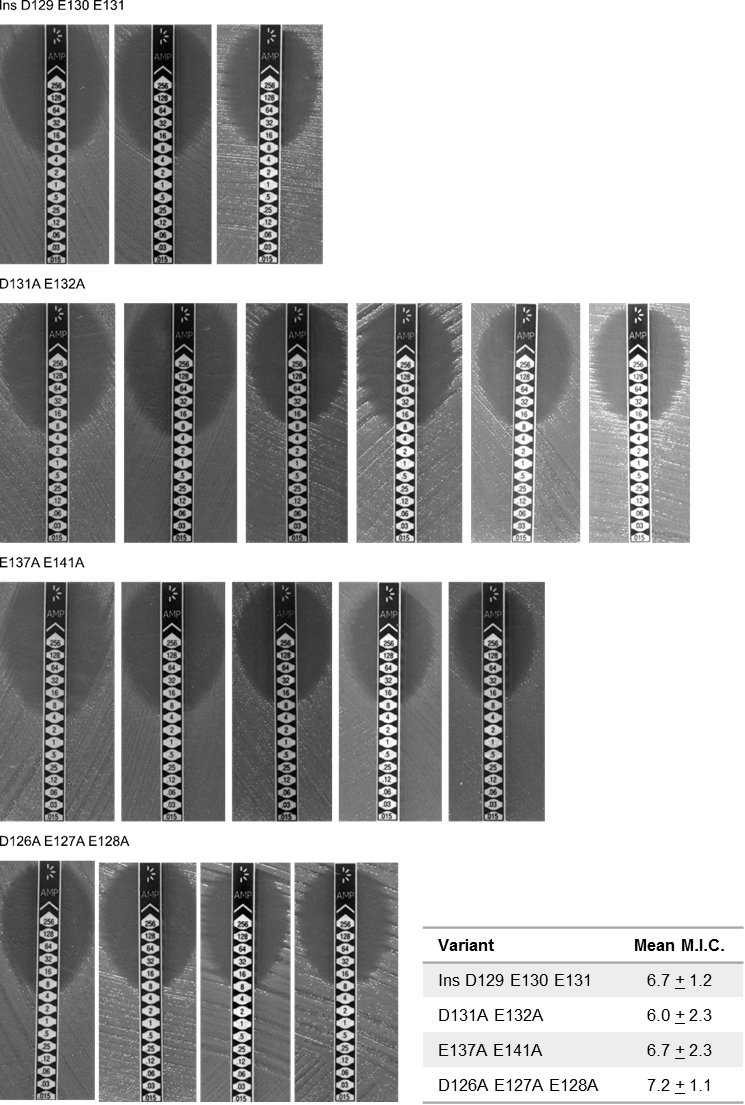


M.I.C.Evaluator^TM^ strip test of DADE (*tat*^-^) harbouring the pSUPROM vector encoding negatively charged variants (InsD129E130E131-Rieske-Bla, D131AE132A-Rieske-Bla, E137AE141A-Rieske-Bla, and D126AE127AE128A-Rieske-Bla). Stationary phase cultures were diluted to OD_600_ 0.1 and a lawn of bacteria was spread onto LB agar plates, M.I.C.Evaluator^TM^ strips were placed on the lawn and the plate was incubated at 37°C for 18 h. The M.I.C. (μg/ml) for ampicillin is read at the intersection of the test strip and the clearing of bacteria. The tables indicate the mean M.I.C. and ± s.d.


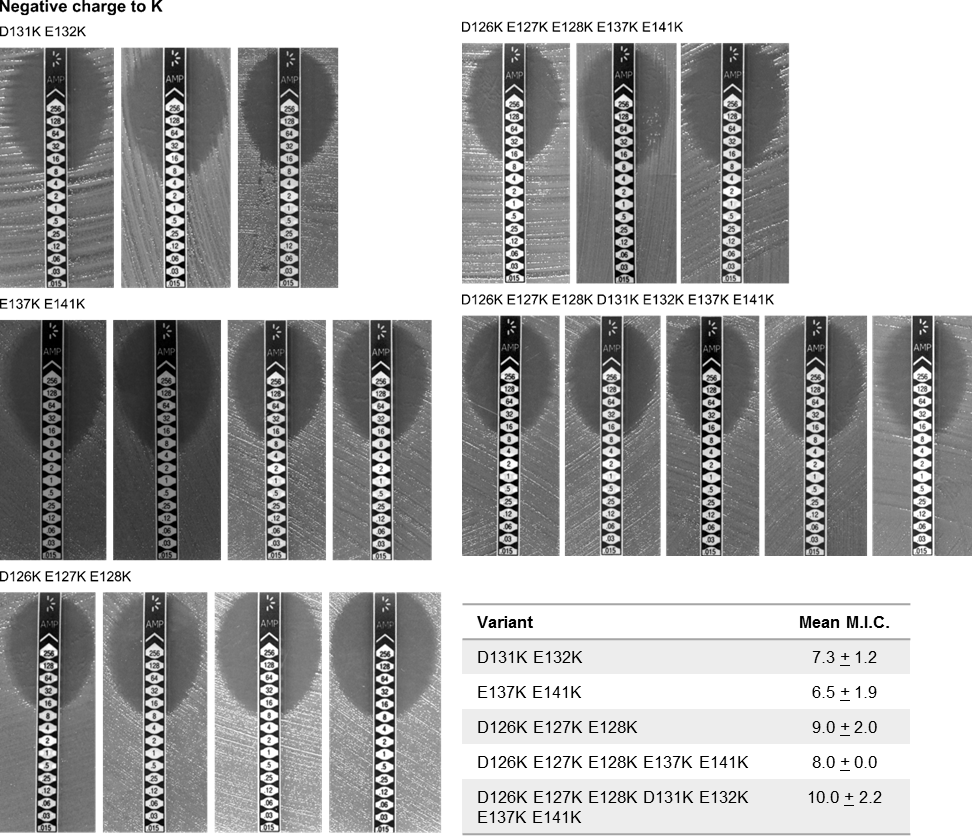


M.I.C.Evaluator^TM^ strip test of DADE (*tat*^-^) harbouring the pSUPROM vector encoding negatively charged variants (D131KE132K-Rieske-Bla, E137KE141K-Rieske-Bla, D126KE127KE128K-Rieske-Bla, D126KE127KE128KE137KE141K-Rieske-Bla and D126KE127KE128KD131KE132KE137KE141K -Rieske-Bla). Stationary phase cultures were diluted to OD_600_ 0.1 and a lawn of bacteria was spread onto LB agar plates, M.I.C.Evaluator^TM^ strips were placed on the lawn and the plate was incubated at 37°C for 18 h. The M.I.C. (μg/ml) for ampicillin is read at the intersection of the test strip and the clearing of bacteria. The table indicates the mean M.I.C. and ± s.d.
