# Supplementary material for: A unifying mechanism for the biogenesis of membrane proteins co-operatively integrated by the Sec and Tat pathways
Source: eLife. 2017 May 17;6:e26577. doi: 10.7554/eLife.26577 (PMC5449189; doi:10.7554/eLife.26577)
Supplement: Table 2—source data 1. — DOI: http://dx.doi.org/10.7554/eLife.26577.010 [file elife-26577-table2-data1.docx]

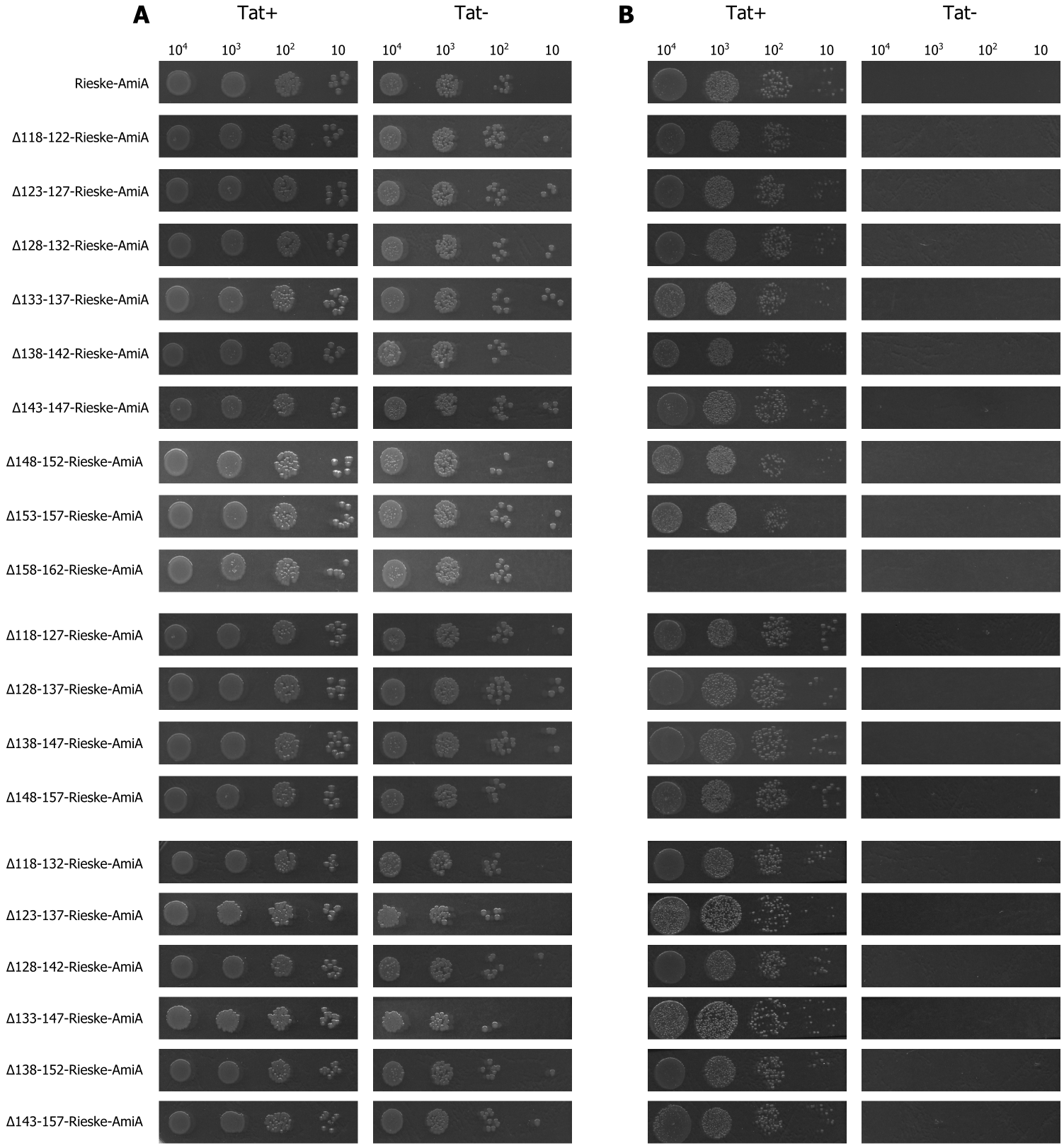


Representative spot tests of strains MCDSSAC (*tat^+^* strain) and MCDSSACΔtat (*tat^-^* strain) harbouring the pSUPROM vector encoding Rieske-AmiA or 5 aa deletion fusion variants (Δ118-122-Rieske-AmiA, Δ123-127-Rieske-AmiA, Δ128-132-Rieske-AmiA, Δ133-137-Rieske-AmiA, Δ138-142-Rieske-AmiA, Δ143-147-Rieske-AmiA, Δ148-152-Rieske-AmiA Δ153-157-Rieske-AmiA and Δ158-162-Rieske-AmiA) or 10 aa deletion fusion variants (Δ118-127-Rieske-AmiA, Δ128-137-Rieske-AmiA, Δ138-147-Rieske-AmiA, and Δ148-157-Rieske-AmiA) or 15 aa deletion fusion variants (Δ118-132-Rieske-AmiA, Δ123-137-Rieske-AmiA, Δ128-142-Rieske-AmiA, Δ133-147-Rieske-AmiA, Δ138-152-Rieske-AmiA, and Δ143-157-Rieske-AmiA). Strains were grown overnight in liquid media, diluted to give serial dilutions of 10, 10^2^, 10^3^ and 10^4^ cells per 5 µl aliquots which were spotted onto LB solid agar (A) and LB solid agar containing 1% (w/v) SDS (B). LB agar plates were incubated at 37°C for 16 h.


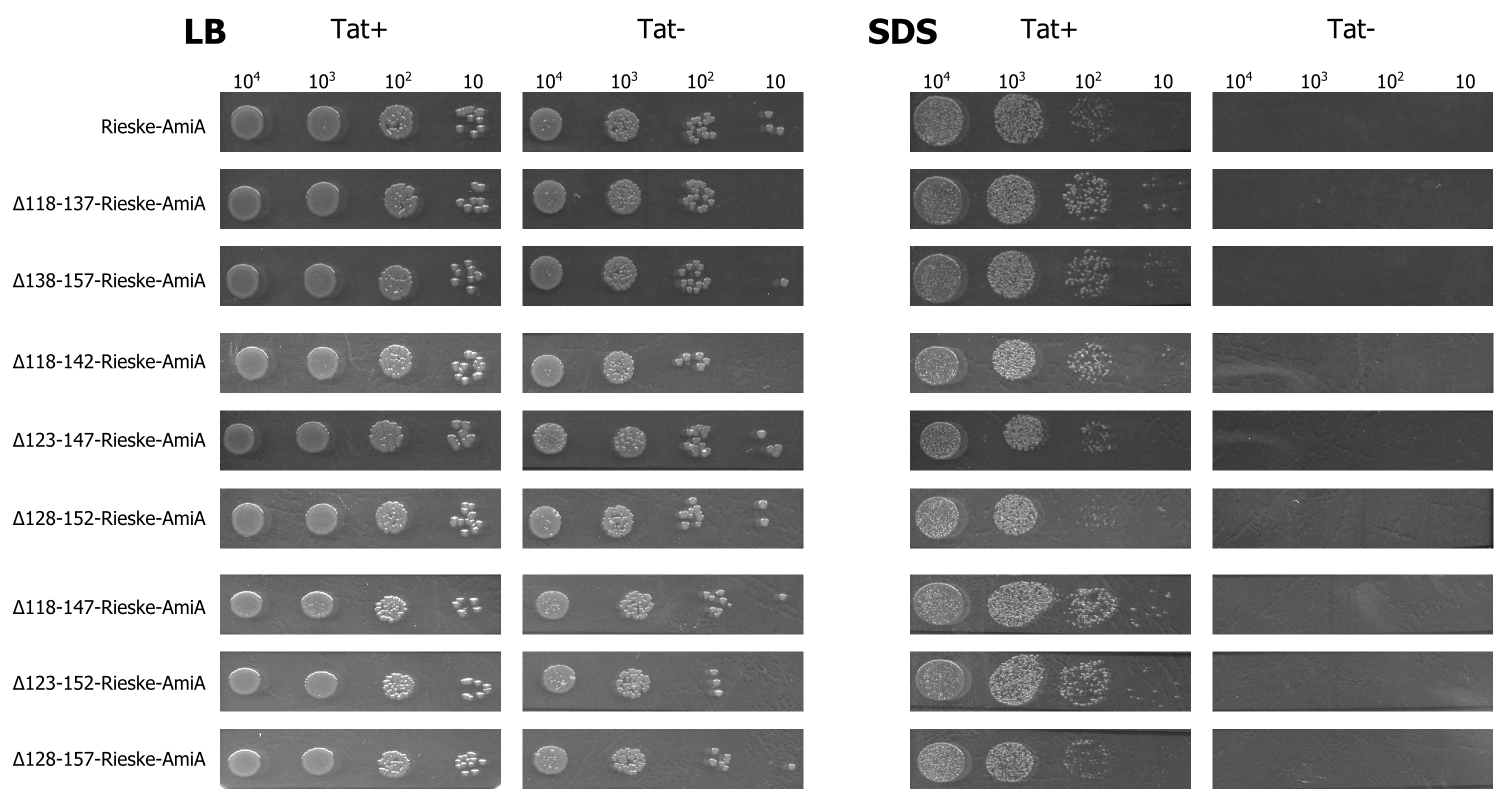


Representative spot tests of strains MCDSSAC (*tat^+^* strain) and MCDSSACΔtat (*tat^-^* strain) harbouring the pSUPROM vector encoding Rieske-AmiA or 20 aa deletion fusion variants (Δ118-137-Rieske-AmiA and Δ138-157-Rieske-AmiA) or 25 aa deletion fusion variants (Δ118-142-Rieske-AmiA, Δ123-147-Rieske-AmiA, and Δ128-152-Rieske-AmiA) or 30 aa deletion fusion variants (Δ118-147-Rieske-AmiA, Δ123-152-Rieske-AmiA, and Δ128-157-Rieske-AmiA). Strains were grown overnight in liquid media, diluted to give serial dilutions of 10, 10^2^, 10^3^ and 10^4^ cells per 5 µl aliquots which were spotted onto LB solid agar (A) and LB solid agar containing 1% (w/v) SDS (B). LB agar plates were incubated at 37°C for 16 h.


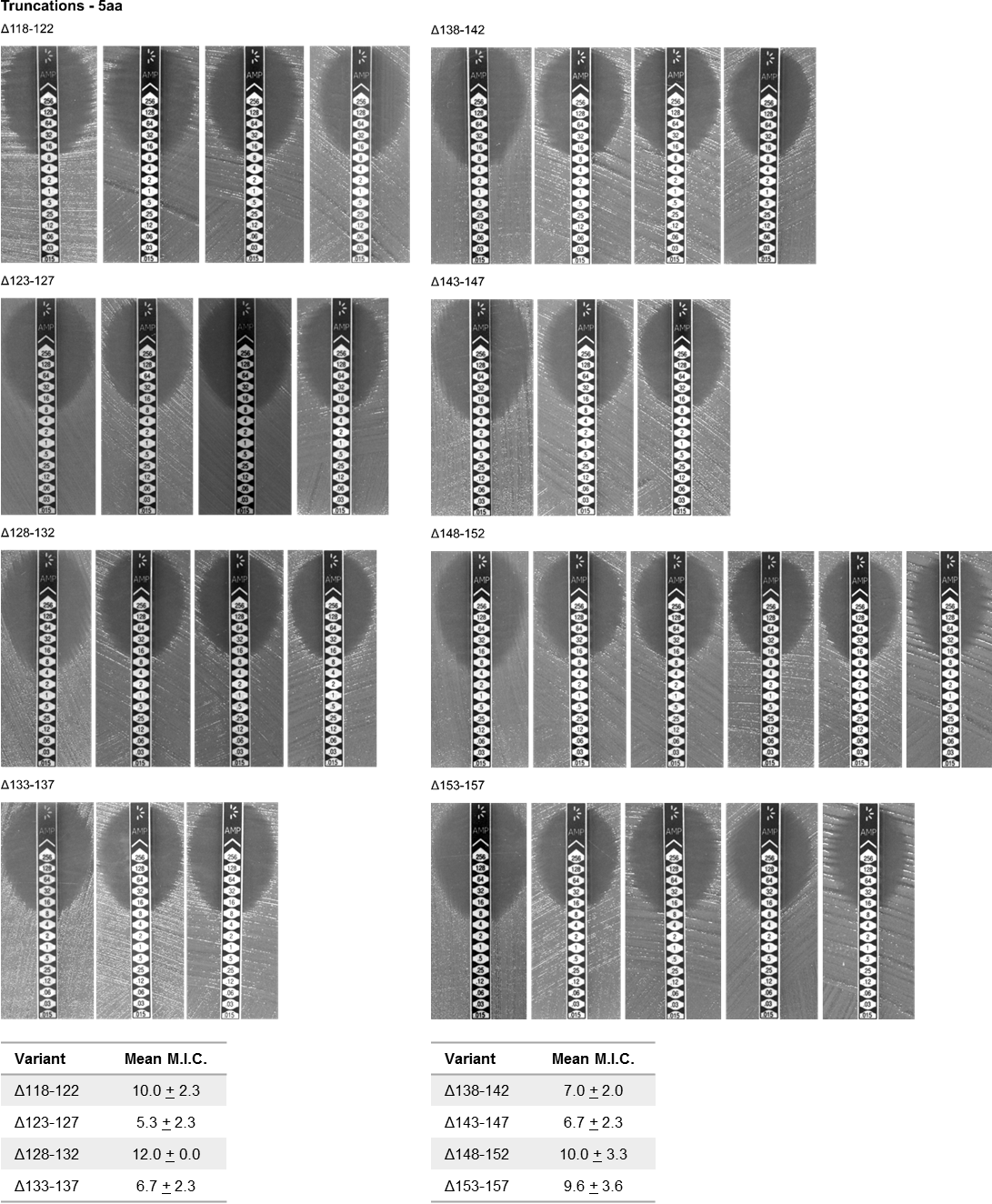


M.I.C.Evaluator^TM^ strip test of DADE (*tat*^-^) harbouring the pSUPROM vector encoding truncations of 5aa variants (Δ118-122-Rieske-Bla, Δ123-127-Rieske-Bla, Δ128-132-Rieske-Bla, Δ133-137-Rieske-Bla, Δ138-142-Rieske-Bla, Δ143-147-Rieske-Bla, Δ148-152-Rieske-Bla and Δ153-157-Rieske-Bla). Stationary phase cultures were diluted to OD_600_ 0.1 and a lawn of bacteria was spread onto LB agar plates, M.I.C.Evaluator^TM^ strips were placed on the lawn and the plate was incubated at 37°C for 18 h. The M.I.C. (μg/ml) for ampicillin is read at the intersection of the test strip and the clearing of bacteria. The tables indicate the mean M.I.C. and ± s.d.


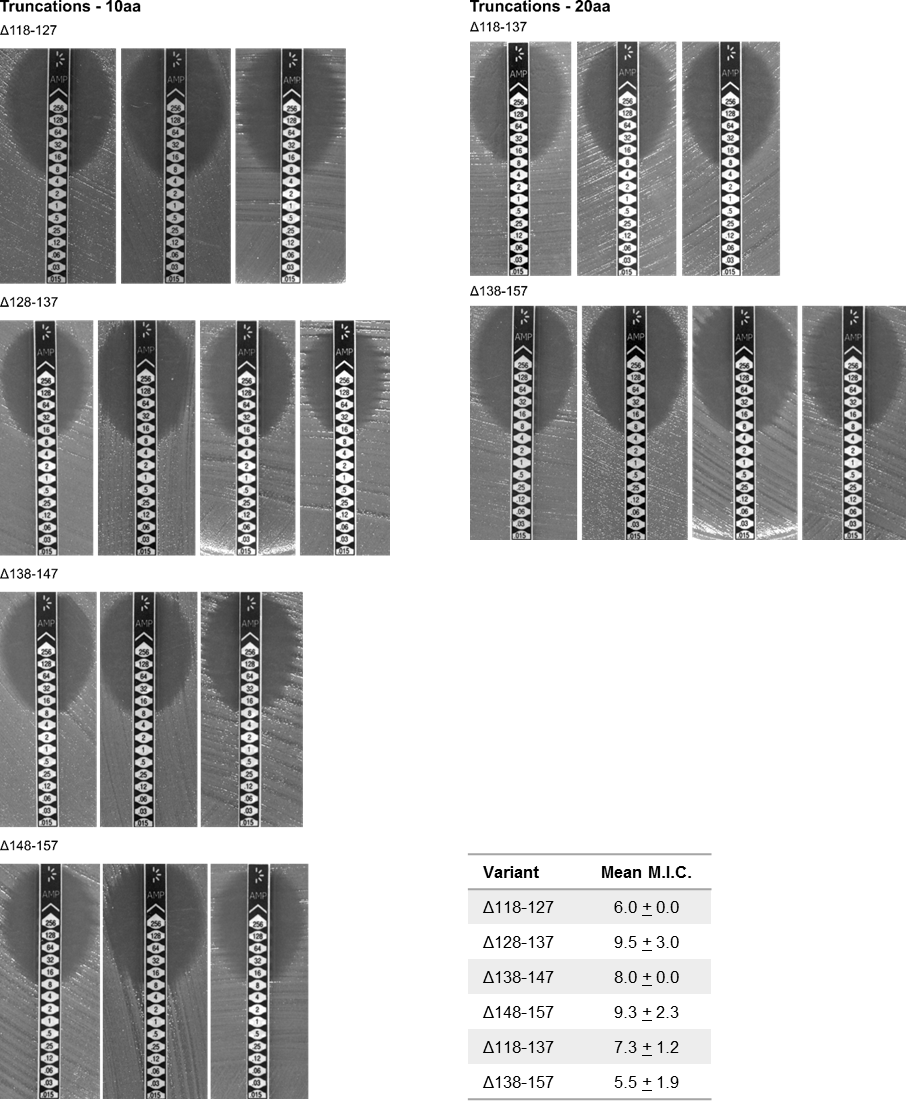


M.I.C.Evaluator^TM^ strip test of DADE (*tat*^-^) harbouring the pSUPROM vector encoding truncations of 10aa (Δ118-127-Rieske-Bla, Δ123-137-Rieske-Bla, Δ138-147-Rieske-Bla, Δ148-157-Rieske-Bla) or 20aa variants (Δ118-137-Rieske-Bla and Δ138-157-Rieske-Bla). Stationary phase cultures were diluted to OD_600_ 0.1 and a lawn of bacteria was spread onto LB agar plates, M.I.C.Evaluator^TM^ strips were placed on the lawn and the plate was incubated at 37°C for 18 h. The M.I.C. (μg/ml) for ampicillin is read at the intersection of the test strip and the clearing of bacteria. The tables indicate the mean M.I.C. and ± s.d.


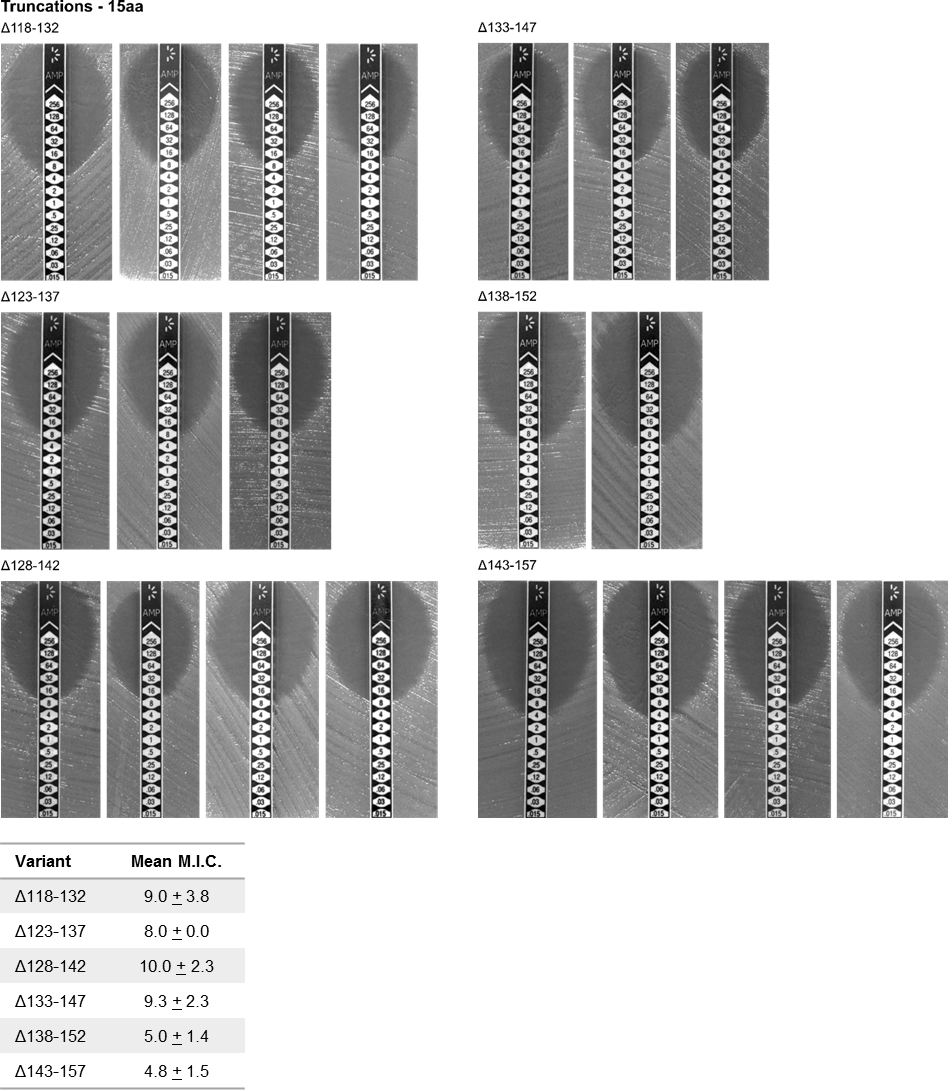


M.I.C.Evaluator^TM^ strip test of DADE (*tat*^-^) harbouring the pSUPROM vector encoding truncations of 15aa variants (Δ118-132-Rieske-Bla, Δ123-137-Rieske-Bla, Δ128-142-Rieske-Bla, Δ133-147-Rieske-Bla, Δ138-152-Rieske-Bla, and Δ143-157-Rieske-Bla). Stationary phase cultures were diluted to OD_600_ 0.1 and a lawn of bacteria was spread onto LB agar plates, M.I.C.Evaluator^TM^ strips were placed on the lawn and the plate was incubated at 37°C for 18 h. The M.I.C. (μg/ml) for ampicillin is read at the intersection of the test strip and the clearing of bacteria. The tables indicates the mean M.I.C. and ± s.d.


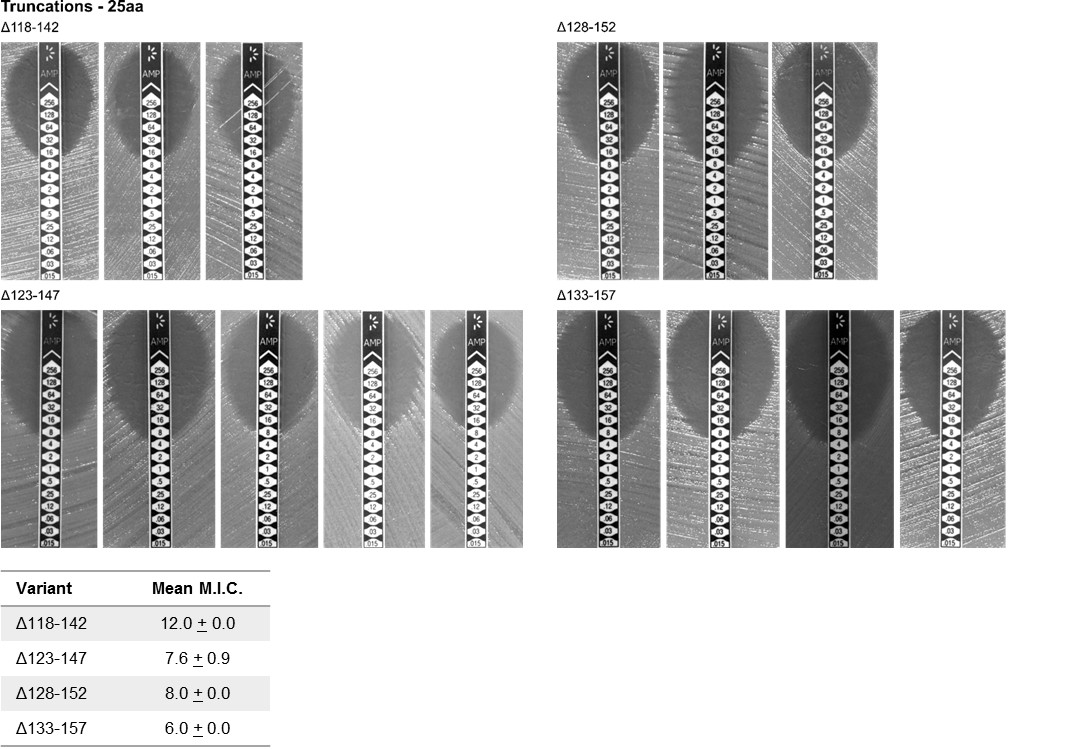


M.I.C.Evaluator^TM^ strip test of DADE (*tat*^-^) harbouring the pSUPROM vector encoding truncations of 25aa variants (Δ118-142-Rieske-Bla, Δ123-147-Rieske-Bla, Δ128-152-Rieske-Bla, and Δ133-157-Rieske-Bla). Stationary phase cultures were diluted to OD_600_ 0.1 and a lawn of bacteria was spread onto LB agar plates, M.I.C.Evaluator^TM^ strips were placed on the lawn and the plate was incubated at 37°C for 18 h. The M.I.C. (μg/ml) for ampicillin is read at the intersection of the test strip and the clearing of bacteria. The table indicates the mean M.I.C. and ± s.d.


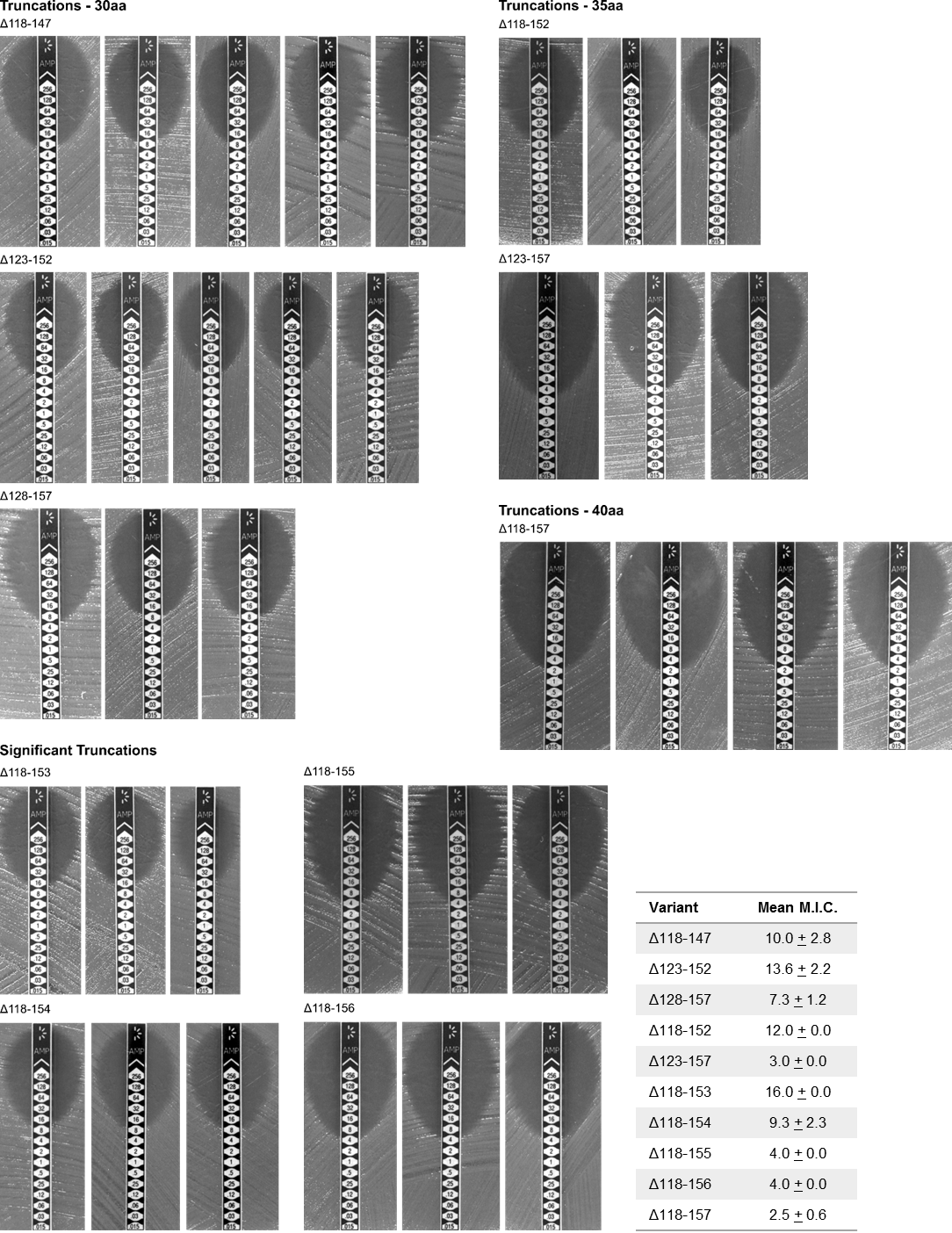


M.I.C.Evaluator^TM^ strip test of DADE (*tat*^-^) harbouring the pSUPROM vector encoding truncations of 30aa (Δ118-147-Rieske-Bla, Δ123-152-Rieske-Bla, Δ128-157-Rieske-Bla) or 35aa (Δ118-152-Rieske-Bla and Δ123-157-Rieske-Bla) or significant truncation variants (Δ118-153-Rieske-Bla, Δ118-154-Rieske-Bla, Δ118-155-Rieske-Bla, Δ118-156-Rieske-Bla and Δ118-157-Rieske-Bla). Stationary phase cultures were diluted to OD_600_ 0.1 and a lawn of bacteria was spread onto LB agar plates, M.I.C.Evaluator^TM^ strips were placed on the lawn and the plate was incubated at 37°C for 18 h. The M.I.C. (μg/ml) for ampicillin is read at the intersection of the test strip and the clearing of bacteria. The tables indicates the mean M.I.C. and ± s.d.


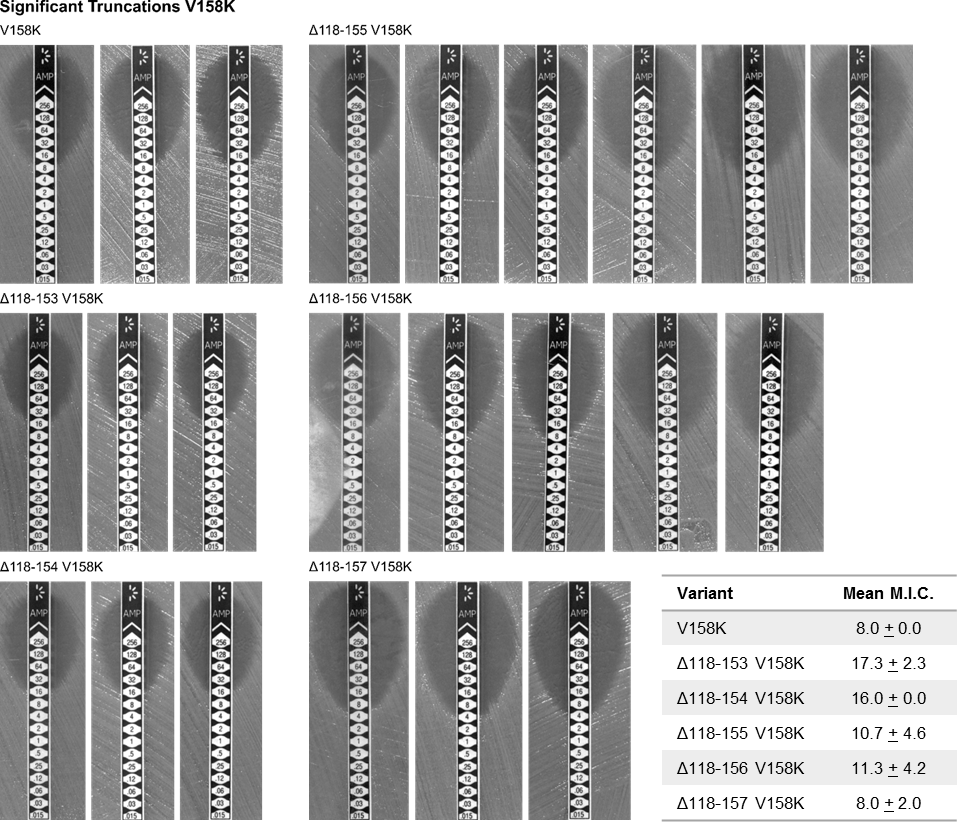


M.I.C.Evaluator^TM^ strip test of DADE (*tat*^-^) harbouring the pSUPROM vector encoding significant truncation variants (V158K, Δ118-153 V158K-Rieske-Bla, Δ118-154V158K-Rieske-Bla, Δ118-155V158K-Rieske-Bla, Δ118-156V158K-Rieske-Bla and Δ118-157V158K-Rieske-Bla). Stationary phase cultures were diluted to OD_600_ 0.1 and a lawn of bacteria was spread onto LB agar plates, M.I.C.Evaluator^TM^ strips were placed on the lawn and the plate was incubated at 37°C for 18 h. The M.I.C. (μg/ml) for ampicillin is read at the intersection of the test strip and the clearing of bacteria. The tables indicate the mean M.I.C. and ± s.d.
