# Supplementary figures and images for: A unifying mechanism for the biogenesis of membrane proteins co-operatively integrated by the Sec and Tat pathways
Source: eLife. 2017 May 17;6:e26577. doi: 10.7554/eLife.26577 (PMC5449189; doi:10.7554/eLife.26577)

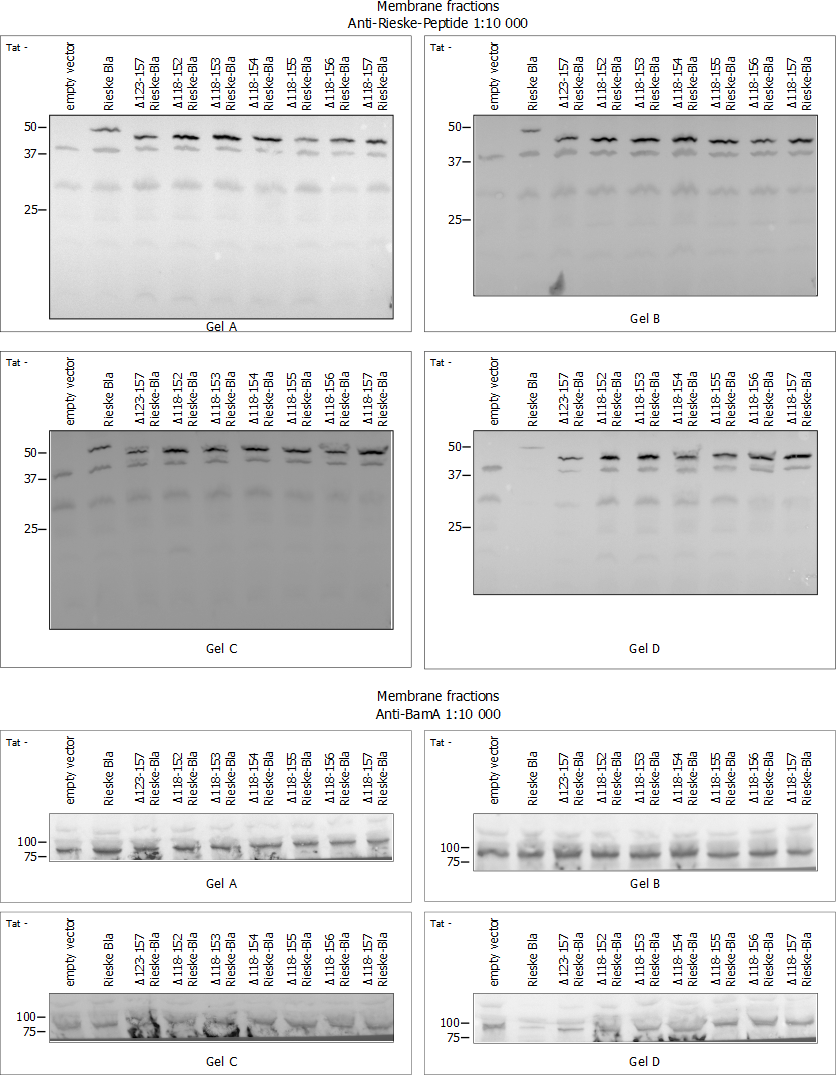

Supplement: Figure 2—source data 1. — DOI: http://dx.doi.org/10.7554/eLife.26577.012 [file elife-26577-fig2-data1.docx]

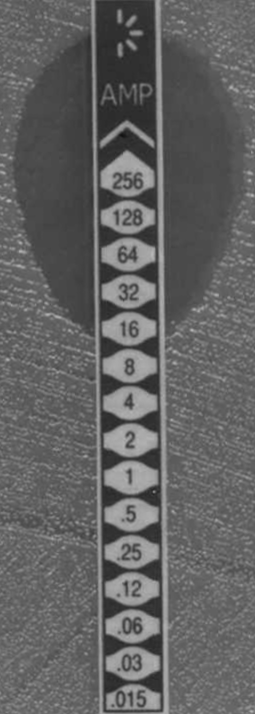

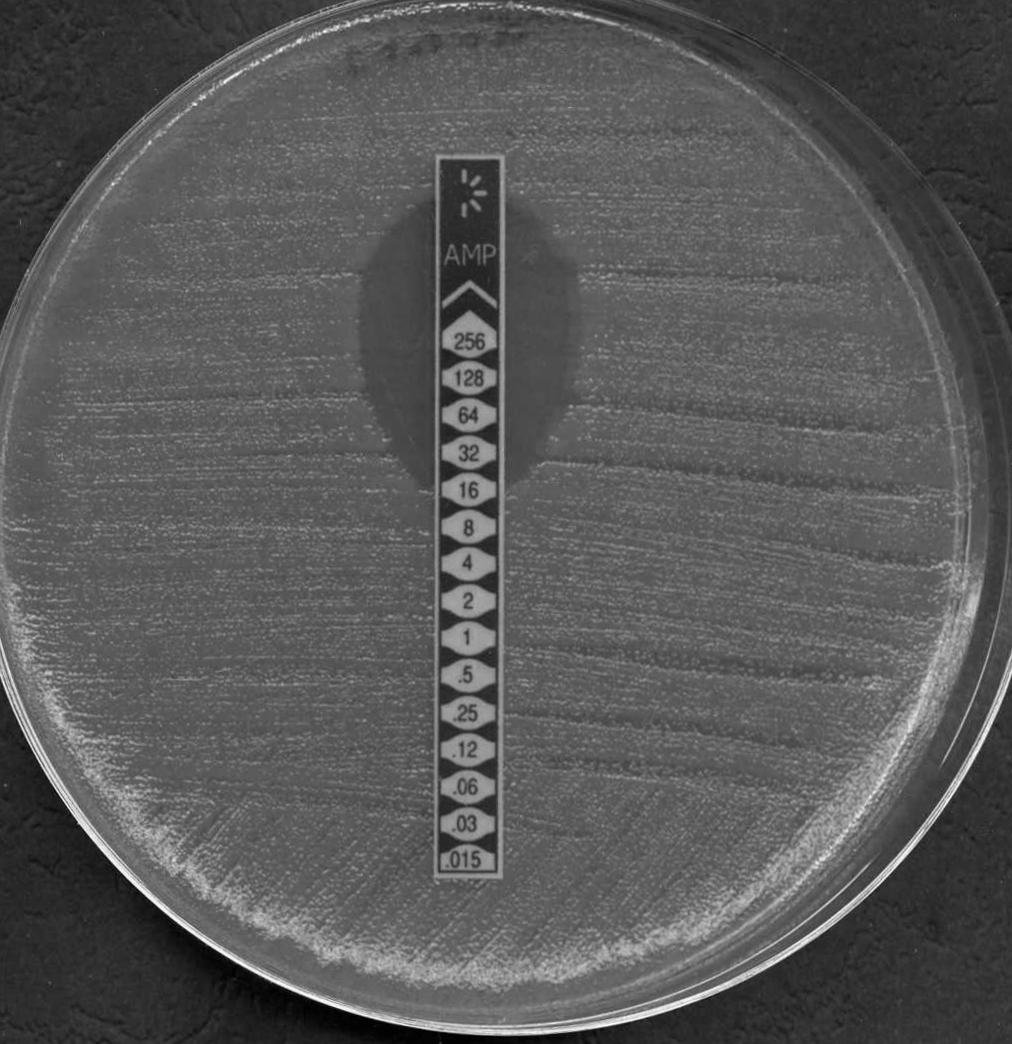

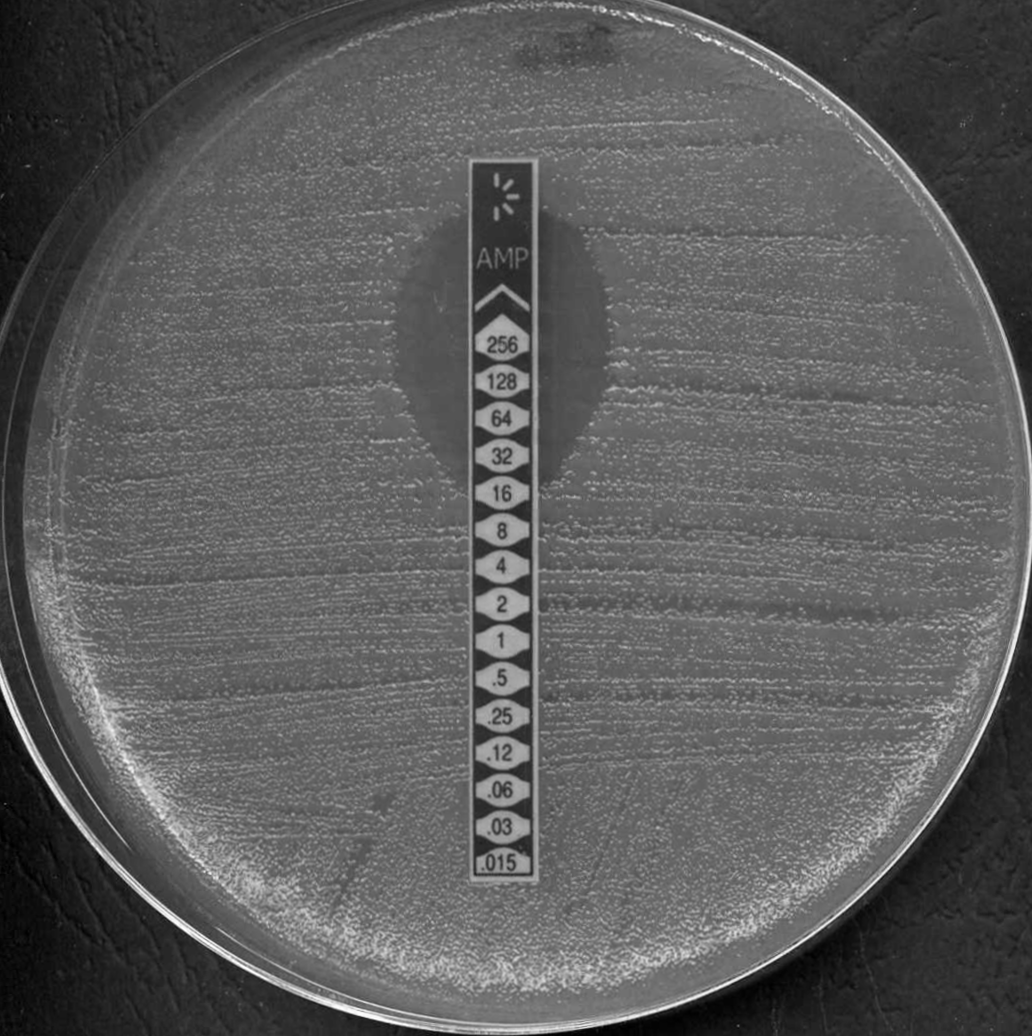

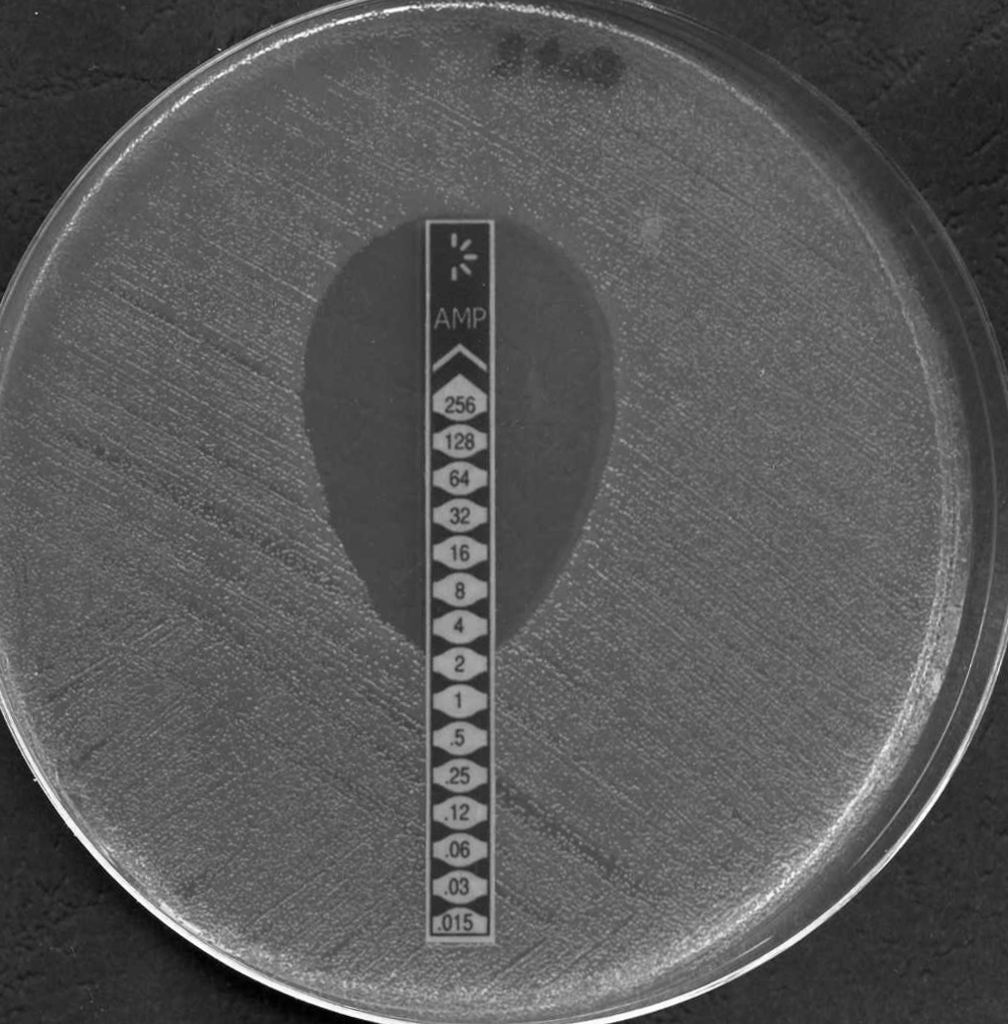

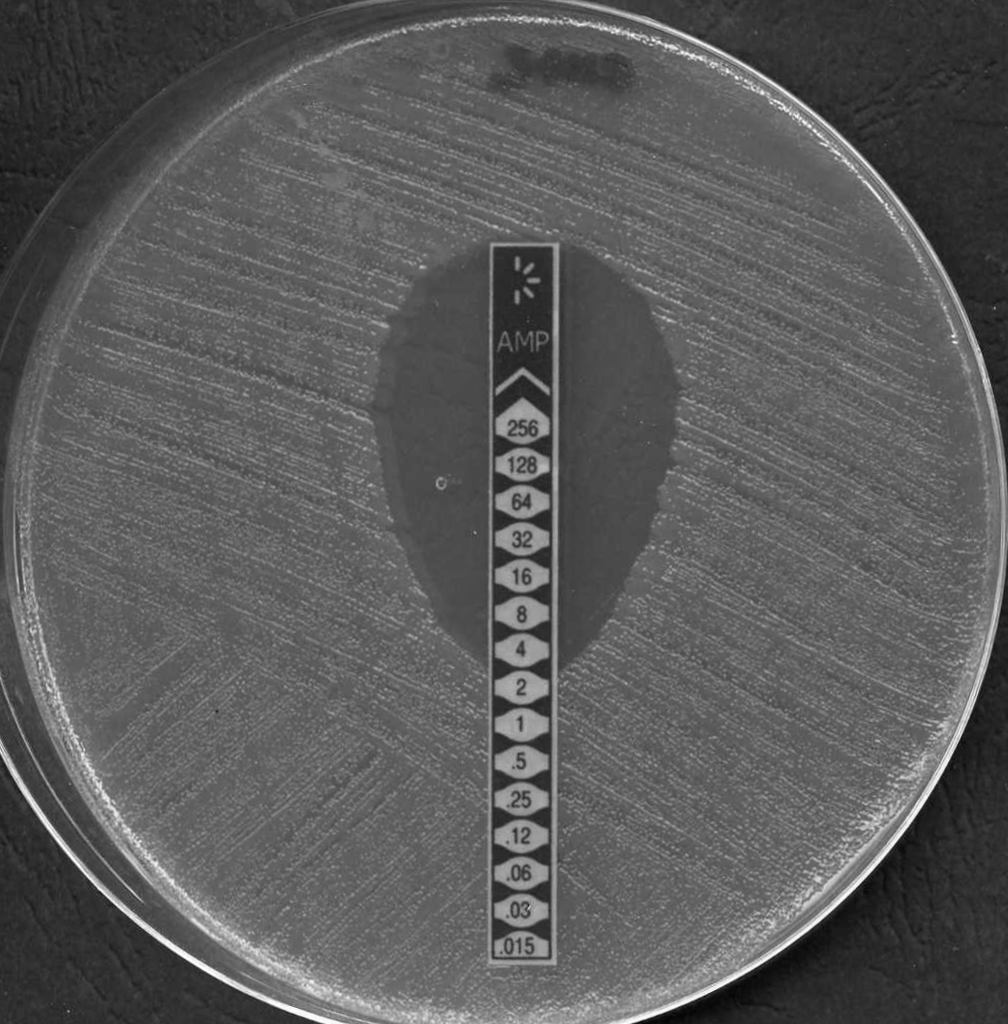

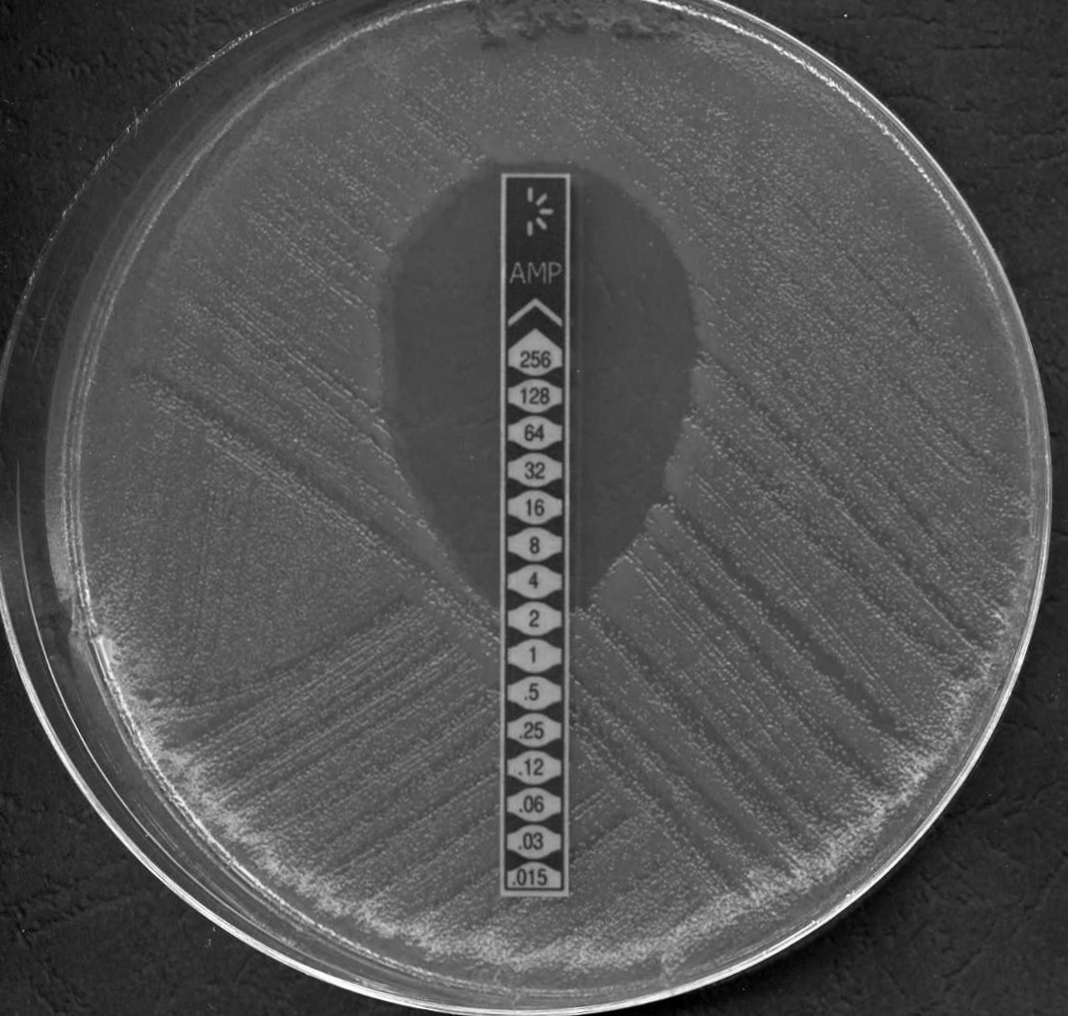

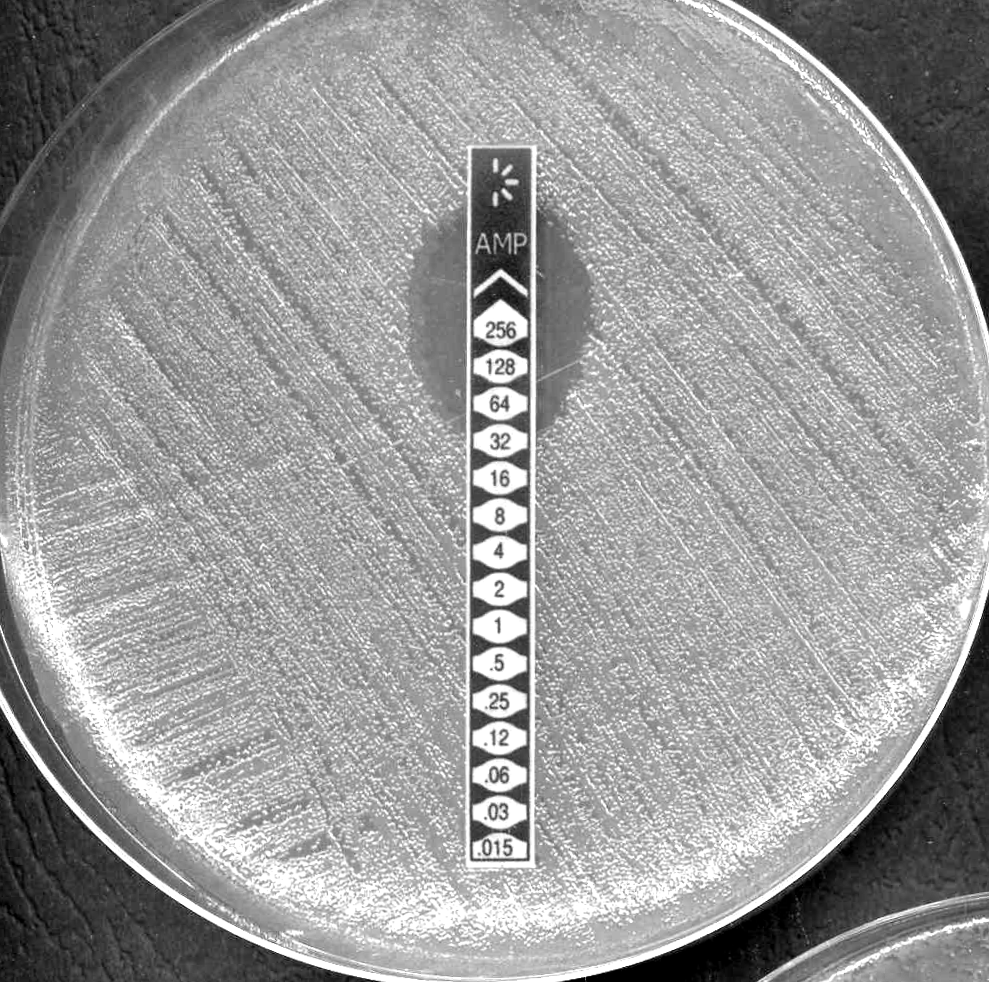

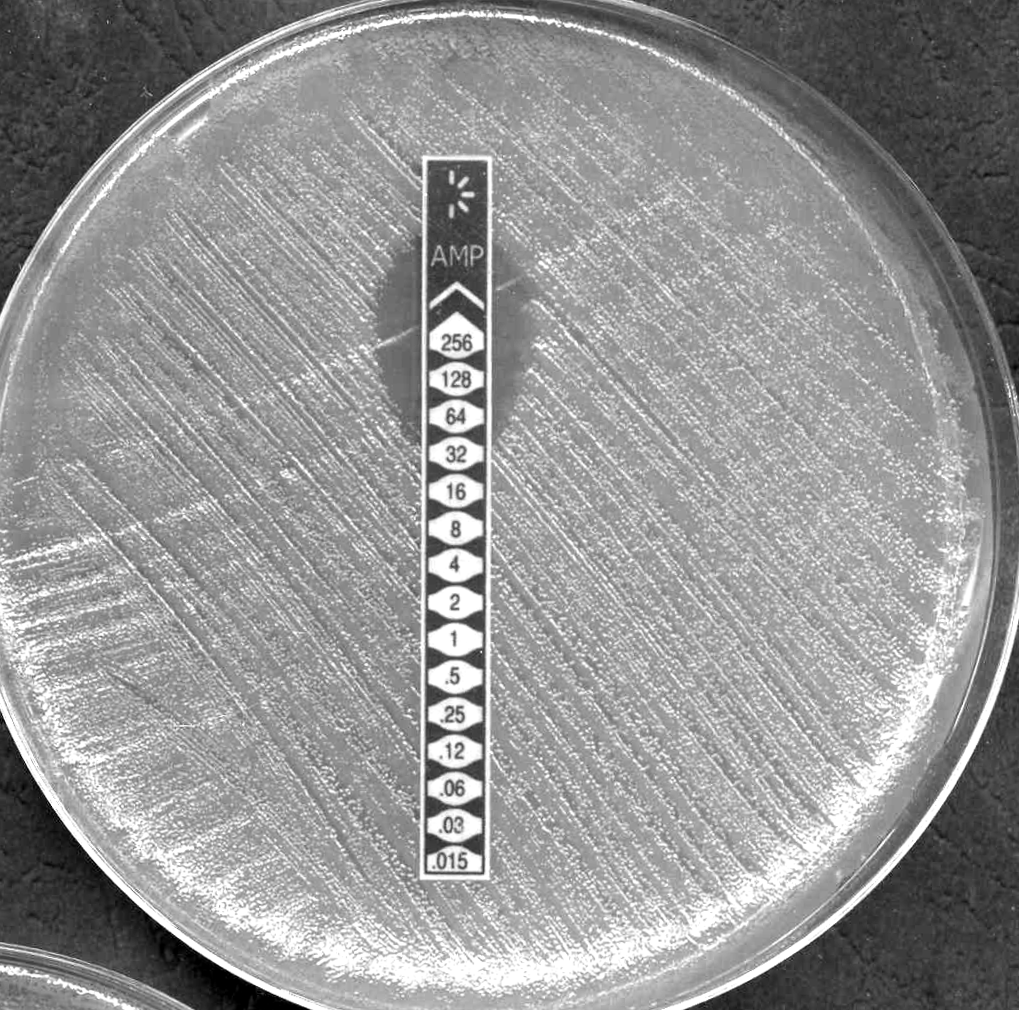

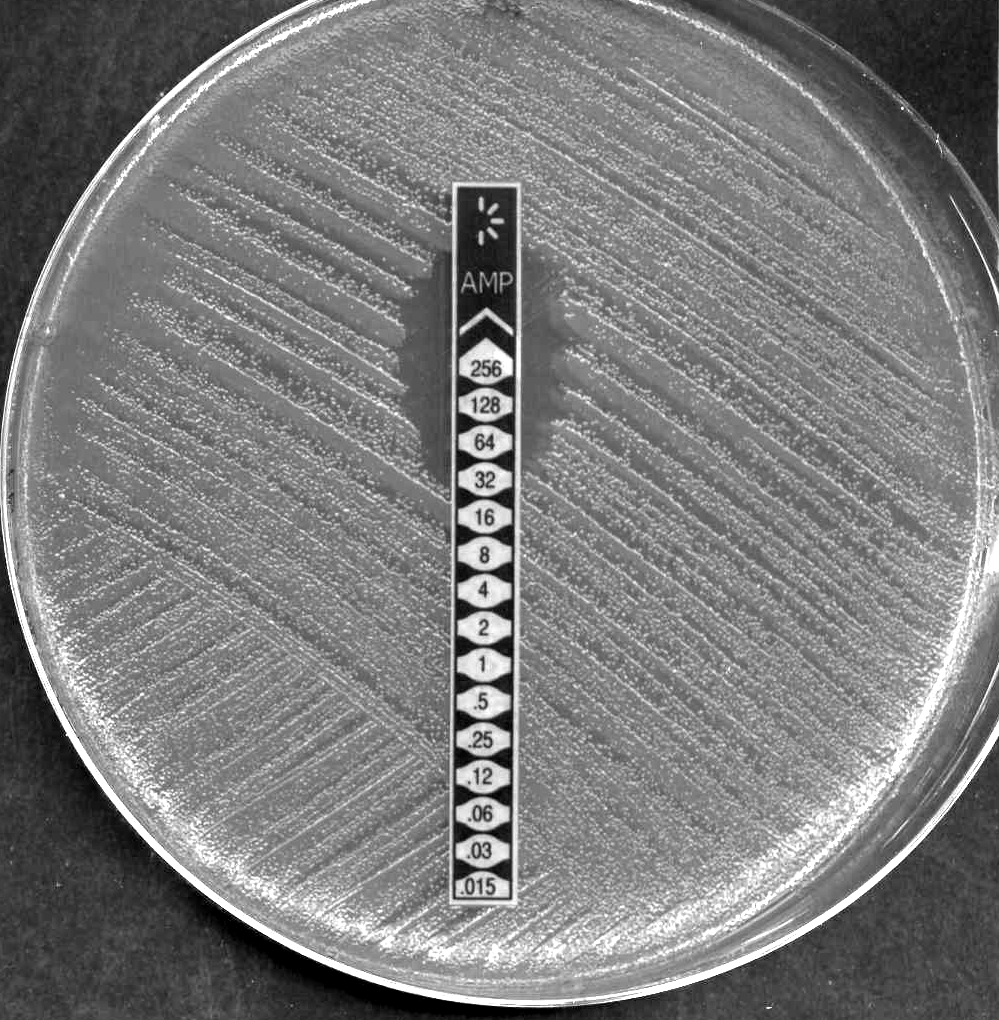

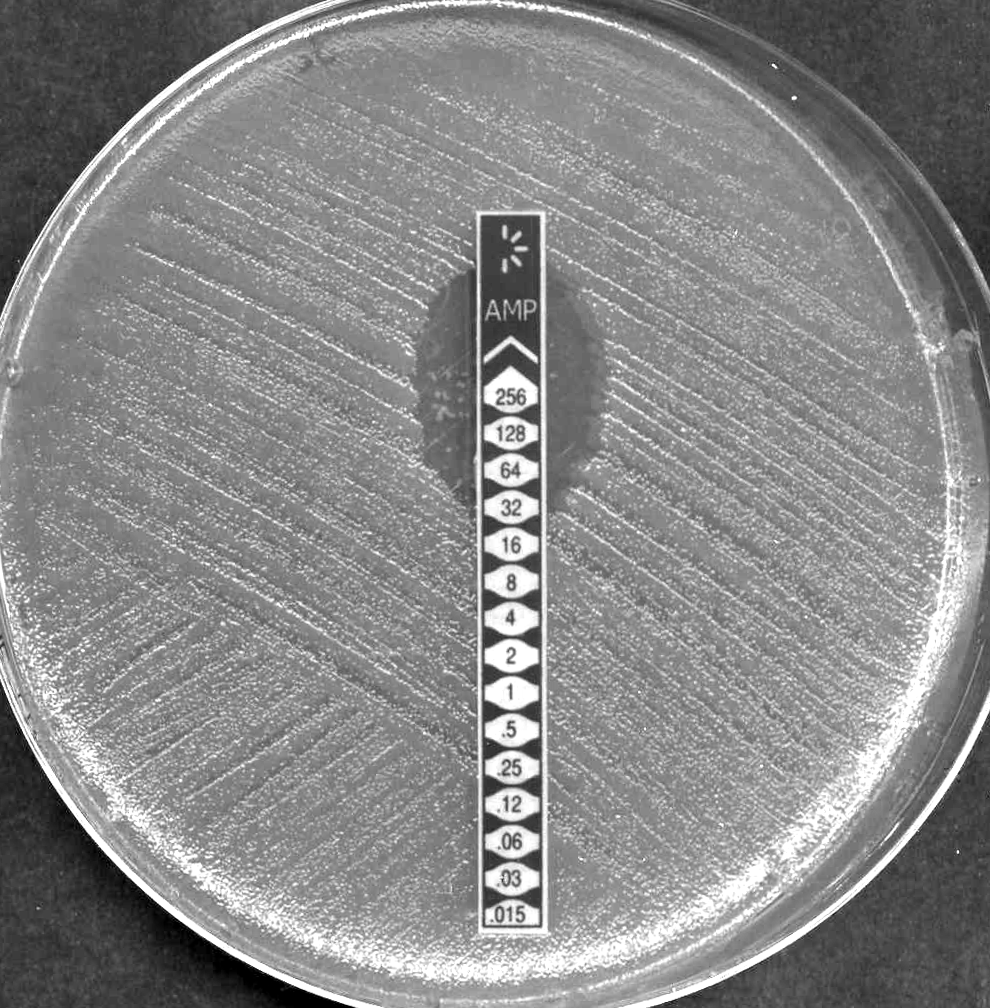

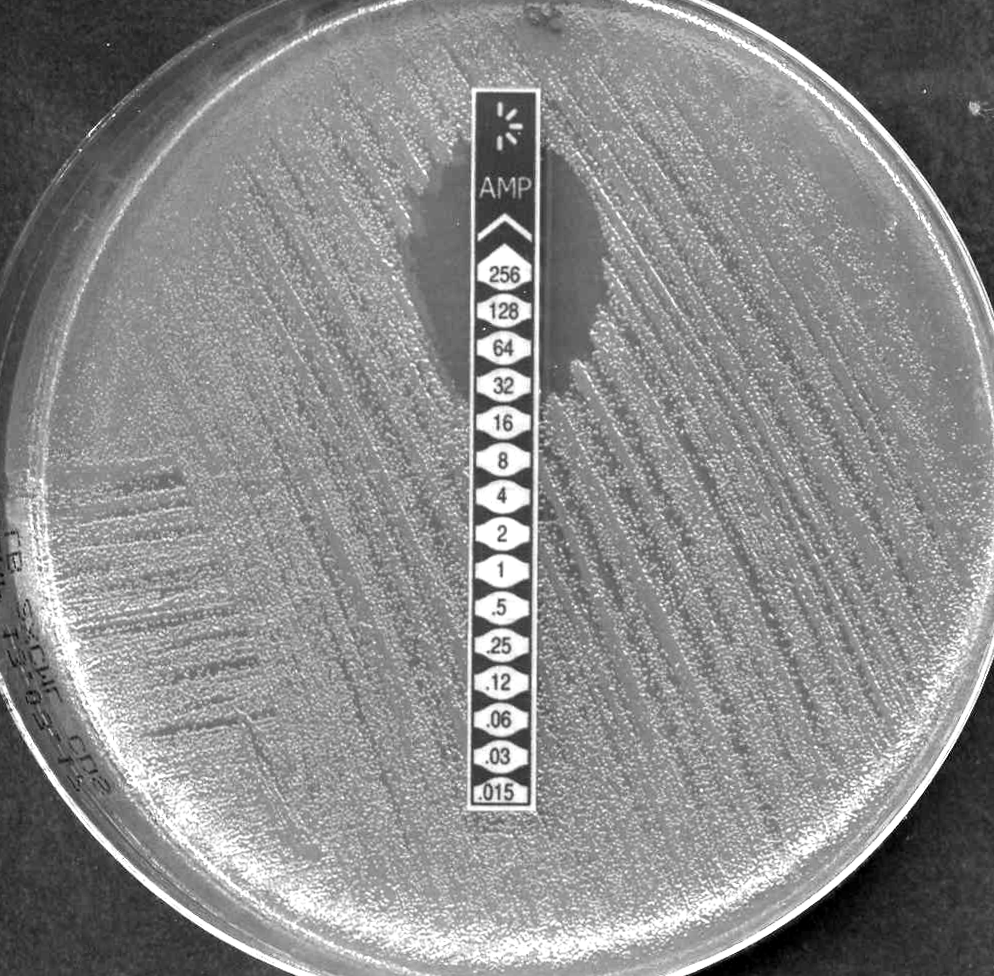

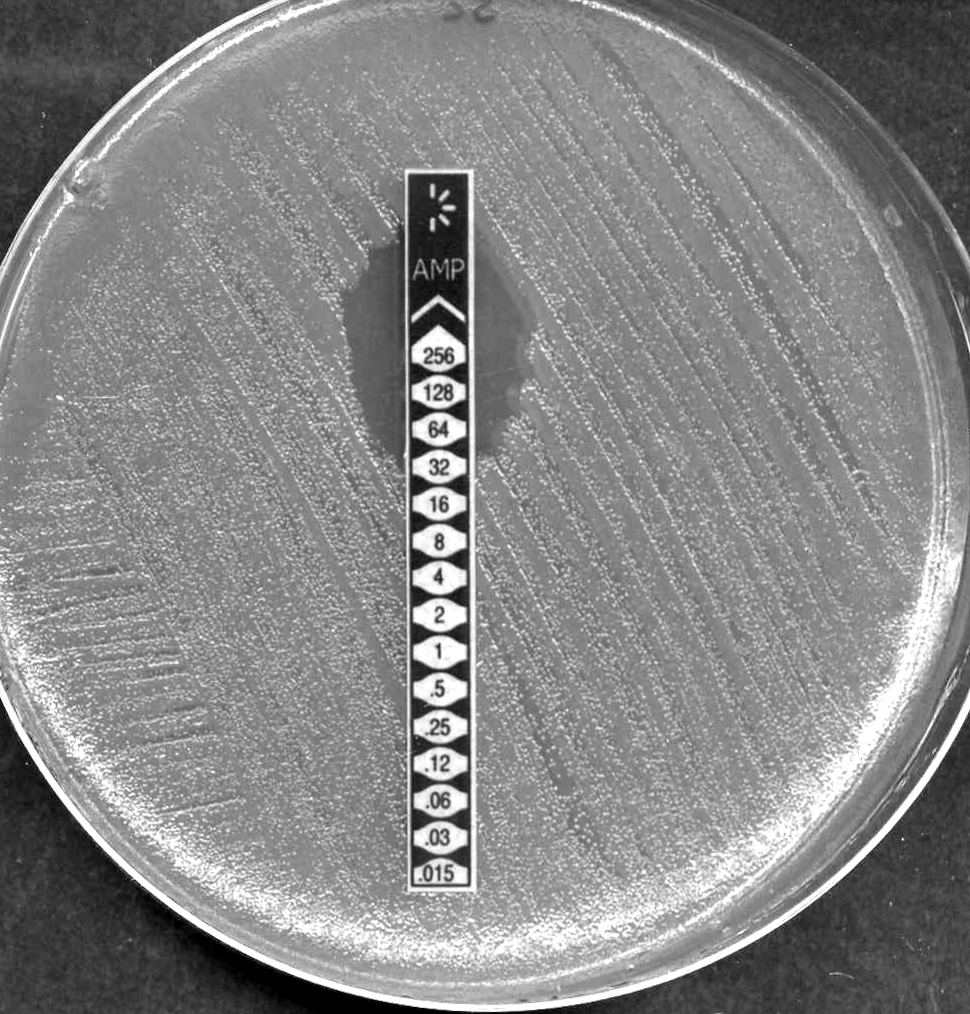

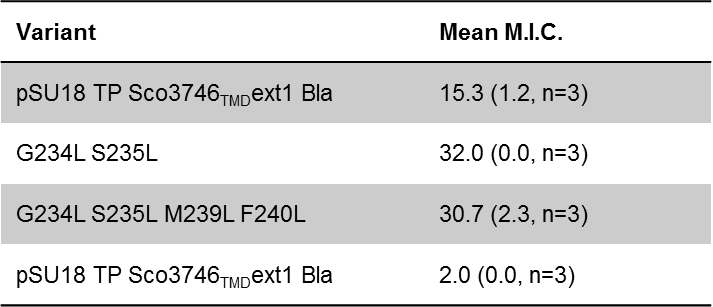


pSU18 TP Sco3746TMDext1 Bla

pSU18 TP Sco3746TMDext1 Bla

G234L S235L

G234L S235L M239L F240L

Supplement: Figure 9—source data 1. — DOI: http://dx.doi.org/10.7554/eLife.26577.024 [file elife-26577-fig9-data1.docx]

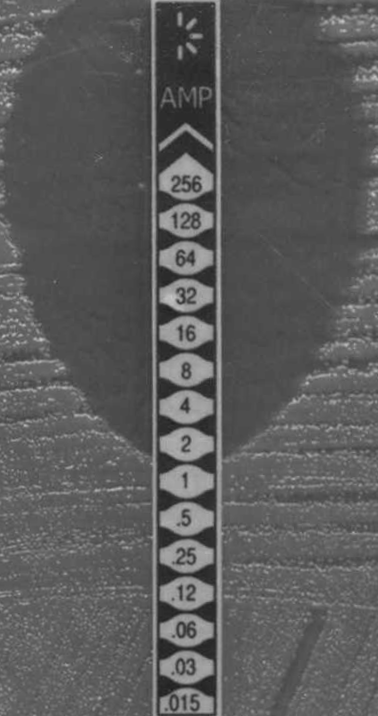

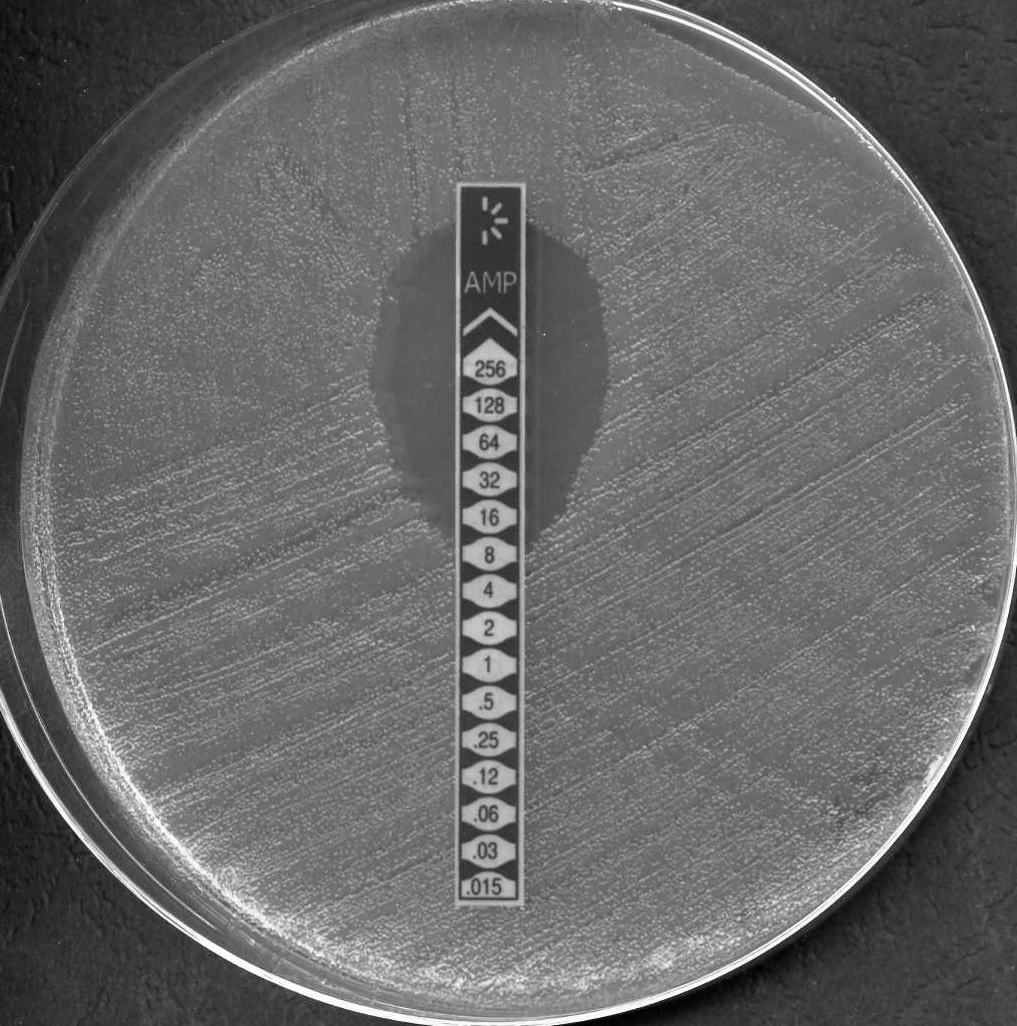

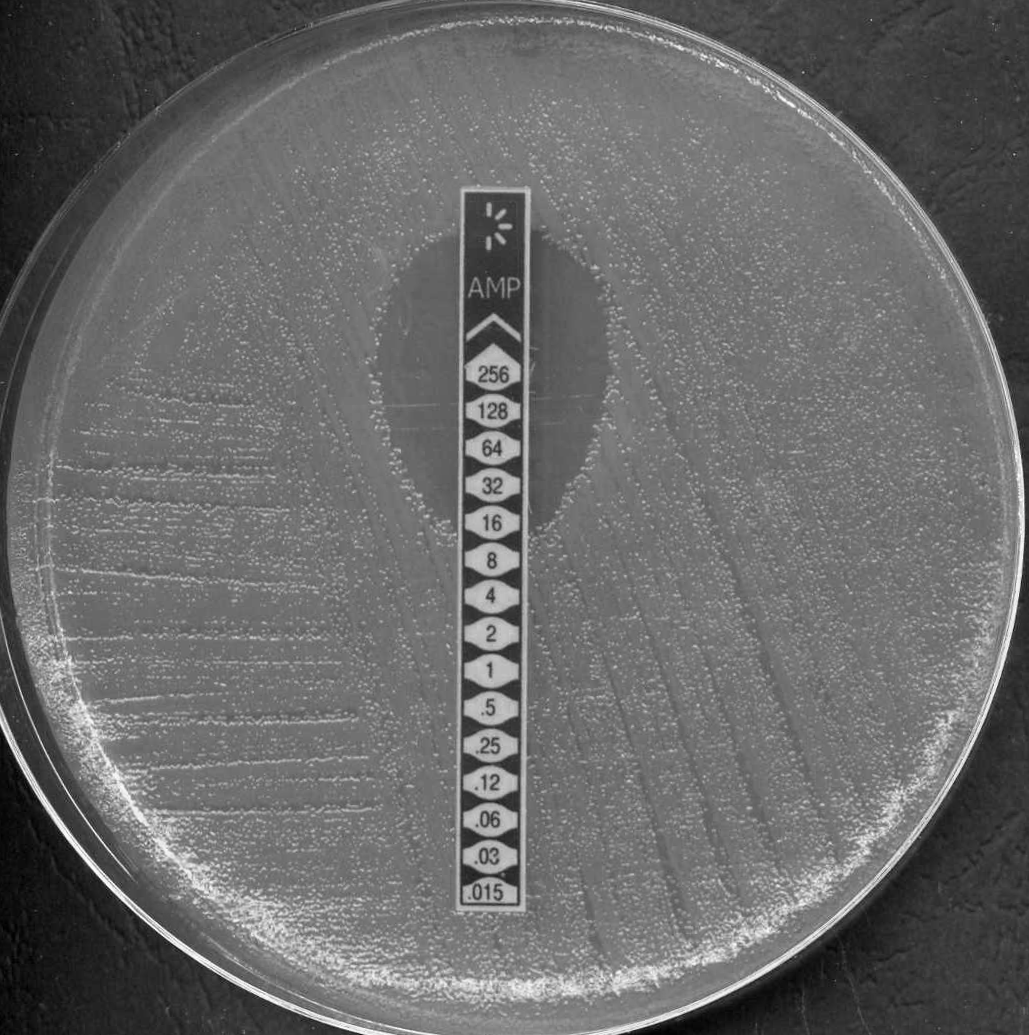

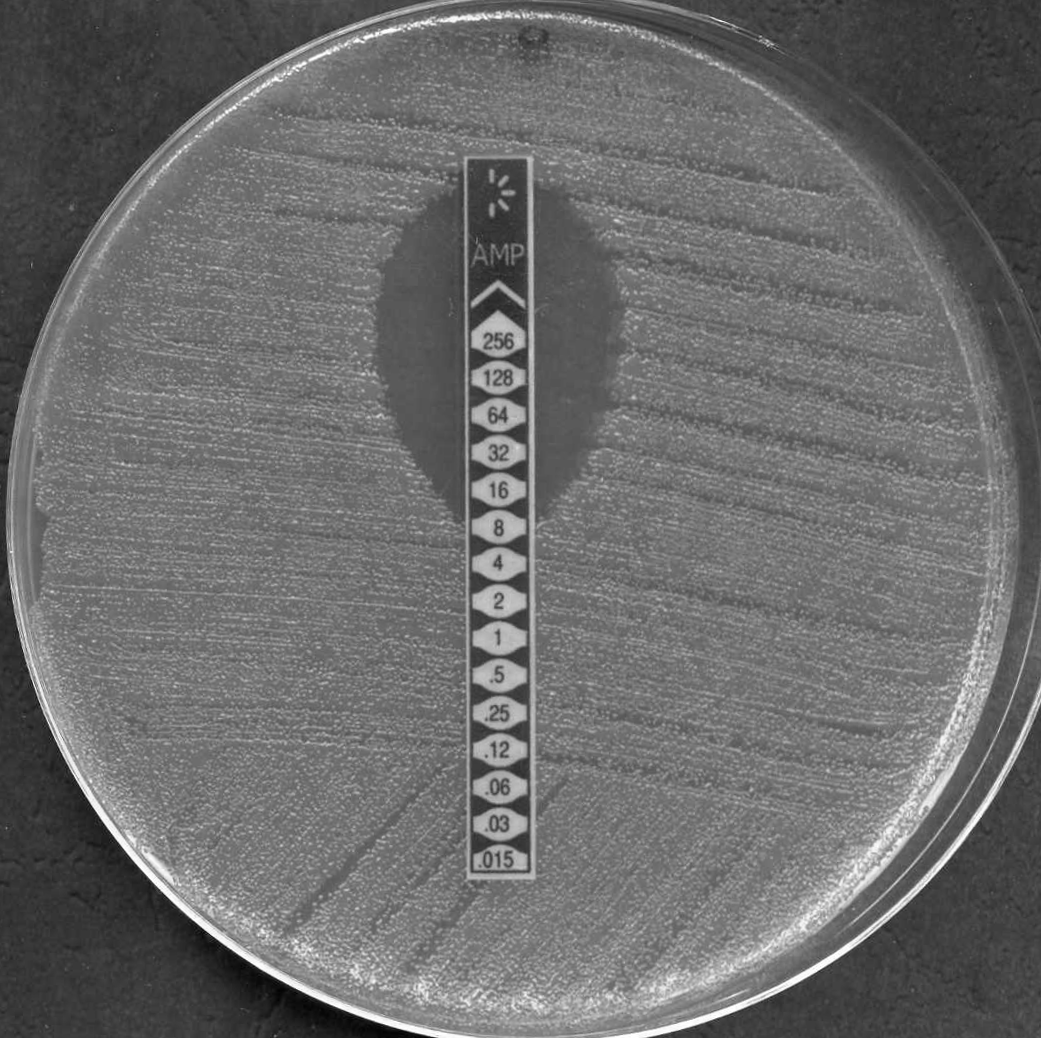

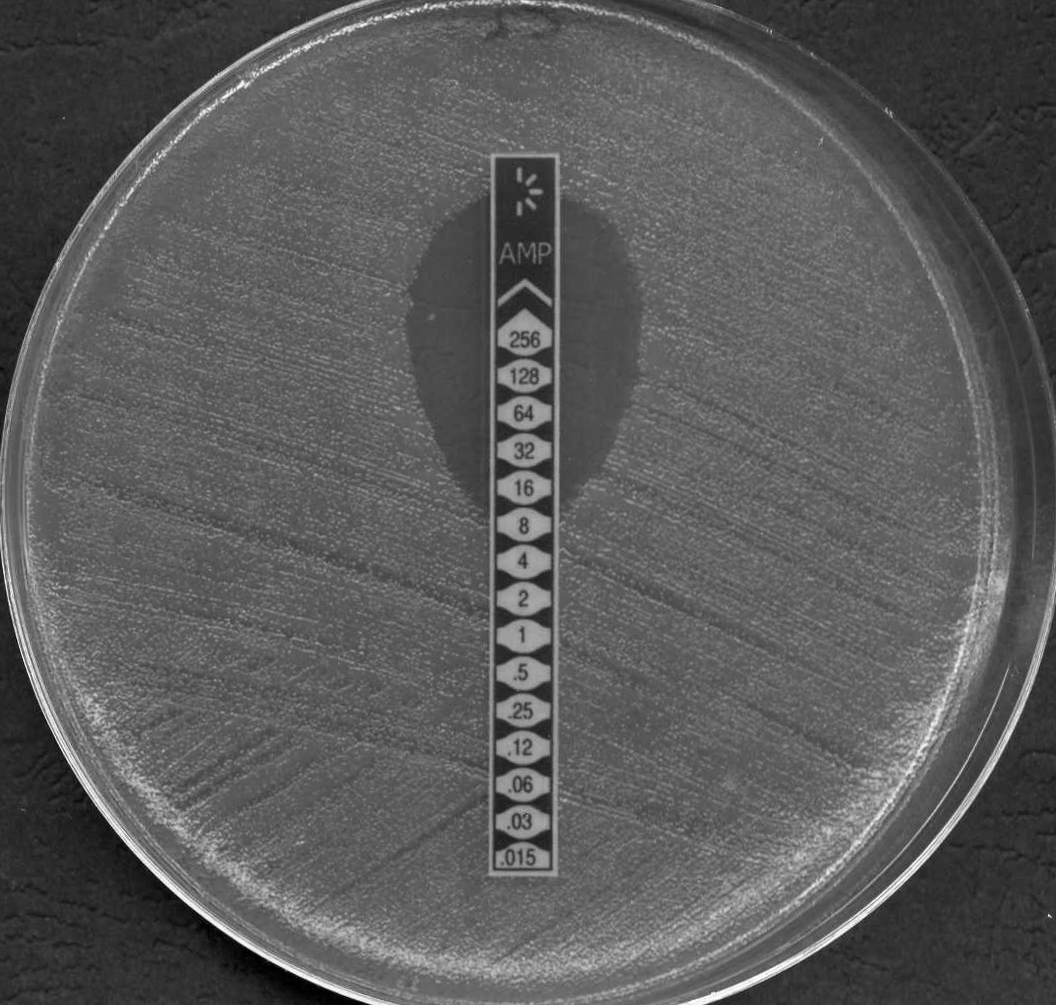

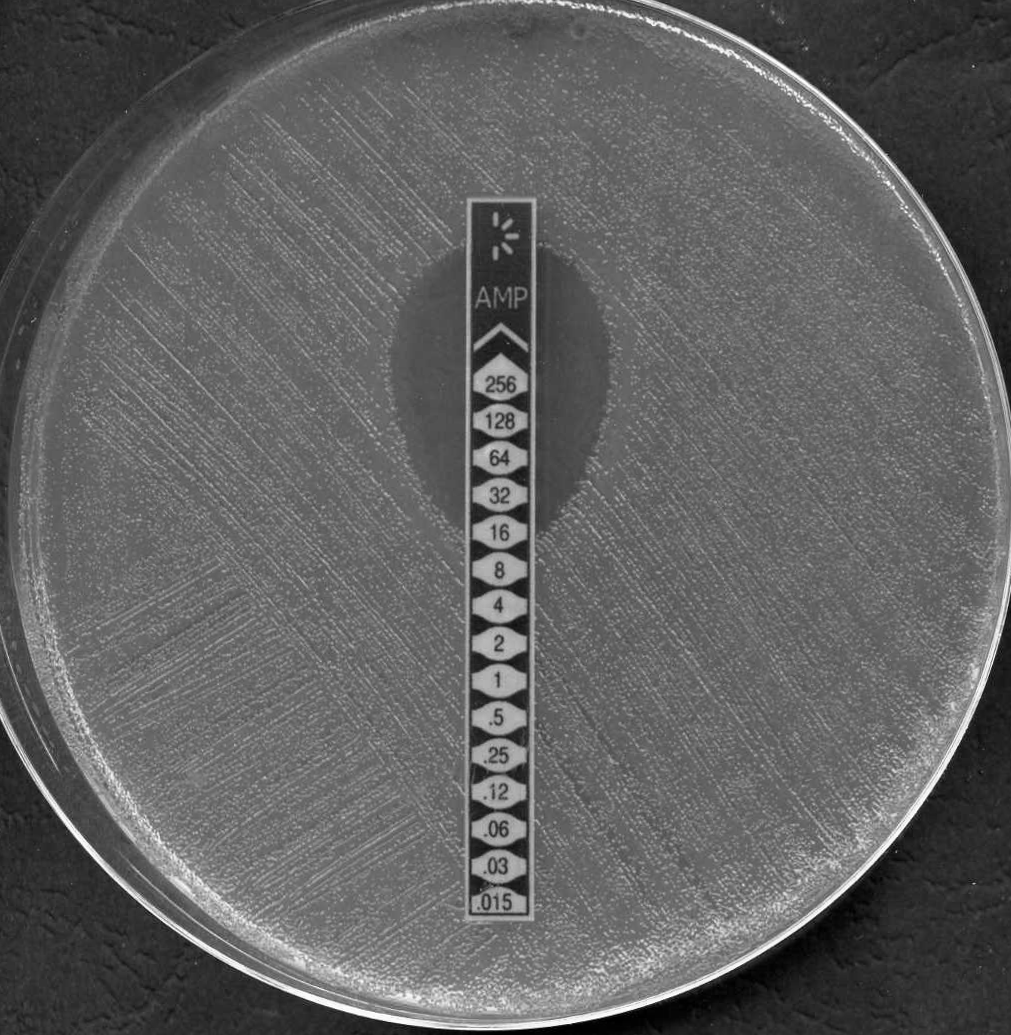

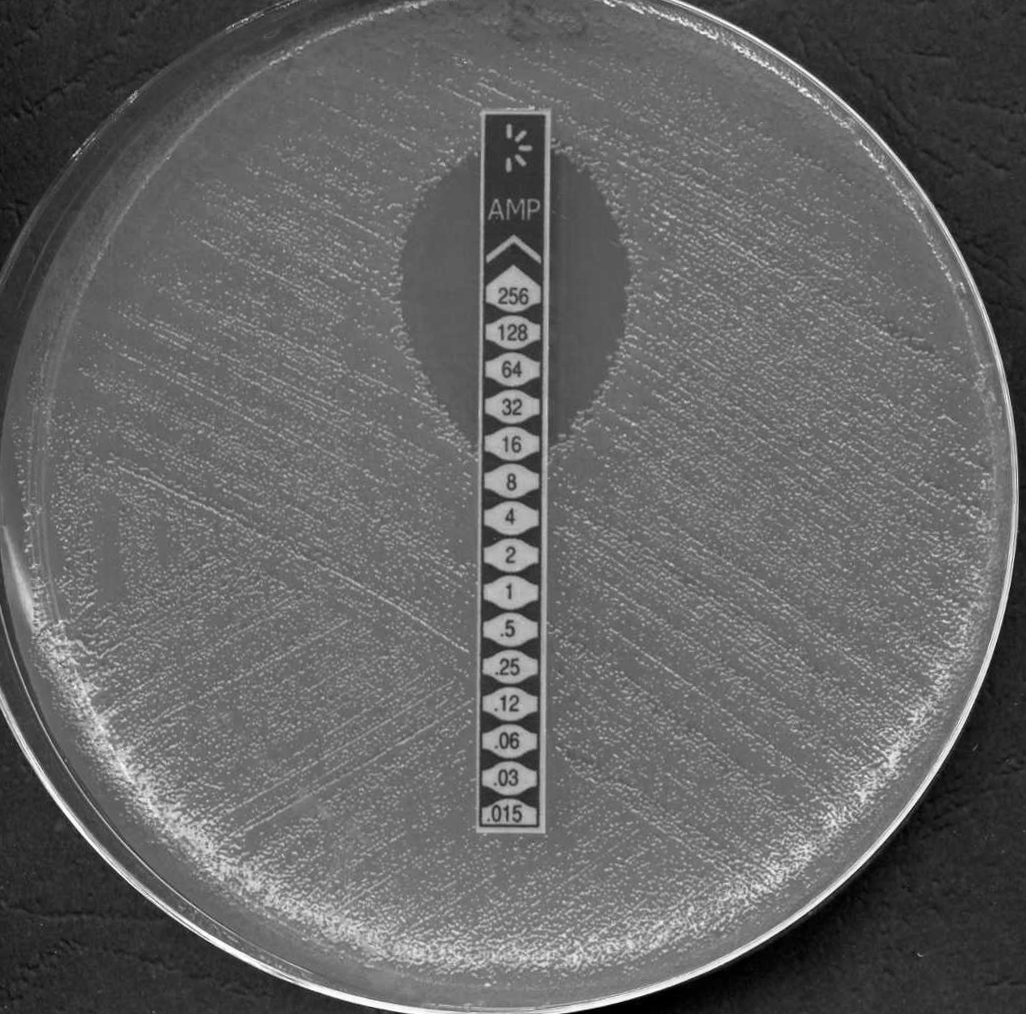

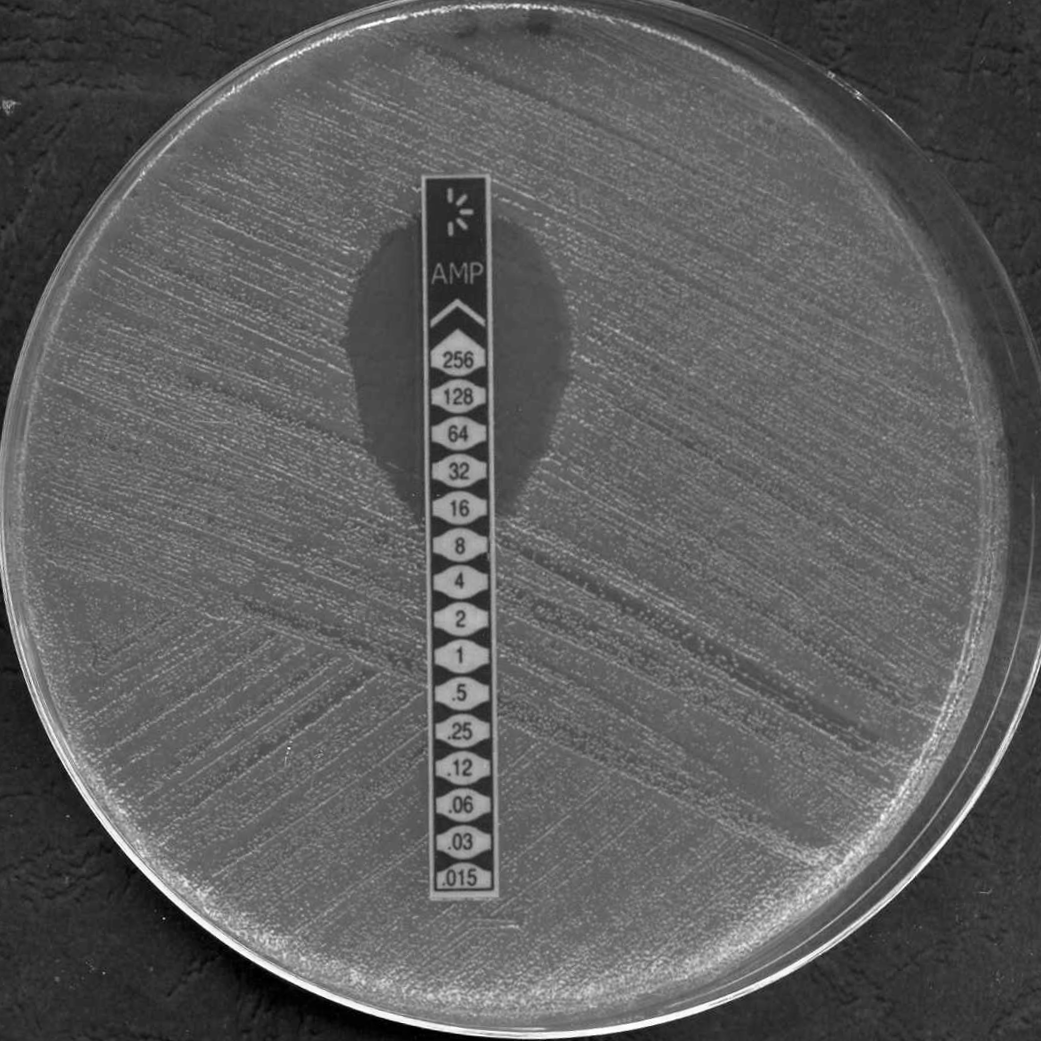

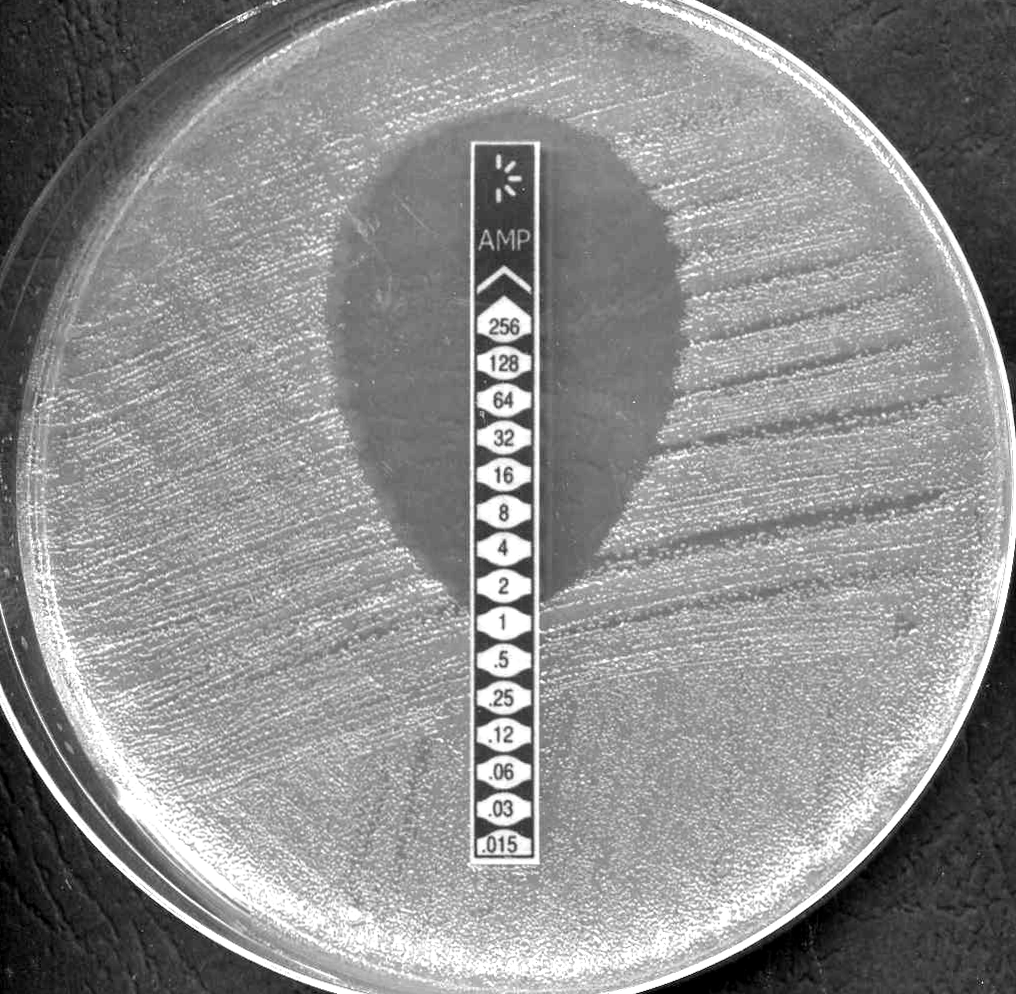

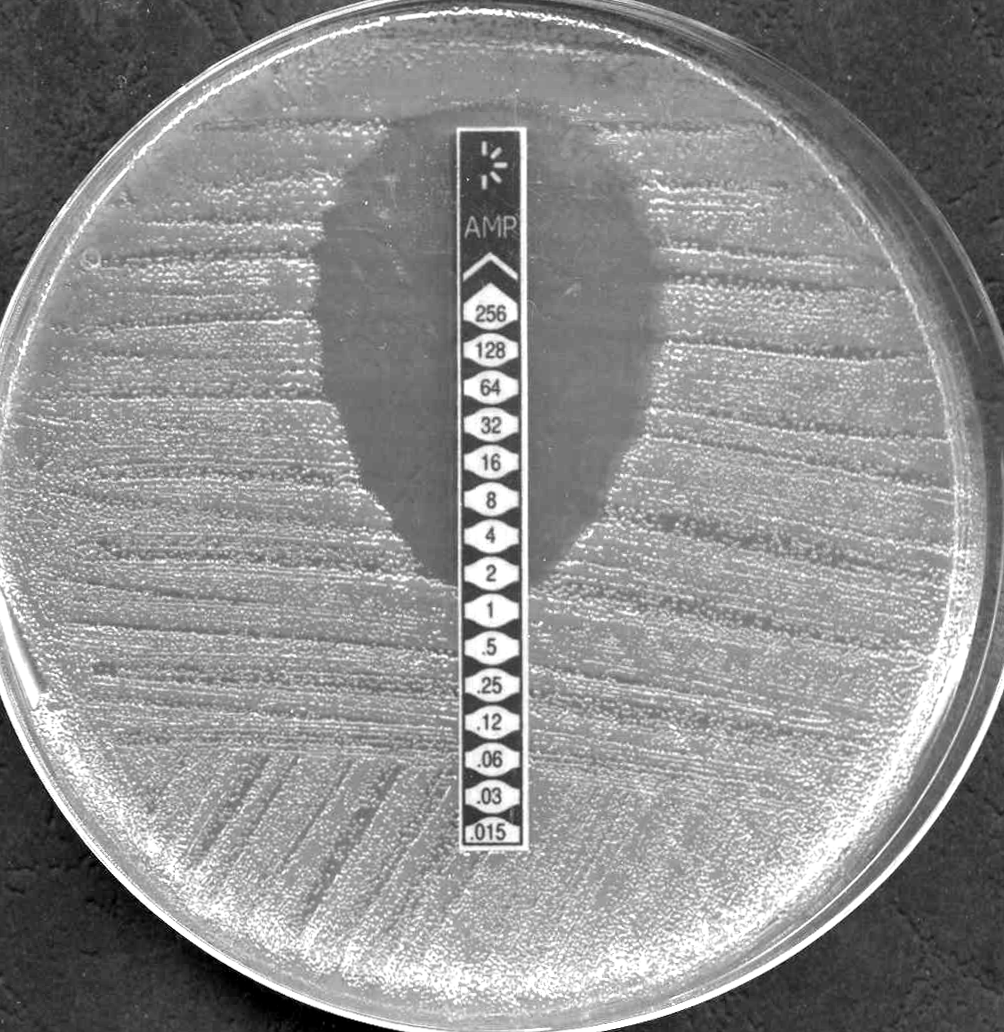

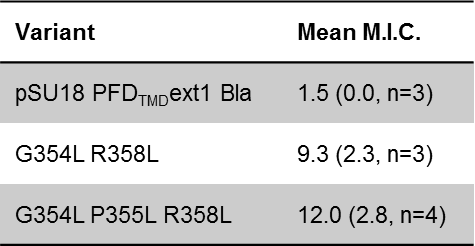


pSU18 PFDTMDext1 Bla

G354L R358L

G354L P355L R358L


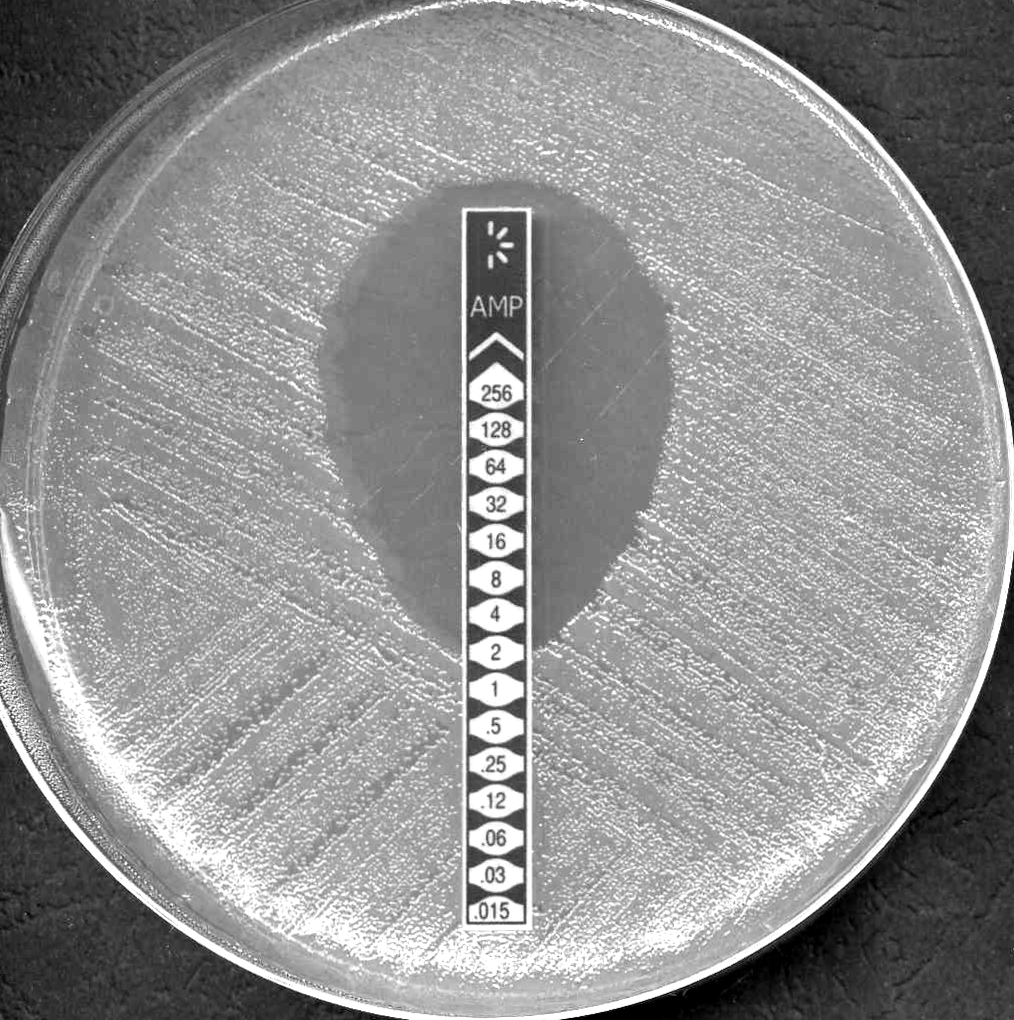

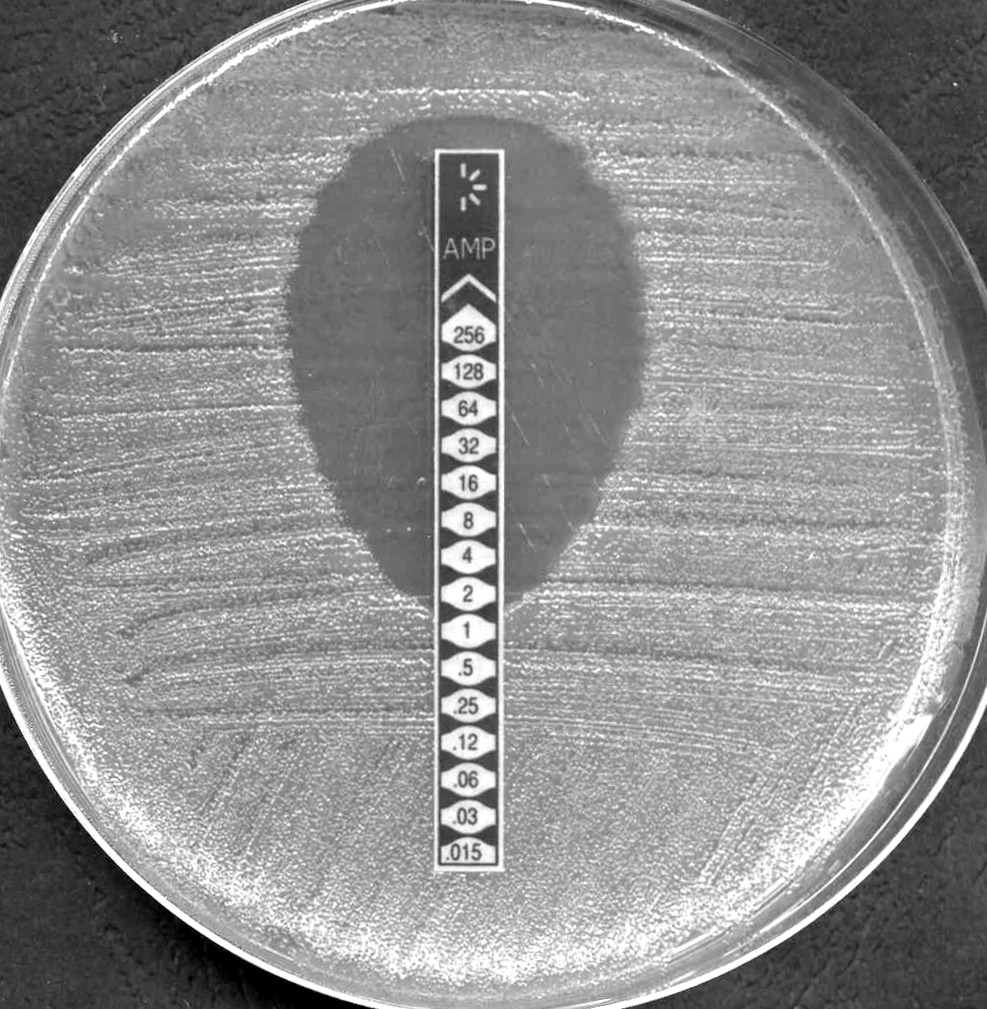

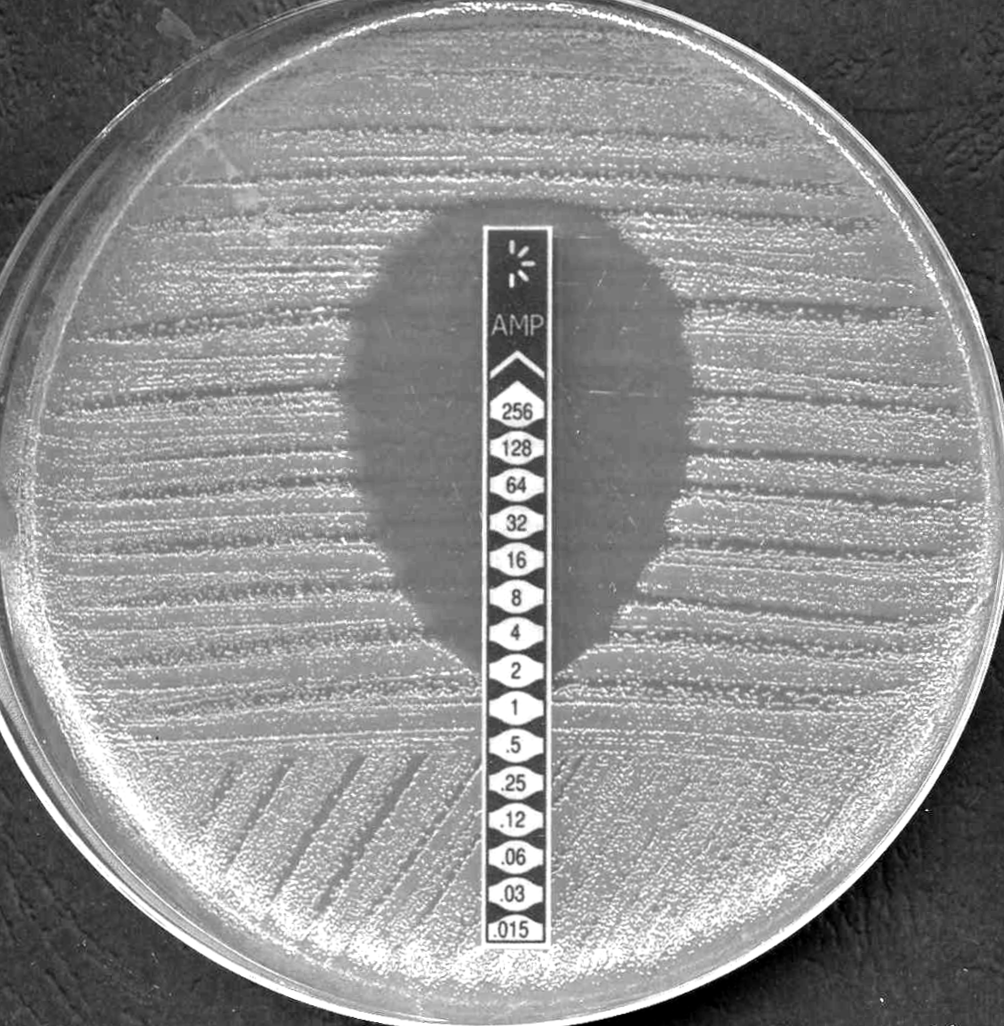

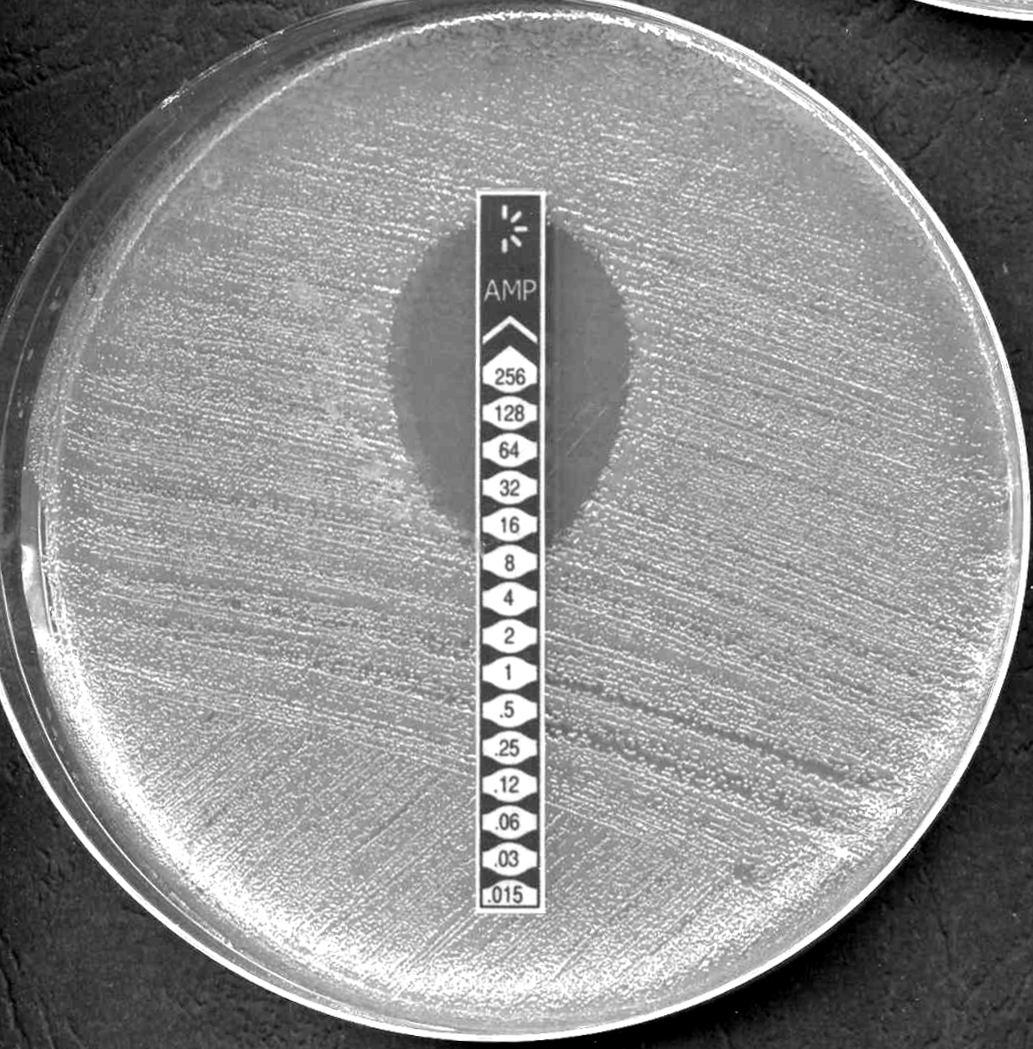

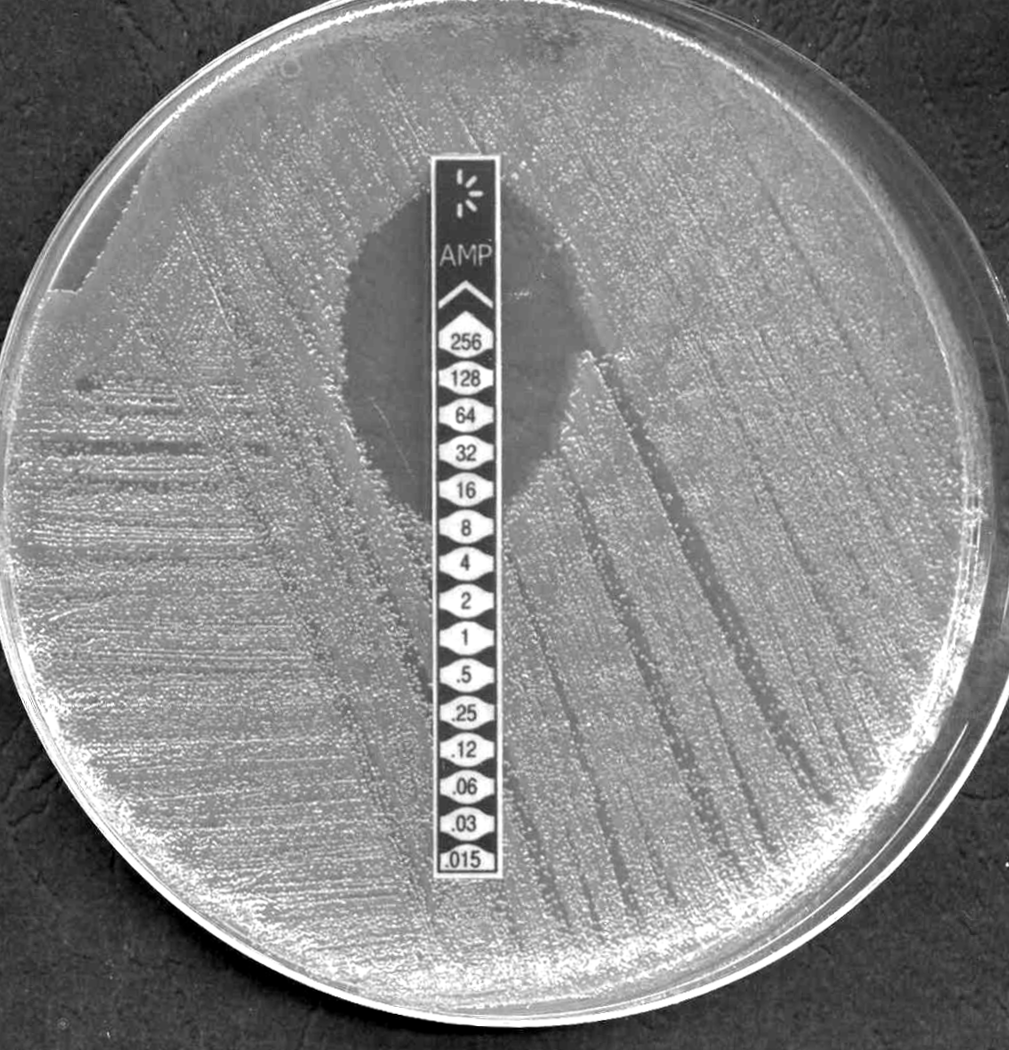

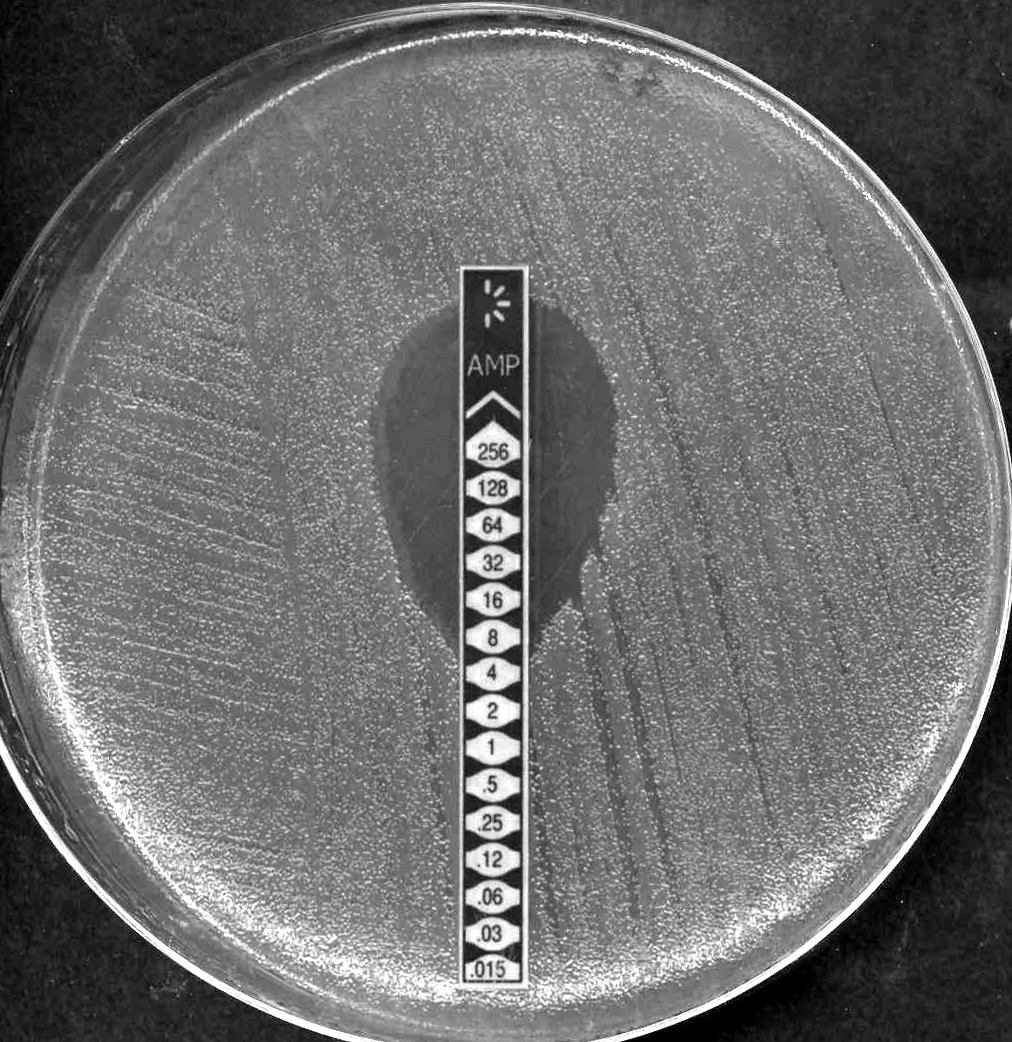

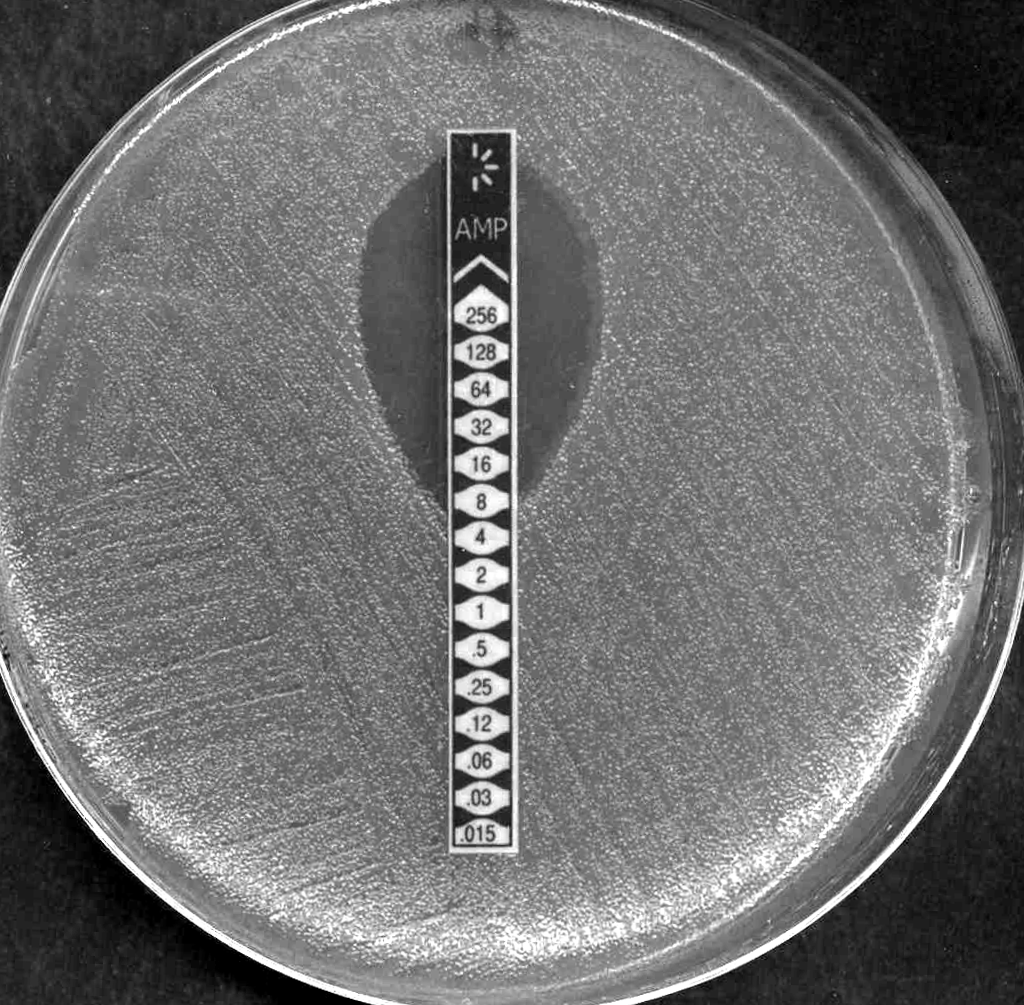

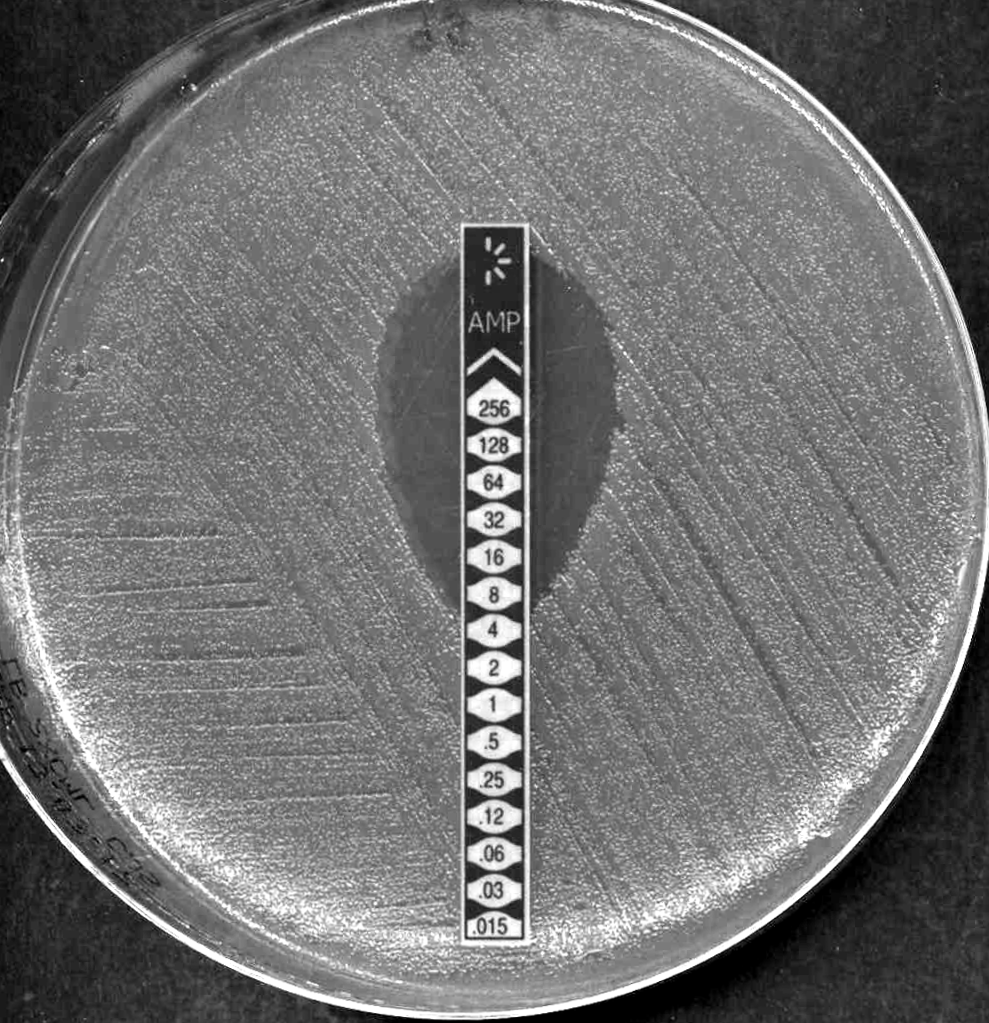

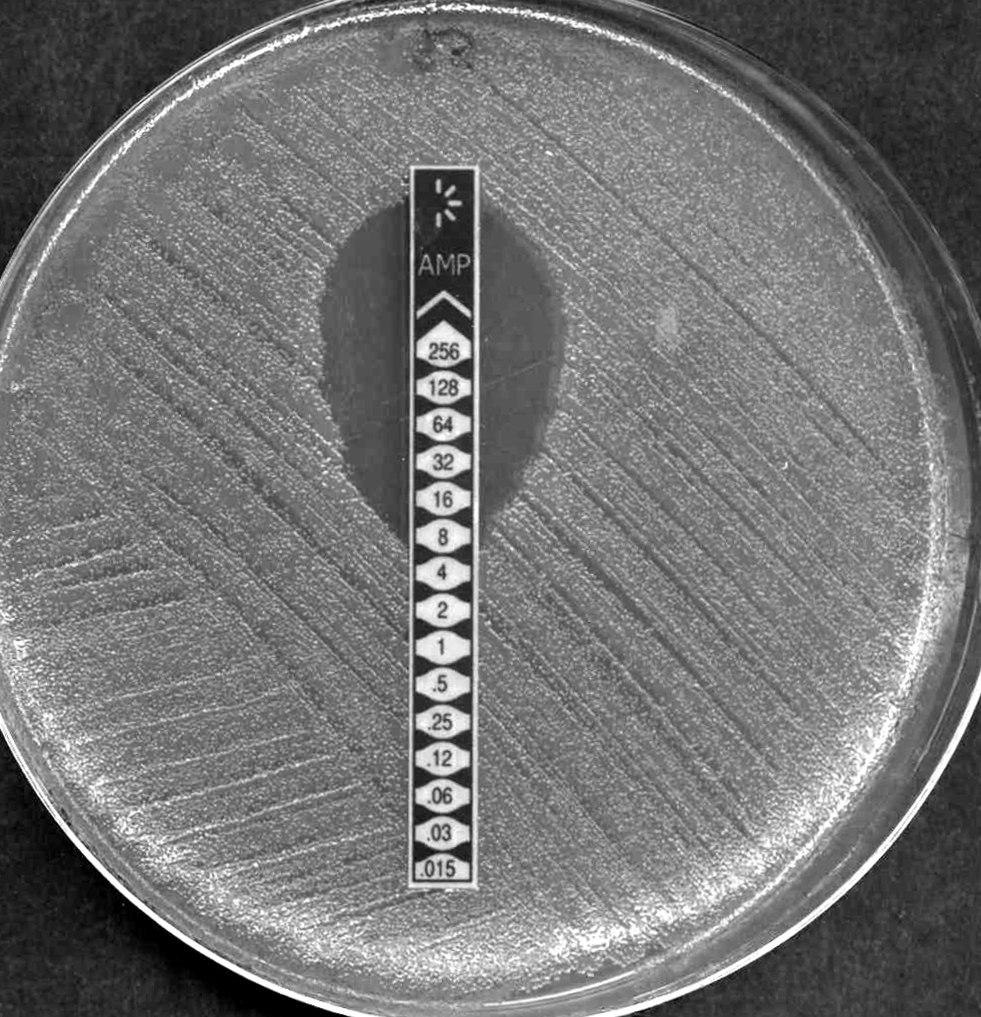

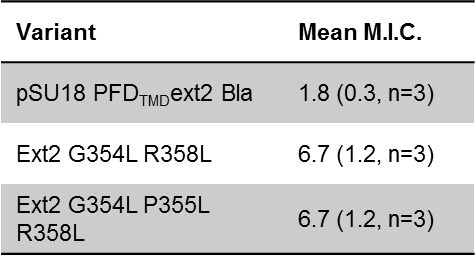


pSU18 PFDTMDext2 Bla

Ext2 G354L R358L

Ext2 G354L P355L R358L

Supplement: Figure 10—source data 1. — DOI: http://dx.doi.org/10.7554/eLife.26577.027 [file elife-26577-fig10-data1.docx]
