# Supplementary material for: A unifying mechanism for the biogenesis of membrane proteins co-operatively integrated by the Sec and Tat pathways
Source: eLife. 2017 May 17;6:e26577. doi: 10.7554/eLife.26577 (PMC5449189; doi:10.7554/eLife.26577)
Supplement: Figure 3—source data 1. — DOI: http://dx.doi.org/10.7554/eLife.26577.016 [file elife-26577-fig3-data1.docx]

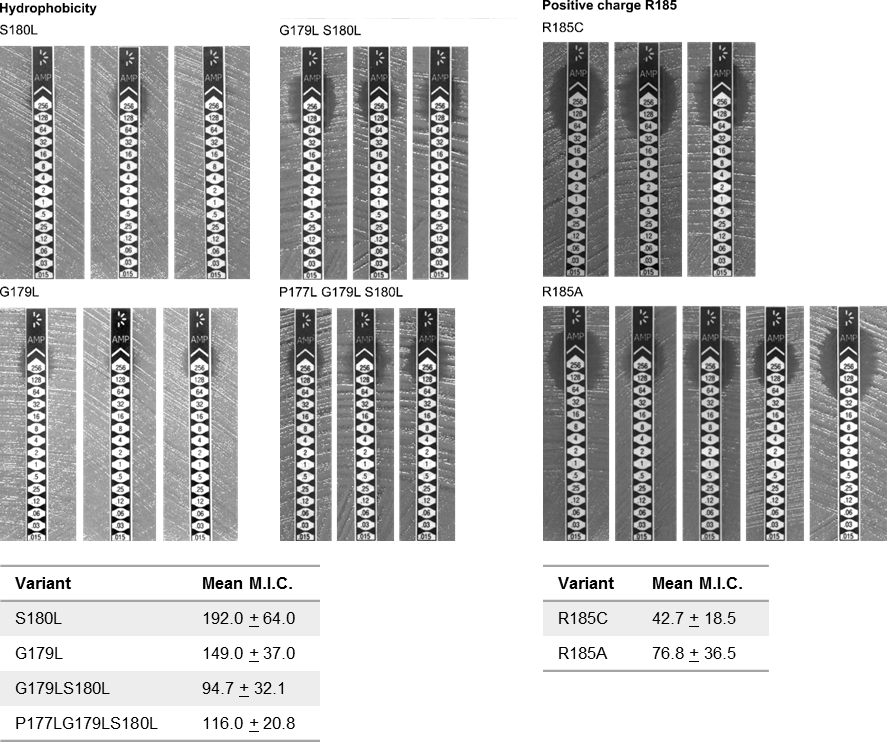

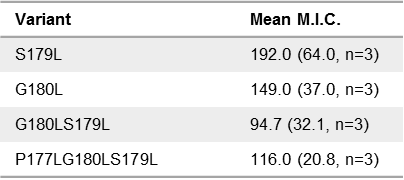

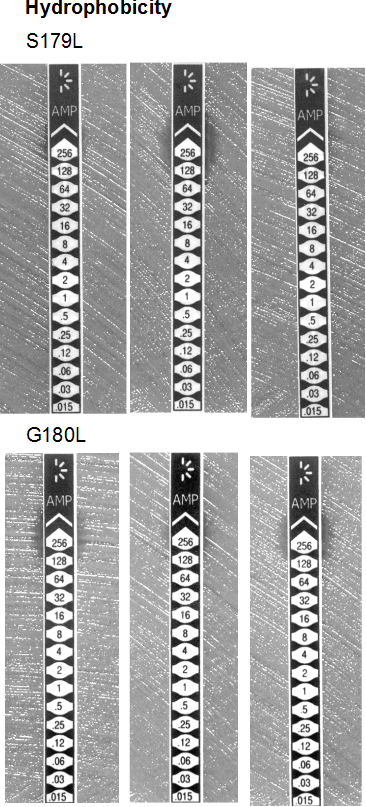

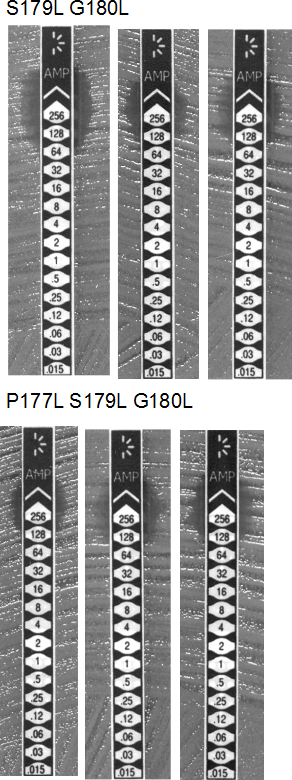


M.I.C.Evaluator^TM^ strip test of DADE (*tat*^-^) harbouring the pSUPROM vector encoding hydrophobicity (G180L-Rieske-Bla, S179L-Rieske-Bla, S179LG180L-Rieske-Bla and P177LS179LG180L-Rieske-Bla) or R185 variants (R185C-Rieske-Bla, and R185A-Rieske-Bla,). Stationary phase cultures were diluted to OD_600_ 0.1 and a lawn of bacteria was spread onto LB agar plates, M.I.C.Evaluator^TM^ strips were placed on the lawn and the plate was incubated at 37°C for 18 h. The M.I.C. (μg/ml) for ampicillin is read at the intersection of the test strip and the clearing of bacteria. The tables indicate the mean M.I.C. and ± s.d.


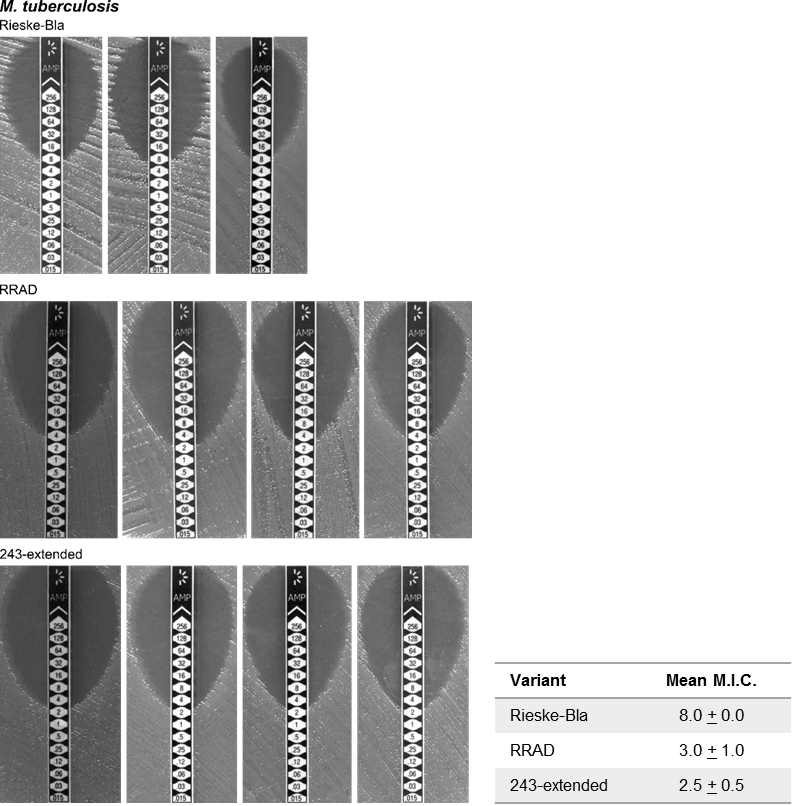


M.I.C.Evaluator^TM^ strip test of DADE (*tat*^-^) harbouring the pSUPROM vector encoding *M. tuberculosis* variants (Rieske-Bla, RRAD-Rieske-Bla and 243-extended-Rieske-Bla,). Stationary phase cultures were diluted to OD_600_ 0.1 and a lawn of bacteria was spread onto LB agar plates, M.I.C.Evaluator^TM^ strips were placed on the lawn and the plate was incubated at 37°C for 18 h. The M.I.C. (μg/ml) for ampicillin is read at the intersection of the test strip and the clearing of bacteria. The table indicates the mean M.I.C. and ± s.d.
