# Supplementary material for: A unifying mechanism for the biogenesis of membrane proteins co-operatively integrated by the Sec and Tat pathways
Source: eLife. 2017 May 17;6:e26577. doi: 10.7554/eLife.26577 (PMC5449189; doi:10.7554/eLife.26577)
Supplement: Supplementary file 1. — DOI: http://dx.doi.org/10.7554/eLife.26577.030 [file elife-26577-supp1.docx]

**Supplementary file 1A. Strains used in this work.**

| Strain | Genotype | Reference |
| --- | --- | --- |
| MC4100 | F^-^Δ*lac*U169 *araD139 rpsL150 relA1 ptsF rbs flbB5301* | (56) |
| DADE | As MC4100, ∆*tatABCD* ∆*tatE* | (57) |
| MCDSSAC | As MC4100, *amiA*∆2-33, *amiC*∆2-32 | (26) |
| MCDSSACΔtat | As MCDSSAC, ∆*tatABC*::Apra | (22) |
| HS3018-A | F^-^ Δ*lac*U169 *araD*139 *rpsL thi MalTc*-1 Δ*malE*444, Ara^R^ | (58) |
| HS3018-AΔtat | As HS3018-A, ∆*tatABCD* ∆*tatE* | (58) |
| DH5α | F^-^ *endA1* *gln*V44 *thi*-1 *recA1* *relA1* *gyrA96* *deoR* nupG Φ80d*lacZ*ΔM15 Δ(*lacZYA-argF*)U169, *hsdR17*(r_K_^-^ m_K_^+^), λ– | Stratagene |

**Supplementary file 1B. Amino acid sequences of the fusion proteins used in this study.**

**Rieske_TMD_-Bla**

MSSQDIPEENLPAEQDRPHGAAARPADETNPFADPGLPPHEPRVQDVDERAAKRSERTVALLFTLSMLATIAFIAAFVAIDVDKSVYIFPLGHISALNFALGMTLGVALFAIGAGAVHWARTLMSDEEVADERHPIEASPEVRAKVHADFKQGAKESVIGRRKLIRNTMLGALTLVPLSGVVLLRSRHPETLVKVKDAEDQLGARVGYIELDLNSGKILESFRPEERFPMMSTFKVLLCGAVLSRVDAGQEQLGRRIHYSQNDLVEYSPVTEKHLTDGMTVRELCSAAITMSDNTAANLLLTTIGGPKELTAFLHNMGDHVTRLDRWEPELNEAIPNDERDTTMPAAMATTLRKLLTGELLTLASRQQLIDWMEADKVAGPLLRSALPAGWFIADKSGAGERGSRGIIAALGPDGKPSRIVVIYTTGSQATMDERNRQIAEIGASLIKHW

**Rieske_TMD_-Bla 205 extended**

MSSQDIPEENLPAEQDRPHGAAARPADETNPFADPGLPPHEPRVQDVDERAAKRSERTVALLFTLSMLATIAFIAAFVAIDVDKSVYIFPLGHISALNFALGMTLGVALFAIGAGAVHWARTLMSDEEVADERHPIEASPEVRAKVHADFKQGAKESVIGRRKLIRNTMLGALTLVPLSGVVLLRDLGPLPGTKLRHTLWSKGKLSRHPETLVKVKDAEDQLGARVGYIELDLNSGKILESFRPEERFPMMSTFKVLLCGAVLSRVDAGQEQLGRRIHYSQNDLVEYSPVTEKHLTDGMTVRELCSAAITMSDNTAANLLLTTIGGPKELTAFLHNMGDHVTRLDRWEPELNEAIPNDERDTTMPAAMATTLRKLLTGELLTLASRQQLIDWMEADKVAGPLLRSALPAGWFIADKSGAGERGSRGIIAALGPDGKPSRIVVIYTTGSQATMDERNRQIAEIGASLIKHW

**Rieske_TMD_-AmiA**

MSSQDIPEENLPAEQDRPHGAAARPADETNPFADPGLPPHEPRVQDVDERAAKRSERTVALLFTLSMLATIAFIAAFVAIDVDKSVYIFPLGHISALNFALGMTLGVALFAIGAGAVHWARTLMSDEEVADERHPIEASPEVRAKVHADFKQGAKESVIGRRKLIRNTMLGALTLVPLSGVVLLRSRAIAKDELLKTSNGHSKPKAKKSGGKRVVVLDPGHGGIDTGAIGRNGSKEKHVVLAIAKNVRSILRNHGIDARLTRSGDTFIPLYDRVEIAHKHGADLFMSIHADGFTNPKAAGASVFALSNRGASSAMAKYLSERENRADEVAGKKATDKDHLLQQVLFDLVQTDTIKNSLTLGSHILKKIKPVHKLHSRNTEQAAFVVLKSPSVPSVLVETSFITNPEEERLLGTAAFRQKIATAIAEGVISYFHWFDNQKAHSKKR

**MtbRieske_TMD_-Bla (‘short fusion’)**

MSRADDDAVGVPPTCGGRSDEEERRIVPGPNPQDGAKDGAKATAVPREPDEAALAAMSNQELLALGGKLDGVRIAYKEPRWPVEGTKAEKRAERSVAVWLLLGGVFGLALLLIFLFWPWEFKAADGESDFIYSLTTPLYGLTFGLSILSIAIGAVLYQKRFIPEEISIQERHDGASREIDRKTVVANLTDAFEGSTIRRRKLIGLSFGVGMGAFGLGTLVAFAGGLISRHPETLVKVKDAEDQLGARVGYIELDLNSGKILESFRPEERFPMMSTFKVLLCGAVLSRVDAGQEQLGRRIHYSQNDLVEYSPVTEKHLTDGMTVRELCSAAITMSDNTAANLLLTTIGGPKELTAFLHNMGDHVTRLDRWEPELNEAIPNDERDTTMPAAMATTLRKLLTGELLTLASRQQLIDWMEADKVAGPLLRSALPAGWFIADKSGAGERGSRGIIAALGPDGKPSRIVVIYTTGSQATMDERNRQIAEIGASLIKHW

**MtbRieske_TMD_-Bla 243-extended (‘long fusion’)**

MSRADDDAVGVPPTCGGRSDEEERRIVPGPNPQDGAKDGAKATAVPREPDEAALAAMSNQELLALGGKLDGVRIAYKEPRWPVEGTKAEKRAERSVAVWLLLGGVFGLALLLIFLFWPWEFKAADGESDFIYSLTTPLYGLTFGLSILSIAIGAVLYQKRFIPEEISIQERHDGASREIDRKTVVANLTDAFEGSTIRRRKLIGLSFGVGMGAFGLGTLVAFAGGLIKNPWKPVVPTAEGKKASRHPETLVKVKDAEDQLGARVGYIELDLNSGKILESFRPEERFPMMSTFKVLLCGAVLSRVDAGQEQLGRRIHYSQNDLVEYSPVTEKHLTDGMTVRELCSAAITMSDNTAANLLLTTIGGPKELTAFLHNMGDHVTRLDRWEPELNEAIPNDERDTTMPAAMATTLRKLLTGELLTLASRQQLIDWMEADKVAGPLLRSALPAGWFIADKSGAGERGSRGIIAALGPDGKPSRIVVIYTTGSQATMDERNRQIAEIGASLIKHW

**Sco3746_TMD_-AmiA**

MGHWPSGSGAPAYGRSMRDLASRLPSSPGFWRSPLRGPWLTSVLGTVLLVGITVLFVTGLLSYAAYNPDLAPVNDKTPDKGILGFYLFAWPTDPPWLYRLTQGVHVTLGLVLIPVLLAKLWSVVPRLFTLPPVRSLAHALERISLLLLVGGALFEFVTGVLNIQLDYLFPGSFYPLHFYGAWVFFAAFVAHAVLKTPIALRNLRAMREERDDLVSPRPAAPTVSRRGALWFVGGGSLLMFATNAGRSSRAIAKDELLKTSNGHSKPKAKKSGGKRVVVLDPGHGGIDTGAIGRNGSKEKHVVLAIAKNVRSILRNHGIDARLTRSGDTFIPLYDRVEIAHKHGADLFMSIHADGFTNPKAAGASVFALSNRGASSAMAKYLSERENRADEVAGKKATDKDHLLQQVLFDLVQTDTIKNSLTLGSHILKKIKPVHKLHSRNTEQAAFVVLKSPSVPSVLVETSFITNPEEERLLGTAAFRQKIATAIAEGVISYFHWFDNQKAHSKKR

**Sco3746_TMD_-MBP**

MGHWPSGSGAPAYGRSMRDLASRLPSSPGFWRSPLRGPWLTSVLGTVLLVGITVLFVTGLLSYAAYNPDLAPVNDKTPDKGILGFYLFAWPTDPPWLYRLTQGVHVTLGLVLIPVLLAKLWSVVPRLFTLPPVRSLAHALERISLLLLVGGALFEFVTGVLNIQLDYLFPGSFYPLHFYGAWVFFAAFVAHAVLKTPIALRNLRAMREERDDLVSPRPAAPTVSRRGALWFVGGGSLLMFATNAGRSSRKIEEGKLVIWINGDKGYNGLAEVGKKFEKDTGIKVTVEHPDKLEEKFPQVAATGDGPDIIFWAHDRFGGYAQSGLLAEITPDKAFQDKLYPFTWDAVRYNGKLIAYPIAVEALSLIYNKDLLPNPPKTWEEIPALDKELKAKGKSALMFNLQEPYFTWPLIAADGGYAFKYENGKYDIKDVGVDNAGAKAGLTFLVDLIKNKHMNADTDYSIAEAAFNKGETAMTINGPWAWSNIDTSKVNYGVTVLPTFKGQPSKPFVGVLSAGINAASPNKELAKEFLENYLLTDEGLEAVNKDKPLGAVALKSYEEELAKDPRIAATMENAQKGEIMPNIPQMSAFWYAVRTAVINAASGRQTVDEALKDAQTRITK

**Sco3746_TMD_-Bla (‘short fusion’)**

MGHWPSGSGAPAYGRSMRDLASRLPSSPGFWRSPLRGPWLTSVLGTVLLVGITVLFVTGLLSYAAYNPDLAPVNDKTPDKGILGFYLFAWPTDPPWLYRLTQGVHVTLGLVLIPVLLAKLWSVVPRLFTLPPVRSLAHALERISLLLLVGGALFEFVTGVLNIQLDYLFPGSFYPLHFYGAWVFFAAFVAHAVLKTPIALRNLRAMREERDDLVSPRPAAPTVSRRGALWFVGGGSLLMFATNAGRSFDSPLGTHPETLVKVKDAEDQLGARVGYIELDLNSGKILESFRPEERFPMMSTFKVLLCGAVLSRVDAGQEQLGRRIHYSQNDLVEYSPVTEKHLTDGMTVRELCSAAITMSDNTAANLLLTTIGGPKELTAFLHNMGDHVTRLDRWEPELNEAIPNDERDTTMPAAMATTLRKLLTGELLTLASRQQLIDWMEADKVAGPLLRSALPAGWFIADKSGAGERGSRGIIAALGPDGKPSRIVVIYTTGSQATMDERNRQIAEIGASLIKHW

**Sco3746_TMD_-Bla extended (‘long fusion’)**

MGHWPSGSGAPAYGRSMRDLASRLPSSPGFWRSPLRGPWLTSVLGTVLLVGITVLFVTGLLSYAAYNPDLAPVNDKTPDKGILGFYLFAWPTDPPWLYRLTQGVHVTLGLVLIPVLLAKLWSVVPRLFTLPPVRSLAHALERISLLLLVGGALFEFVTGVLNIQLDYLFPGSFYPLHFYGAWVFFAAFVAHAVLKTPIALRNLRAMREERDDLVSPRPAAPTVSRRGALWFVGGGSLLMFATNAGRSFDSPLRATAVLSPHGGPEPGGGPNGGTHPETLVKVKDAEDQLGARVGYIELDLNSGKILESFRPEERFPMMSTFKVLLCGAVLSRVDAGQEQLGRRIHYSQNDLVEYSPVTEKHLTDGMTVRELCSAAITMSDNTAANLLLTTIGGPKELTAFLHNMGDHVTRLDRWEPELNEAIPNDERDTTMPAAMATTLRKLLTGELLTLASRQQLIDWMEADKVAGPLLRSALPAGWFIADKSGAGERGSRGIIAALGPDGKPSRIVVIYTTGSQATMDERNRQIAEIGASLIKHW

**PFD_TMD_-AmiA**

MNFSRLKSLRGWRLLSQWFFLLLFLFLFIETEGKGGDELGWPVKLFLDFNPLILLTTLLSAHTVPAIFLLALVVVALTLLLGRVFCGWICPFGTIHNLLSLASNRSRRLVGHPAWLRLKYLILIMMLAMAALGIQQAGLLDPISLLIRSLAVGIYPAFSYAISAFFDTIYLWQIGGVSAVAEWFYGILRDTVLPFQQPIFSQAGLIGGLFIAILALNFYERRFWCRYLCPLGALLGLLARWSLLSREVAEGCNHCGACGQHCPGGAEPHALPDFAGQHQTGAHQPTASPPWLRSECHACFNCDDICPQRLISFRWRLPGLGGKPRSAVSAPAVAGPDLGRRRLLGAAAAGLVVGPLLRVSNPPESRAIAKDELLKTSNGHSKPKAKKSGGKRVVVLDPGHGGIDTGAIGRNGSKEKHVVLAIAKNVRSILRNHGIDARLTRSGDTFIPLYDRVEIAHKHGADLFMSIHADGFTNPKAAGASVFALSNRGASSAMAKYLSERENRADEVAGKKATDKDHLLQQVLFDLVQTDTIKNSLTLGSHILKKIKPVHKLHSRNTEQAAFVVLKSPSVPSVLVETSFITNPEEERLLGTAAFRQKIATAIAEGVISYFHWFDNQKAHSKKR

**PFD_TMD_-MBP**

MNFSRLKSLRGWRLLSQWFFLLLFLFLFIETEGKGGDELGWPVKLFLDFNPLILLTTLLSAHTVPAIFLLALVVVALTLLLGRVFCGWICPFGTIHNLLSLASNRSRRLVGHPAWLRLKYLILIMMLAMAALGIQQAGLLDPISLLIRSLAVGIYPAFSYAISAFFDTIYLWQIGGVSAVAEWFYGILRDTVLPFQQPIFSQAGLIGGLFIAILALNFYERRFWCRYLCPLGALLGLLARWSLLSREVAEGCNHCGACGQHCPGGAEPHALPDFAGQHQTGAHQPTASPPWLRSECHACFNCDDICPQRLISFRWRLPGLGGKPRSAVSAPAVAGPDLGRRRLLGAAAAGLVVGPLLRVSNPPESRKIEEGKLVIWINGDKGYNGLAEVGKKFEKDTGIKVTVEHPDKLEEKFPQVAATGDGPDIIFWAHDRFGGYAQSGLLAEITPDKAFQDKLYPFTWDAVRYNGKLIAYPIAVEALSLIYNKDLLPNPPKTWEEIPALDKELKAKGKSALMFNLQEPYFTWPLIAADGGYAFKYENGKYDIKDVGVDNAGAKAGLTFLVDLIKNKHMNADTDYSIAEAAFNKGETAMTINGPWAWSNIDTSKVNYGVTVLPTFKGQPSKPFVGVLSAGINAASPNKELAKEFLENYLLTDEGLEAVNKDKPLGAVALKSYEEELAKDPRIAATMENAQKGEIMPNIPQMSAFWYAVRTAVINAASGRQTVDEALKDAQTRITK

**PFD_TMD_-Bla (‘short fusion’)**

MNFSRLKSLRGWRLLSQWFFLLLFLFLFIETEGKGGDELGWPVKLFLDFNPLILLTTLLSAHTVPAIFLLALVVVALTLLLGRVFCGWICPFGTIHNLLSLASNRSRRLVGHPAWLRLKYLILIMMLAMAALGIQQAGLLDPISLLIRSLAVGIYPAFSYAISAFFDTIYLWQIGGVSAVAEWFYGILRDTVLPFQQPIFSQAGLIGGLFIAILALNFYERRFWCRYLCPLGALLGLLARWSLLSREVAEGCNHCGACGQHCPGGAEPHALPDFAGQHQTGAHQPTASPPWLRSECHACFNCDDICPQRLISFRWRLPGLGGKPRSAVSAPAVAGPDLGRRRLLGAAAAGLVVGPLLRVSNPPEGRANPLLGTHPETLVKVKDAEDQLGARVGYIELDLNSGKILESFRPEERFPMMSTFKVLLCGAVLSRVDAGQEQLGRRIHYSQNDLVEYSPVTEKHLTDGMTVRELCSAAITMSDNTAANLLLTTIGGPKELTAFLHNMGDHVTRLDRWEPELNEAIPNDERDTTMPAAMATTLRKLLTGELLTLASRQQLIDWMEADKVAGPLLRSALPAGWFIADKSGAGERGSRGIIAALGPDGKPSRIVVIYTTGSQATMDERNRQIAEIGASLIKHW

**PFD_TMD_-Bla extended (‘long fusion’)**

MNFSRLKSLRGWRLLSQWFFLLLFLFLFIETEGKGGDELGWPVKLFLDFNPLILLTTLLSAHTVPAIFLLALVVVALTLLLGRVFCGWICPFGTIHNLLSLASNRSRRLVGHPAWLRLKYLILIMMLAMAALGIQQAGLLDPISLLIRSLAVGIYPAFSYAISAFFDTIYLWQIGGVSAVAEWFYGILRDTVLPFQQPIFSQAGLIGGLFIAILALNFYERRFWCRYLCPLGALLGLLARWSLLSREVAEGCNHCGACGQHCPGGAEPHALPDFAGQHQTGAHQPTASPPWLRSECHACFNCDDICPQRLISFRWRLPGLGGKPRSAVSAPAVAGPDLGRRRLLGAAAAGLVVGPLLRVSNPPEGRANPLLIRPGTHPETLVKVKDAEDQLGARVGYIELDLNSGKILESFRPEERFPMMSTFKVLLCGAVLSRVDAGQEQLGRRIHYSQNDLVEYSPVTEKHLTDGMTVRELCSAAITMSDNTAANLLLTTIGGPKELTAFLHNMGDHVTRLDRWEPELNEAIPNDERDTTMPAAMATTLRKLLTGELLTLASRQQLIDWMEADKVAGPLLRSALPAGWFIADKSGAGERGSRGIIAALGPDGKPSRIVVIYTTGSQATMDERNRQIAEIGASLIKHW

**Supplementary file 1C. Plasmids used in this work.**

| Plasmid | Relevant features | Reference |
| --- | --- | --- |
| pSU-PROM | Expression vector with constitutive *E. coli* *tatA* promoter; Kan^R^ | (28) |
| pSU-PROM Sco2149_TMD_‑AmiA | pSU-PROM producing aa 1-185 of Sco2149 fused to aa 32-289 of *E. coli* AmiA | This work |
| pSU-PROM Sco2149_TMD_-Bla | pSU-PROM producing aa 1-185 of Sco2149 fused to aa 24-286 of β‑lactamase (from pBR322) | This work |
| Constructs based on pSU-PROM Sco2149_TMD_-Bla | | |
| Sco2149_TMD_ RHHR-Bla | Substitution of aa 133-134 of Sco2149_TMD_ from RH to HR | This work |
| Sco2149_TMD_ RHKK-Bla | Substitution of aa 133-134 of Sco2149_TMD_ from RH to KK | This work |
| Sco3149_TMD_ A144 -Bla | Substitution of aa 144 of Sco2149_TMD_ from A to P | This work |
| Sco3149_TMD_ A148P-Bla | Substitution of aa 148 of Sco2149_TMD_ from A to P | This work |
| Sco3149_TMD_ A154P-Bla | Substitution of aa 154 of Sco2149_TMD_ from A to P | This work |
| Sco3149_TMD_ M124A-Bla | Substitution of aa 124 of Sco2149_TMD_ from M to A | This work |
| Sco2149_TMD_ S125A-Bla | Substitution of aa 125 of Sco2149_TMD_ from S to A | This work |
| Sco2149_TMD_ D126A-Bla | Substitution of aa 126 of Sco2149_TMD_ from D to A | This work |
| Sco2149_TMD_ E127A-Bla | Substitution of aa 127 of Sco2149_TMD_ from E to A | This work |
| Sco2149_TMD_ M124L-Bla | Substitution of aa 124 of Sco2149_TMD_ from M to L | This work |
| Sco2149_TMD_ S125L-Bla | Substitution of aa 125 of Sco2149_TMD_ from S to L | This work |
| Sco2149_TMD_ D126L-Bla | Substitution of aa 126 of Sco2149_TMD_ from D to L | This work |
| Sco2149_TMD_ E127L-Bla | Substitution of aa 127 of Sco2149_TMD_ from E to L | This work |
| Sco2149_TMD_ RRKK-Bla | Substitution of aa 161-162 of Sco2149_TMD_ from RR to KK | This work |
| Sco2149_TMD_ RRKQ-Bla | Substitution of aa 161-162 of Sco2149_TMD_ from RR to KQ | This work |
| Sco2149_TMD_ RRAA-Bla | Substitution of aa 161-162 of Sco2149_TMD_ from RR to AA | This work |
| Sco2149_TMD_ RRAD-Bla | Substitution of aa 161-162 of Sco2149_TMD_ from RR to AD | This work |
| Sco2149_TMD_∆RR -Bla | Deletion of aa 161-162 of Sco2149_TMD_ | This work |
| Sco2149_TMD_∆118-122-Bla | Deletion of aa 118-122 of Sco2149_TMD_ | This work |
| Sco2149_TMD_∆123-127-Bla | Deletion of aa 123-127 of Sco2149_TMD_ | This work |
| Sco2149_TMD_∆128-132-Bla | Deletion of aa 128-132 of Sco2149_TMD_ | This work |
| Sco2149_TMD_∆133-137-Bla | Deletion of aa 133-137 of Sco2149_TMD_ | This work |
| Sco2149_TMD_∆138-142-Bla | Deletion of aa 138-142 of Sco2149_TMD_ | This work |
| Sco2149_TMD_∆143-147-Bla | Deletion of aa 143-147 of Sco2149_TMD_ | This work |
| Sco2149_TMD_∆148-152-Bla | Deletion of aa 148-152 of Sco2149_TMD_ | This work |
| Sco2149_TMD_∆153-157-Bla | Deletion of aa 153-157 of Sco2149_TMD_ | This work |
| Sco2149_TMD_∆118-127-Bla | Deletion of aa 118-127 of Sco2149_TMD_ | This work |
| Sco2149_TMD_∆128-137-Bla | Deletion of aa 128-137 of Sco2149_TMD_ | This work |
| Sco2149_TMD_∆138-147-Bla | Deletion of aa 138-147 of Sco2149_TMD_ | This work |
| Sco2149_TMD_∆148-157-Bla | Deletion of aa 148-157 of Sco2149_TMD_ | This work |
| Sco2149_TMD_∆138-152-Bla | Deletion of aa 138-152 of Sco2149_TMD_ | This work |
| Sco2149_TMD_∆118-132-Bla | Deletion of aa 118-132 of Sco2149_TMD_ | This work |
| Sco2149_TMD_∆123-137-Bla | Deletion of aa 123-137 of Sco2149_TMD_ | This work |
| Sco2149_TMD_∆128-142-Bla | Deletion of aa 128-142 of Sco2149_TMD_ | This work |
| Sco2149_TMD_∆133-147-Bla | Deletion of aa 133-147 of Sco2149_TMD_ | This work |
| Sco2149_TMD_∆143-157-Bla | Deletion of aa 143-157 of Sco2149_TMD_ | This work |
| Sco2149_TMD_∆118-137-Bla | Deletion of aa 118-137 of Sco2149_TMD_ | This work |
| Sco2149_TMD_∆138-157-Bla | Deletion of aa 138-157 of Sco2149_TMD_ | This work |
| Sco2149_TMD_∆118-142-Bla | Deletion of aa 118-142 of Sco2149_TMD_ | This work |
| Sco2149_TMD_∆123-147-Bla | Deletion of aa 123-147 of Sco2149_TMD_ | This work |
| Sco2149_TMD_∆128-152-Bla | Deletion of aa 128-152 of Sco2149_TMD_ | This work |
| Sco2149_TMD_∆133-157-Bla | Deletion of aa 133-157 of Sco2149_TMD_ | This work |
| Sco2149_TMD_∆118-147-Bla | Deletion of aa 118-147 of Sco2149_TMD_ | This work |
| Sco2149_TMD_∆123-152-Bla | Deletion of aa 123-152 of Sco2149_TMD_ | This work |
| Sco2149_TMD_∆128-157-Bla | Deletion of aa 128-157 of Sco2149_TMD_ | This work |
| Sco2149_TMD_∆123-157-Bla | Deletion of aa 123-157 of Sco2149_TMD_ | This work |
| Sco2149_TMD_∆118-152-Bla | Deletion of aa 118-152 of Sco2149_TMD_ | This work |
| Sco2149_TMD_∆118-153-Bla | Deletion of aa 118-153 of Sco2149_TMD_ | This work |
| Sco2149_TMD_∆118-154-Bla | Deletion of aa 118-154 of Sco2149_TMD_ | This work |
| Sco2149_TMD_∆118-155-Bla | Deletion of aa 118-155 of Sco2149_TMD_ | This work |
| Sco2149_TMD_∆118-156-Bla | Deletion of aa 118-156 of Sco2149_TMD_ | This work |
| Sco2149_TMD_∆118-157-Bla | Deletion of aa 118-157 of Sco2149_TMD_ | This work |
| Sco2149_TMD_∆126-127-Bla | Deletion of aa 126-127of Sco2149_TMD_ | This work |
| Sco2149_TMD_∆127-128-Bla | Deletion of aa 127-128 of Sco2149_TMD_ | This work |
| Sco2149_TMD_∆126-128-Bla | Deletion of aa 126-128 of Sco2149_TMD_ | This work |
| Sco2149_TMD_∆131-132-Bla | Deletion of aa 131-132 of Sco2149_TMD_ | This work |
| Sco2149_TMD_∆137Δ141-Bla | Deletion of aa 137 & 141of Sco2149_TMD_ | This work |
| Sco2149_TMD_∆149Δ156-Bla | Deletion of aa 149 & 156 of Sco2149_TMD_ | This work |
| Sco2149_TMD_∆131-132 Δ141-Bla | Deletion of aa 131 & 132 & 141 of Sco2149_TMD_ | This work |
| Sco2149_TMD_∆126-128 Δ131-132-Bla | Deletion of aa 126-128 &131-132 of Sco2149_TMD_ | This work |
| Sco2149_TMD_∆126-128 Δ137 Δ141-Bla | Deletion of aa 126-128 &137 &141 of Sco2149_TMD_ | This work |
| Sco2149_TMD_∆131-132 Δ137 Δ141-Bla | Deletion of aa 131-132 & 137 &141 of Sco2149_TMD_ | This work |
| Sco2149_TMD_-Bla ∆126-128 Δ131-132 Δ137 Δ141 | Deletion of aa 126-128 &131-132 &137 & 141 of Sco2149_TMD_ | This work |
| Sco2149_TMD_-Bla ∆126-128 Δ131-132 Δ137 Δ141 Δ149 Δ156 | Deletion of aa 126-128 &131-132 &137 & 141 &149 & 156 of Sco2149_TMD_ | This work |
| Sco2149_TMD_ D131A E132A-Bla | Substitution of aa 131 & 132 of Sco2149_TMD_ to A | This work |
| Sco2149_TMD_ E137A E141A-Bla | Substitution of aa 137 & 141 of Sco2149_TMD_ to A | This work |
| Sco2149_TMD_ D126A E127A E128A -Bla | Substitution of aa 126 & 127 & 128 of Sco2149_TMD_ to A | This work |
| Sco2149_TMD_ D131K E132K-Bla | Substitution of aa 131 & 132 of Sco2149_TMD_ to K | This work |
| Sco2149_TMD_ E137K E141K-Bla | Substitution of aa 137 & 141 of Sco2149_TMD_ to K | This work |
| Sco2149_TMD_ D126K E127K E128K-Bla | Substitution of aa 126 & 127 & 128 of Sco2149_TMD_ to K | This work |
| Sco2149_TMD_D129 E130 E131-Bla INS | Insertion of DEE at positions aa 129-131 of Sco2149_TMD_ | This work |
| Sco2149_TMD_D126K E127K E128K E137K E141K-Bla | Substitution of aa 126-128 & 137 & 141 of Sco2149_TMD_ to K | This work |
| Sco2149_TMD_ Sco2149_TMD_D126K E127K E128K D131K E132K E137K E141K -Bla | Substitution of aa 126-128 & 131-132 & 137 & 141 of Sco2149_TMD_ to K | This work |
| Sco2149_TMD_P177L S179L G180L-Bla | Substitution of aa 177 &179 & 180 of Sco2149_TMD_ to L | This work |
| Sco2149_TMD_S179L G180L-Bla | Substitution of aa 179 & 180 of Sco2149_TMD_ to L | This work |
| Sco2149_TMD_G180L-Bla | Substitution of aa 180 of Sco2149_TMD_ to L | This work |
| Sco2149_TMD_S179L-Bla | Substitution of aa 179 of Sco2149_TMD_ to L | This work |
| Sco2149_TMD_R185A-Bla | Substitution of aa 185 of Sco2149_TMD_ to A | This work |
| Sco2149_TMD_V158K-Bla | Substitution of aa 158 of Sco2149_TMD_ to K | This work |
| Sco2149_TMD_∆118-155 V158K -Bla | Deletion of aa 118-155 of Sco2149_TMD_ & substitution of aa 158 of Sco2149_TMD_ to K | This work |
| Sco2149_TMD_∆118-156 V158K -Bla | Deletion of aa 118-156 of Sco2149_TMD_ & substitution of aa 158 of Sco2149_TMD_ to K | This work |
| Sco2149_TMD_∆118-157 V158K -Bla | Deletion of aa 118-157 of Sco2149_TMD_ & substitution of aa 158 of Sco2149_TMD_ to K | This work |
| pSU-PROM Sco2149_TMD_extended-Bla | pSU-PROM Sco2149_TMD_-Bla extension of sequence from aa 185 to 205 | This work |
| Constructs based on pSU-PROM Sco2149_TMD_-AmiA | | |
| Sco2149_TMD_ RHHR-AmiA | Substitution of aa 133-134 of Sco2149_TMD_ from RH to HR | This work |
| Sco2149_TMD_ RHKK-AmiA | Substitution of aa 133-134 of Sco2149_TMD_ from RH to KK | This work |
| Sco2149_TMD_ A144P-AmiA | Substitution of aa 144 of Sco2149_TMD_ from A to P | This work |
| Sco2149_TMD_ A148P-AmiA | Substitution of aa 148 of Sco2149_TMD_ from A to P | This work |
| Sco2149_TMD_ A154P-AmiA | Substitution of aa 154 of Sco2149_TMD_ from A to P | This work |
| Sco2149_TMD_ M124A-AmiA | Substitution of aa 124 of Sco2149_TMD_ from M to A | This work |
| Sco2149_TMD_ S125A-AmiA | Substitution of aa 125 of Sco2149_TMD_ from S to A | This work |
| Sco2149_TMD_ D126A-AmiA | Substitution of aa 126 of Sco2149_TMD_ from D to A | This work |
| Sco2149_TMD_ E127A-AmiA | Substitution of aa 127 of Sco2149_TMD_ from E to A | This work |
| Sco2149_TMD_ M124L-AmiA | Substitution of aa 124 of Sco2149_TMD_ from M to L | This work |
| Sco2149_TMD_ S125L-AmiA | Substitution of aa 125 of Sco2149_TMD_ from S to L | This work |
| Sco2149_TMD_ D126L-AmiA | Substitution of aa 126 of Sco2149_TMD_ from D to L | This work |
| Sco2149_TMD_ E127L-AmiA | Substitution of aa 127 of Sco2149_TMD_ from E to L | This work |
| Sco2149_TMD_ RRKK-AmiA | Substitution of aa 161-162 of Sco2149_TMD_ from RR to KK | This work |
| Sco2149_TMD_ RRKQ-AmiA | Substitution of aa 161-162 of Sco2149_TMD_ from RR to KQ | This work |
| Sco2149_TMD_ RRAA-AmiA | Substitution of aa 161-162 of Sco2149_TMD_ from RR to AA | This work |
| Sco2149_TMD_ RRAD-AmiA | Substitution of aa 161-162 of Sco2149_TMD_ from RR to AD | This work |
| Sco2149_TMD_ ∆RR -AmiA | Deletion of aa 161-162 of Sco2149_TMD_ | This work |
| Sco2149_TMD_∆118-122-AmiA | Deletion of aa 118-122 of Sco2149_TMD_ | This work |
| Sco2149_TMD_∆123-127-AmiA | Deletion of aa 123-127 of Sco2149_TMD_ | This work |
| Sco2149_TMD_∆128-132-AmiA | Deletion of aa 128-132 of Sco2149_TMD_ | This work |
| Sco2149_TMD_∆133-137-AmiA | Deletion of aa 133-137 of Sco2149_TMD_ | This work |
| Sco2149_TMD_∆138-142-AmiA | Deletion of aa 138-142 of Sco2149_TMD_ | This work |
| Sco2149_TMD_∆143-147-AmiA | Deletion of aa 143-147 of Sco2149_TMD_ | This work |
| Sco2149_TMD_∆148-152-AmiA | Deletion of aa 148-152 of Sco2149_TMD_ | This work |
| Sco2149_TMD_∆153-157-AmiA | Deletion of aa 153-157 of Sco2149_TMD_ | This work |
| Sco2149_TMD_∆118-127-AmiA | Deletion of aa 118-127 of Sco2149_TMD_ | This work |
| Sco2149_TMD_∆128-137-AmiA | Deletion of aa 128-137 of Sco2149_TMD_ | This work |
| Sco2149_TMD_∆138-147-AmiA | Deletion of aa 138-147 of Sco2149_TMD_ | This work |
| Sco2149_TMD_∆148-157-AmiA | Deletion of aa 148-157 of Sco2149_TMD_ | This work |
| Sco2149_TMD_∆138-152-AmiA | Deletion of aa 138-152 of Sco2149_TMD_ | This work |
| Sco2149_TMD_∆118-132-AmiA | Deletion of aa 118-132 of Sco2149_TMD_ | This work |
| Sco2149_TMD_-∆123-137AmiA | Deletion of aa 123-137 of Sco2149_TMD_ | This work |
| Sco2149_TMD_∆128-142-AmiA | Deletion of aa 128-142 of Sco2149_TMD_ | This work |
| Sco2149_TMD_∆133-147-AmiA | Deletion of aa 133-147 of Sco2149_TMD_ | This work |
| Sco2149_TMD_∆143-157-AmiA | Deletion of aa 143-157 of Sco2149_TMD_ | This work |
| Sco2149_TMD_∆118-137-AmiA | Deletion of aa 118-137 of Sco2149_TMD_ | This work |
| Sco2149_TMD_∆138-157-AmiA | Deletion of aa 138-157 of Sco2149_TMD_ | This work |
| Sco2149_TMD_∆118-142-AmiA | Deletion of aa 118-142 of Sco2149_TMD_ | This work |
| Sco2149_TMD_∆123-147-AmiA | Deletion of aa 123-147 of Sco2149_TMD_ | This work |
| Sco2149_TMD_∆128-152-AmiA | Deletion of aa 128-152 of Sco2149_TMD_ | This work |
| Sco2149_TMD_∆133-157-AmiA | Deletion of aa 133-157 of Sco2149_TMD_ | This work |
| Sco2149_TMD_∆118-147-AmiA | Deletion of aa 118-147 of Sco2149_TMD_ | This work |
| Sco2149_TMD_∆123-152-AmiA | Deletion of aa 123-152 of Sco2149_TMD_ | This work |
| Sco2149_TMD_-∆128-157AmiA | Deletion of aa 128-157 of Sco2149_TMD_ | This work |
| Sco2149_TMD_∆123-157-AmiA | Deletion of aa 123-157 of Sco2149_TMD_ | This work |
| Sco2149_TMD_∆118-152-AmiA | Deletion of aa 118-152 of Sco2149_TMD_ | This work |
| Sco2149_TMD_∆118-153-AmiA | Deletion of aa 118-153 of Sco2149_TMD_ | This work |
| Sco2149_TMD_∆118-154-AmiA | Deletion of aa 118-154 of Sco2149_TMD_ | This work |
| Sco2149_TMD_∆118-155-AmiA | Deletion of aa 118-155 of Sco2149_TMD_ | This work |
| Sco2149_TMD_∆118-156-AmiA | Deletion of aa 118-156 of Sco2149_TMD_ | This work |
| Sco2149_TMD_∆118-157-AmiA | Deletion of aa 118-157 of Sco2149_TMD_ | This work |
| Sco2149_TMD_∆124-157-AmiA | Deletion of aa 118-155 of Sco2149_TMD_ | This work |
| Sco2149_TMD_∆125-157-AmiA | Deletion of aa 118-156 of Sco2149_TMD_ | This work |
| pSU18 | Expression vector with a constitutive *E. coli* *lac* promoter. Cml^R^ | (59) |
| pSU18 AmiA | pSU18 producing aa 1-289 of *E. coli* AmiA | (26) |
| pSU18 Sco2149_TMD_-AmiA (pSU-TM123-AmiA) | pSU18 producing aa 1-185 of Sco2149 fused to aa 32-289 of *E. coli* AmiA | (22) |
| pSU-PROM MtbRieske_TMD_-Bla | pSU-PROM producing aa 1-227 of *M. tuberculosis* H37Rv *qcrA* codon optimised for *E. coli* fused to aa 24-286 of β‑lactamase (from pBR322) | This work |
| pSU-PROM MtbRieske_TMD_extended -Bla | pSU-PROM MtbRieke_TMD_-Bla extension of sequence from aa 227 to 243 | This work |
| pSU18-PROM Sco3746_TMD_-AmiA | pSU18 producing aa 1-247 of Sco3746 fused via an Ala-Ile-Ala linker to aa 32-289 of *E. coli* AmiA under control of constitutive *E. coli* *tat* promoter | This work |
| pSU18-PROM Sco3746_TMD_-RRKK-AmiA | pSU18-PROM Sco3746_TMD_-AmiA with substitution of aa 225-226 of Sco3746 from RR to KK | This work |
| pSU18-PROM Sco3746_TMD_-MBP | pSU18 producing aa 1-247 of Sco3746 fused to aa 29-396 of *E. coli* MBP under control of constitutive *E. coli* *tat* promoter | This work |
| pSU18-PROM Sco3746_TMD_ RRKK-MBP | pSU18-PROM Sco3746_TMD_-MBP with substitution of aa 225-226 of Sco3746 from RR to KK | This work |
| pSU1-8PROM Sco3746_TMD_ G14C-MBP | pSU18-PROM Sco3746_TMD_-MBP with substitution of aa G14 of Sco3746 to C | This work |
| pSU18-PROM Sco3746_TMD_ G84C-MBP | pSU18-PROM Sco3746_TMD_-MBP with substitution of aa G84 of Sco3746 to C | This work |
| pSU18-PROM Sco3746_TMD_ A137C-MBP | pSU18-PROM Sco3746_TMD_-MBP with substitution of aa A137 of Sco3746 to C | This work |
| pSU1-8PROM Sco3746_TMD_ G171C-MBP | pSU18-PROM Sco3746_TMD_-MBP with substitution of aa G171 of Sco3746 to C | This work |
| pSU18-PROM Sco3746_TMD_ A219C-MBP | pSU18-PROM Sco3746_TMD_-MBP with substitution of aa A219 of Sco3746 to C | This work |
| pSU18-PROM Sco3746_TMD_-Bla | pSU18 producing aa 1-252 of Sco3746 fused to aa 24-286 of β‑lactamase (from pBR322) under control of constitutive *E. coli* *tat* promoter. | This work |
| pSU18-PROM Sco3746_TMD_extended-Bla | As pSU18PROM Sco3746_TMD_-Bla but with extension of Sco3746_TMD_ sequence from aa 252 to 272 | This work |
| pSU18PROM Sco3746_TMD_ G234L S235L-Bla | pSU18PROM Sco3746_TMD_-Bla with substitution of aa 234-235 of Sco3746 to L | This work |
| pSU18PROM Sco3746_TMD_ G234L S235L M239L F240L-Bla | pSU18PROM Sco3746_TMD_-Bla with substitution of aa 234-235 and 239-240 of Sco3746 to L | This work |
| pSU18 PFD_TMD_-AmiA | pSU18 producing aa 1-364 of PFD fused via an Ala-Ile-Ala linker to aa 32-289 of *E. coli* AmiA | This work |
| pSU18 PFD_TMD_ RRKK-AmiA | pSU18 PFD_TMD_-AmiA with substitution of aa 340-341 of PFD from RR to KK | This work |
| pSU18 PFD_TMD_-MBP | pSU18 producing aa 1-364 of PFD fused to aa 29-396 of *E. coli* MBP | This work |
| pSU18 PFD_TMD_ RRKK-MBP | pSU18 PFD_TMD_-MBP with substitution of aa 340-341 of PFD from RR to KK | This work |
| pSU18 PFD_TMD_-Bla | pSU18 producing aa 1-371 of PFD fused to aa 24-286 of β‑lactamase (from pBR322) under control of constitutive *E. coli* *tat* promoter. | This work |
| pSU18 PFD_TMD_extended-Bla | As pSU18 PFD_TMD_-Bla but with extension of PFD_TMD_ sequence from aa 371 to 374 | This work |
| pSU18 PFD_TMD_ G354L R358L-Bla | pSU18 PFD_TMD_-Bla with substitution of aa 354 and-358 of PFD to L | This work |
| pSU18 PFD_TMD_ G354L P355L R358L-Bla | pSU18 PFD_TMD_-Bla with substitution of aa 354-355 and-358 of PFD to L | This work |
| pSU18 PFD_TMD_extended G354L R358L-Bla | pSU18 PFD_TMD_extended-Bla with substitution of aa 354 and-358 of PFD to L | This work |
| pSU18 PFD_TMD_extended G354L P355L R358L-Bla | pSU18 PFD_TMD_extended-Bla with substitution of aa 354-355 and-358 of PFD to L | This work |

**Supplementary file 1D. Oligonucleotides used in this work.**

| Primer | Sequence | Use |
| --- | --- | --- |
| BamHI AmiA | GCGCGGATCCATGAGCACTTTTAAACCACTAAAA | Construction of AmiA fusions |
| SU18.1 | CGTATGTTGTGTGGAATTGTGAGC | pSU18/pSU40 sequencing primer |
| SU18.2 | GGGTAACGCCAGGGTTTTCCC | pSU18/pSU40 sequencing primer |
| Uni-Rep1-Hind | GCGCAAGCTTTGTCGGTTGGCGCAAAACACGCTG | Amplification of *tat* promoter |
| Inside Bla reverse | GCTGCAGGCATCGTGGTGTCACGCTCGTC | *bla* sequencing primer |
| Inside AmiA reverse | TCAAACAGCACTTGTTGCAATAGGTGATC | *amiA* sequencing primer |
| MalE internal reverse | CCACGTCTTTAATGTCGTACTT | *malE* (MBP) sequencing primer |
| Sco2149_TMD_ | GCGCGGATCCATGAGTAGCCAAGACATT | Construction of Sco2149_TMD_ extended |
| Sco2149_TMD_ extension | GCGCTCTAGAGAGCTTGCCCTTGGACCACAGGGTGTGGCGGAGCTTGGTCCCGGGCAGCGGACCGAGGTCGCGCAGCAGGACGACGCC | Construction of Sco2149_TMD_ extended |
| MtbRieske_TMD_ | GCGCGGATCCATGAGCCGCGCGGATGAT | Construction of MtbRieske_TMD_ extended |
| MtbRieske_TMD_ extension | GCGCTCTAGACGCCTTCTTGCCTTCCGCGGTCGGCACCACCGGTTTCCACGGGTTTTTAATCAGGCCGCCCGCAAA | Construction of MtbRieske_TMD_ extended |
| Sco2149_TMD_ RHHR | GTCGCCGACGAGCATCGCCCGATCGAGGCG | Sco2149_TMD_ Quikchange |
|  | CGCCTCGATCGGGCGATGCTCGTCGGCGAC |  |
| Sco2149_TMD_ RHKK | GTCGCCGACGAGAAAAAACCGATCGAGGCG | Sco2149_TMD_ Quikchange |
|  | CGCCTCGATCGGTTTTTTCTCGTCGGCGAC |  |
| Sco2149_TMD_ A144P | TCCCCCGAGGTCCGTCCCAAGGTCCACGCGGAC | Sco2149_TMD_ Quikchange |
|  | GTCCGCGTGGACCTTGGGACGGACCTCGGGGGA |  |
| Sco2149_TMD_ A148P | CGTGCCAAGGTCCACCCGGACTTCAAGCAGGGT | Sco2149_TMD_ Quikchange |
|  | ACCCTGCTTGAAGTCCGGGTGGACCTTGGCACG |  |
| Sco2149_TMD_ A154P | GACTTCAAGCAGGGTCCCAAGGAGTCCGTGATC | Sco2149_TMD_ Quikchange |
|  | CAGCACGGACTCCTTGGGACCCTGCTTGAAGTC |  |
| Sco2149_TMD_ M124A | TGGGCCCGCACCCTGGCCTCCGACGAGGAGGTC | Sco2149_TMD_ Quikchange |
|  | GACCTCCTCGTCGGAGGCCAGGGTGCGGGCCCA |  |
| Sco2149_TMD_ S125A | GCCCGCACCCTGATGGCCGACGAGGAGGTCGCC | Sco2149_TMD_ Quikchange |
|  | GGCGACCTCCTCGTCGGCCATCAGGGTGCGGGC |  |
| Sco2149_TMD_ D126A | CGCACCCTGATGTCCGCCGAGGAGGTCGCCGAC | Sco2149_TMD_ Quikchange |
|  | GTCGGCGACCTCCTCGGCGGACATCAGGGTGCG |  |
| Sco2149_TMD_ E127A | ACCCTGATGTCCGACGCCGAGGTCGCCGACGAG | Sco2149_TMD_ Quikchange |
|  | CTCGTCGGCGACCTCGGCGTCGGACATCAGGGT |  |
| Sco2149_TMD_ M124L | TGGGCCCGCACCCTGTTGTCCGACGAGGAGGTC | Sco2149_TMD_ Quikchange |
|  | GACCTCCTCGTCGGACAACAGGGTGCGGGCCCA |  |
| Sco2149_TMD_ S125L | GCCCGCACCCTGATGTTGGACGAGGAGGTCGCC | Sco2149_TMD_ Quikchange |
|  | GGCGACCTCCTCGTCCAACATCAGGGTGCGGGC |  |
| Sco2149_TMD_ D126L | CGCACCCTGATGTCCTTGGAGGAGGTCGCCGAC | Sco2149_TMD_ Quikchange |
|  | GTCGGCGACCTCCTCCAAGGACATCAGGGTGCG |  |
| Sco2149_TMD_ E127L | ACCCTGATGTCCGACTTGGAGGTCGCCGACGAG | Sco2149_TMD_ Quikchange |
|  | CTCGTCGGCGACCTCCAAGTCGGACATCAGGGT |  |
| Sco2149_TMD_ RRKK | GCCAAGGAGTCCGTGATCGGGAAGAAGAAGCTGATCCGCAACACG | Sco2149_TMD_ Quikchange |
|  | CGTGTTGCGGATCAGCTTCTTCTTCCCGATCACGGACTCCTTGGC |  |
| Sco2149_TMD_ RRKQ | GCCAAGGAGTCCGTGATCGGGAAGCAGAAGCTGATCCGCAACACG | Sco2149_TMD_ Quikchange |
|  | CGTGTTGCGGATCAGCTTCTGCTTCCCGATCACGGACTCCTTGGC |  |
| Sco2149_TMD_ RRAA | GCCAAGGAGTCCGTGATCGGGGCCGCCAAGCTGATCCGCAACACG | Sco2149_TMD_ Quikchange |
|  | CGTGTTGCGGATCAGCTTGGCGGCCCCGATCACGGACTCCTTGGC |  |
| Sco2149_TMD_ RRAD | GCCAAGGAGTCCGTGATCGGGGCCGATAAGCTGATCCGCAACACG | Sco2149_TMD_ Quikchange |
|  | CGTGTTGCGGATCAGCTTATCGGCCCCGATCACGGACTCCTTGGC |  |
| Sco2149_TMD_ ∆RR | TCCGTGATCGGGAAGCTGATCCGCAACACGATGCTG | Sco2149_TMD_ Quikchange |
|  | CGCCTAGTCGAAGGGCTAGTGCCTGAGGAACCGTGG |  |
| Sco2149_TMD_ ∆118-122 | GCGGGCGCGGTCCTGATGTCCGACGAGGAGGTCGCC | Sco2149_TMD_ Modified Quikchange |
|  | GTCGGACATCAGGACCGCGCCCGCGCCGATGGCGAA |  |
| Sco2149_TMD_ ∆123-127 | TGGGCCCGCACCGAGGTCGCCGACGAGCGTCACCCG | Sco2149_TMD_ Modified Quikchange |
|  | GTCGGCGACCTCGGTGCGGGCCCAGTGGACCGCGCC |  |
| Sco2149_TMD_ ∆128-132 | ATGTCCGACGAGCGTCACCCGATCGAGGCGTCCCCC | Sco2149_TMD_ Modified Quikchange |
|  | GATCGGGTGACGCTCGTCGGACATCAGGGTGCGGGC |  |
| Sco2149_TMD_ ∆133-137 | GTCGCCGACGAGGCGTCCCCCGAGGTCCGTGCCAAG | Sco2149_TMD_ Modified Quikchange |
|  | CTCGGGGGACGCGTCGGCGACCTCCTCGTCGGACAT |  |
| Sco2149_TMD_ ∆138-142 | CACCCGATCGAGCGTGCCAAGGTCCACGCGGACTTC | Sco2149_TMD_ Modified Quikchange |
|  | GACCTTGGCACGCTCGATCGGGTGACGCTCGTCGGC |  |
| Sco2149_TMD_ ∆143-147 | TCCCCCGAGGTCGCGGACTTCAAGCAGGGTGCCAAG | Sco2149_TMD_ Modified Quikchange |
|  | CTTGAAGTCCGCGACCTCGGGGGACGCCTCGATCGG |  |
| Sco2149_TMD_ ∆148-152 | GCCAAGGTCCACGGTGCCAAGGAGTCCGTGATCGGG | Sco2149_TMD_ Modified Quikchange |
|  | CTCCTTGGCACCGTGGACCTTGGCACGGACCTCGGG |  |
| Sco2149_TMD_ ∆153-157 | GACTTCAAGCAGGTGATCGGGCGCCGCAAGCTGATC | Sco2149_TMD_ Modified Quikchange |
|  | GCGCCCGATCACCTGCTTGAAGTCCGCGTGGACCTT |  |
| Sco2149_TMD_ ∆118-127 | GCGGGCGCGGTCGAGGTCGCCGACGAGCGTCACCCGATC | Sco2149_TMD_ Modified Quikchange |
|  | GTCGGCGACCTCGACCGCGCCCGCGCCGATGGCGAA |  |
| Sco2149_TMD_ ∆128-137 | ATGTCCGACGAGGCGTCCCCCGAGGTCCGTGCCAAG | Sco2149_TMD_ Modified Quikchange |
|  | CTCGGGGGACGCCTCGTCGGACATCAGGGTGCGGGC |  |
| Sco2149_TMD_ ∆138-147 | CACCCGATCGAGGCGGACTTCAAGCAGGGTGCCAAG | Sco2149_TMD_ Modified Quikchange |
|  | CTTGAAGTCCGCCTCGATCGGGTGACGCTCGTCGGC |  |
| Sco2149_TMD_ ∆148-157 | GCCAAGGTCCACGTGATCGGGCGCCGCAAGCTGATC | Sco2149_TMD_ Modified Quikchange |
|  | GCGCCCGATCACGTGGACCTTGGCACGGACCTCGGG |  |
| Sco2149_TMD_ ∆131-132 | GAGGAGGTCGCCCGTCACCCGATCGAGGCGTCCCCC | Sco2149_TMD_ Modified Quikchange |
|  | GATCGGGTGACGGGCGACCTCCTCGTCGGACATCAG |  |
| Sco2149_TMD_ ∆126-128 | ACCCTGATGTCCGTCGCCGACGAGCGTCACCCGATC | Sco2149_TMD_ Modified Quikchange |
|  | CTCGTCGGCGACGGACATCAGGGTGCGGGCCCAGTG |  |
| Sco2149_TMD_ ∆137 ∆141 | CACCCGATCGCGTCCCCCGTCCGTGCCAAGGTCCACGCG | Sco2149_TMD_ Modified Quikchange |
|  | GGCACGGACGGGGGACGCGATCGGGTGACGCTCGTCGGC |  |
| Sco2149_TMD_ ∆149 ∆156 | AAGGTCCACGCGTTCAAGCAGGGTGCCAAGTCCGTGATCGGG | Sco2149_TMD_ Modified Quikchange |
|  | CCCGATCACGGACTTGGCACCCTGCTTGAACGCGTGGACCTT |  |
| Sco2149_TMD_ E137A E141A | CGTCACCCGATCGCGGCGTCCCCCGCGGTCCGTGCCAAG | Sco2149_TMD_ Quikchange |
|  | CTTGGCACGGACCGCGGGGGACGCCGCGATCGGGTGACG |  |
| Sco2149_TMD_ D131A E132A | GACGAGGAGGTCGCCGCGGCGCGTCACCCGATCGAG | Sco2149_TMD_ Quikchange |
|  | CTCGATCGGGTGACGCGCCGCGGCGACCTCCTCGTC |  |
| Sco2149_TMD_ D126A E127A E128A | GCCCGCACCCTGATGTCCGCGGCGGCGGTCGCCGACGAGCGTCAC | Sco2149_TMD_ Quikchange |
|  | GTGACGCTCGTCGGCGACCGCCGCCGCGGACATCAGGGTGCGGGC |  |
| Sco2149_TMD_ Ins D129 E130 E131 | TCCGACGAGGAGGACGAGGAGGTCGCCGACGAGCGTCACCCGATC | Sco2149_TMD_ Modified Quikchange |
|  | CTCGTCGGCGACCTCCTCGTCCTCCTCGTCGGACATCAGGGTGCG |  |
| Sco2149_TMD_ ∆126-8 ∆131-2 | ATGTCCGTCGCCCGTCACCCGATCGAGGCGTCCCCC | Sco2149_TMD_ Modified Quikchange |
|  | GATCGGGTGACGGGCGACGGACATCAGGGTGCGGGC |  |
| Sco2149_TMD_ ∆126-7 | ACCCTGATGTCCGAGGTCGCCGACGAGCGTCACCCG | Sco2149_TMD_ Modified Quikchange |
|  | GTCGGCGACCTCGGACATCAGGGTGCGGGCCCAGTG |  |
| Sco2149_TMD_ ∆127-8 | CTGATGTCCGACGTCGCCGACGAGCGTCACCCGATC | Sco2149_TMD_ Modified Quikchange |
|  | CTCGTCGGCGACGTCGGACATCAGGGTGCGGGCCCA |  |
| Sco2149_TMD_ ∆118-132 | GCGGGCGCGGTCCGTCACCCGATCGAGGCGTCCCCC | Sco2149_TMD_ Modified Quikchange |
|  | GATCGGGTGACGGACCGCGCCCGCGCCGATGGCGAA |  |
| Sco2149_TMD_ ∆123-137 | TGGGCCCGCACCGCGTCCCCCGAGGTCCGTGCCAAG | Sco2149_TMD_ Modified Quikchange |
|  | CTCGGGGGACGCGGTGCGGGCCCAGTGGACCGCGCC |  |
| Sco2149_TMD_ ∆128-142 | ATGTCCGACGAGCGTGCCAAGGTCCACGCGGACTTC | Sco2149_TMD_ Modified Quikchange |
|  | GACCTTGGCACGCTCGTCGGACATCAGGGTGCGGGC |  |
| Sco2149_TMD_ ∆133-147 | GTCGCCGACGAGGCGGACTTCAAGCAGGGTGCCAAG | Sco2149_TMD_ Modified Quikchange |
|  | CTTGAAGTCCGCCTCGTCGGCGACCTCCTCGTCGGA |  |
| Sco2149_TMD_ ∆138-152 | CACCCGATCGAGGGTGCCAAGGAGTCCGTGATCGGG | Sco2149_TMD_ Modified Quikchange |
|  | CTCCTTGGCACCCTCGATCGGGTGACGCTCGTCGGC |  |
| Sco2149_TMD_ ∆143-157 | TCCCCCGAGGTCGTGATCGGGCGCCGCAAGCTGATC | Sco2149_TMD_ Modified Quikchange |
|  | GCGCCCGATCACGACCTCGGGGGACGCCTCGATCGG |  |
| Sco2149_TMD_ ∆118-137 | GCGGGCGCGGTCGCGTCCCCCGAGGTCCGTGCCAAG | Sco2149_TMD_ Modified Quikchange |
|  | CTCGGGGGACGCGACCGCGCCCGCGCCGATGGCGAA |  |
| Sco2149_TMD_ ∆138-157 | CACCCGATCGAGGTGATCGGGCGCCGCAAGCTGATC | Sco2149_TMD_ Modified Quikchange |
|  | GCGCCCGATCACCTCGATCGGGTGACGCTCGTCGGC |  |
| Sco2149_TMD_ ∆131-2 | GCGCATGTCCGTCGCCCGTCACCCGATCGCGTCCCCCGTC | Sco2149_TMD_ Modified Quikchange |
|  | GCGCGATCGGGTGACGGGCGACGGACATCAGGGTGCGGGC |  |
| Sco2149_TMD_ D126K E127K E128K | GCGCACCCTGATGTCCAAGAAGAAGGTCGCCGACGAGCGTCACCCG | Sco2149_TMD_ Quikchange |
|  | GCGCCTCGTCGGCGACCTTCTTCTTGGACATCAGGGTGCGGGCCCA |  |
| Sco2149_TMD_ D131K E132K | GAGGAGGTCGCCAAGAAGCGTCACCCGATCGAGGCGTCC | Sco2149_TMD_ Quikchange |
|  | GATCGGGTGACGCTTCTTGGCGACCTCCTCGTCGGACAT |  |
| Sco2149_TMD_ E137K E141K | CGTCACCCGATCAAGGCGTCCCCCAAGGTCCGTGCCAAGGTCCACGCG | Sco2149_TMD_ Quikchange |
|  | CTTGGCACGGACCTTGGGGGACGCCTTGATCGGGTGACGCTCGTCGGC |  |
| Sco2149_TMD_ ∆118-142 | GCGGGCGCGGTCCGTGCCAAGGTCCACGCGGACTTC | Sco2149_TMD_ Modified Quikchange |
|  | GACCTTGGCACGGACCGCGCCCGCGCCGATGGCGAA |  |
| Sco2149_TMD_ ∆123-147 | TGGGCCCGCACCGCGGACTTCAAGCAGGGTGCCAAG | Sco2149_TMD_ Modified Quikchange |
|  | CTTGAAGTCCGCGGTGCGGGCCCAGTGGACCGCGCC |  |
| Sco2149_TMD_ ∆128-152 | ATGTCCGACGAGGGTGCCAAGGAGTCCGTGATCGGG | Sco2149_TMD_ Modified Quikchange |
|  | CTCCTTGGCACCCTCGTCGGACATCAGGGTGCGGGC |  |
| Sco2149_TMD_ ∆133-157 | GTCGCCGACGAGGTGATCGGGCGCCGCAAGCTGATC | Sco2149_TMD_ Modified Quikchange |
|  | GCGCCCGATCACCTCGTCGGCGACCTCCTCGTCGGA |  |
| Sco2149_TMD_ ∆118-147 | GCGGGCGCGGTCGCGGACTTCAAGCAGGGTGCCAAG | Sco2149_TMD_ Modified Quikchange |
|  | CTTGAAGTCCGCGACCGCGCCCGCGCCGATGGCGAA |  |
| Sco2149_TMD_ ∆123-152 | TGGGCCCGCACCGGTGCCAAGGAGTCCGTGATCGGG | Sco2149_TMD_ Modified Quikchange |
|  | CTCCTTGGCACCGGTGCGGGCCCAGTGGACCGCGCC |  |
| Sco2149_TMD_ ∆128-157 | ATGTCCGACGAGGTGATCGGGCGCCGCAAGCTGATC | Sco2149_TMD_ Modified Quikchange |
|  | GCGCCCGATCACCTCGTCGGACATCAGGGTGCGGGC |  |
| Sco2149_TMD_ D131KE132K | AAGAAGGTCGCCAAGAAGCGTCACCCGATCAAGGCGTCC | Sco2149_TMD_ Quikchange |
|  | GATCGGGTGACGCTTCTTGGCGACCTTCTTCTTGGACAT |  |
| Sco2149_TMD_ ∆118-152 | GCGGGCGCGGTCGGTGCCAAGGAGTCCGTGATCGGG | Sco2149_TMD_ Modified Quikchange |
|  | CTCCTTGGCACCGACCGCGCCCGCGCCGATGGCGAA |  |
| Sco2149_TMD_ ∆123-157 | TGGGCCCGCACCGTGATCGGGCGCCGCAAGCTGATC | Sco2149_TMD_ Modified Quikchange |
|  | GCGCCCGATCACGGTGCGGGCCCAGTGGACCGCGCC |  |
| Sco2149_TMD_ ∆118-157 | GCGGGCGCGGTCGTGATCGGGCGCCGCAAGCTGATC | Sco2149_TMD_ Modified Quikchange |
|  | GCGCCCGATCACGACCGCGCCCGCGCCGATGGCGAA |  |
| Sco2149_TMD_ ∆118-153 | GCGGGCGCGGTCGCCAAGGAGTCCGTGATCGGGCGC | Sco2149_TMD_ Modified Quikchange |
|  | GGACTCCTTGGCGACCGCGCCCGCGCCGATGGCGAA |  |
| Sco2149_TMD_ ∆118-154 | GCGGGCGCGGTCAAGGAGTCCGTGATCGGGCGCCGC | Sco2149_TMD_ Modified Quikchange |
|  | CACGGACTCCTTGACCGCGCCCGCGCCGATGGCGAA |  |
| Sco2149_TMD_ ∆118-155 | GCGGGCGCGGTCGAGTCCGTGATCGGGCGCCGCAAG | Sco2149_TMD_ Modified Quikchange |
|  | GATCACGGACTCGACCGCGCCCGCGCCGATGGCGAA |  |
| Sco2149_TMD_ ∆118-156 | GCGGGCGCGGTCTCCGTGATCGGGCGCCGCAAGCTG | Sco2149_TMD_ Modified Quikchange |
|  | CCCGATCACGGAGACCGCGCCCGCGCCGATGGCGAA |  |
| Sco2149_TMD_ Δ124-157 | GCCCGCACCCTGGTGATCGGGCGCCGCAAGCTGATC | Sco2149_TMD_ Modified Quikchange |
|  | GCGCCCGATCACCAGGGTGCGGGCCCAGTGGACCGC |  |
| Sco2149_TMD_ Δ125-157 | CGCACCCTGATGGTGATCGGGCGCCGCAAGCTGATC | Sco2149_TMD_ Modified Quikchange |
|  | GCGCCCGATCACCATCAGGGTGCGGGCCCAGTGGAC |  |
| Sco2149_TMD_ V158K | GGTGCCAAGGAGTCCAAGATCGGGCGCCGCAAG | Sco2149_TMD_ Quikchange |
|  | CTTGCGGCGCCCGATCTTGGACTCCTTGGCACC |  |
|  | CTTGCGGCGCCCGATCTTGGACTCCTTGACCGC |  |
| Sco2149_TMD_ ∆118-155 V158K | GGCGCGGTCGAGTCCAAGATCGGGCGCCGCAAG | Sco2149_TMD_ Quikchange |
|  | CTTGCGGCGCCCGATCTTGGACTCGACCGCGCC |  |
| Sco2149_TMD_ ∆118-156 V158K | GCGGGCGCGGTCTCCAAGATCGGGCGCCGCAAG | Sco2149_TMD_ Quikchange |
|  | CTTGCGGCGCCCGATCTTGGAGACCGCGCCCGC |  |
| Sco2149_TMD_ ∆118-157 V158K | GGCGCGGGCGCGGTCAAGATCGGGCGCCGCAAG | Sco2149_TMD_ Quikchange |
|  | CTTGCGGCGCCCGATCTTGACCGCGCCCGCGCC |  |
| Sco2149_TMD_ S179L | ACCCTGGTGCCGCTCCTGGGCGTCGTCCTGCTGCGC | Sco2149_TMD_ Quikchange |
|  | GCGCAGCAGGACGACGCCCAGGAGCGGCACCAGGGT |  |
| Sco2149_TMD_ G180L | ACCCTGGTGCCGCTCTCCCTGGTCGTCCTGCTGCGC | Sco2149_TMD_ Quikchange |
|  | GCGCAGCAGGACGACCAGGGAGAGCGGCACCAGGGT |  |
| Sco2149_TMD_ S179LG180L | ACCCTGGTGCCGCTCCTGCTGGTCGTCCTGCTGCGC | Sco2149_TMD_ Quikchange |
|  | GCGCAGCAGGACGACCAGCAGGAGCGGCACCAGGGT |  |
| Sco2149_TMD_ P177L S179L G180L | GCGCTCACCCTGGTGCTGCTCCTGCTGGTCGTCCTGCTGCGC | Sco2149_TMD_ Quikchange |
|  | GCGCAGCAGGACGACCAGCAGGAGCAGCACCAGGGTGAGCGC |  |
| Sco2149_TMD_ R185A | TCCGGCGTCGTCCTGCTGGCGTCTAGACACCCAGAAACG | Sco2149_TMD_ Quikchange |
|  | CGTTTCTGGGTGTCTAGACGCCAGCAGGACGACGCCGGA |  |
| Sco3746For | GCGCAGATCTATGGGTCACTGGCCCTCTGGGAGTGGGGCCCCG | Sco3746_TMD_ amplification |
| Sco3746Rev | GCGCTCTAGAGCTGCGCCCCGCGTTCGTGGCGCCAATCAGC |  |
| Sco3746(252)rev | GCGCTCTAGACAGCGGACTGTCGAAGCTGCGCCCCGCGTT | Construction of Sco3746_TMD_Bla |
| Sco3746_TMD_Bla For | GCGCACGAACGCGGGGCGCAGCGGTACCCACCCAGAAACGCTGGTG | Quickchange of *Xba*I site out of Sco3746_TMD_Bla (replaced with *Kpn*I) |
| Sco3746_TMD_Bla Rev | CACCAGCGTTTCTGGGTGGGTACCGCTGCGCCCCGCGTTCGTGCGC |  |
| Sco3746_TMD_extension | CGCGCCATGGTGGCAAGCCTGGCGGCGGGCCAAGCCCGGGCGGCACGCCCCTCTCGTGCCGCCACCGGGCGTCGCCTGACAGCTTCGACGCGGG | Construction of Sco3746_TMD_ extended |
| Sco3746_TMD_ RRKK | CCGGCCGCGCCCACCGTCTCGAAGAAGGGGGCCCTGTGGTTCGTCGGG | Sco3746_TMD_ Quikchange |
|  | CCCGACGAACCACAGGGCCCCCTTCTTCGAGACGGTGGGCGCGGCCGG |  |
| Sco3746_TMD_ G14C | GCGCGAGTGGGGCCCCGGCCTACTGTCGGTCCATGCGCGACC | Sco3746_TMD_ Quikchange |
|  | GGTCGCGCATGGACCGACAGTAGGCCGGGGCCCCACTCGCGC |  |
| Sco3746_TMD_ G84C | GCGCcccggacaaggggatcctctgcttctacctcttcgcctGG | Sco3746_TMD_ Quikchange |
|  | CCAGGCGAAGAGGTAGAAGCAGAGGATCCCCTTGTCCGGGGCGC |  |
| Sco3746_TMD_ A137C | GCGCccgccggtccgctcgctctgccacgcgctggagcggatct | Sco3746_TMD_ Quikchange |
|  | AGATCCGCTCCAGCGCGTGGCAGAGCGAGCGGACCGGCGGGCGC |  |
| Sco3746_TMD_ G171C | GCGCagctggactacctgtttccctgttccttctacccgctgca | Sco3746_TMD_ Quikchange |
|  | TGCAGCGGGTAGAAGGAACAGGGAAACAGGTAGTCCAGCTGCGC |  |
| Sco3746_TMD_ A219C | GCGCcgacctggtctccccgcgcccgtgcgcgcccaccgtctcgcgg | Sco3746_TMD_ Quikchange |
|  | CCGCGAGACGGTGGGCGCGCACGGGCGCGGGGAGACCAGGTCGGCGC |  |
| Sco3746_TMD_ G234L S235L | TTCGTCGGGGGCCTGCTGCTGCTGATGTTC | Sco3746_TMD_ Quikchange |
|  | GAACATCAGCAGCAGCAGGCCCCCGACGAA |  |
| Sco3746_TMD_ G234L S235L M239L F240L | TTCGTCGGGGGCCTGCTGCTGCTGCTGCTGGCCACGAACGCG | Sco3746_TMD_ Quikchange |
|  | CGCGTTCGTGGCCAGCAGCAGCAGCAGCAGGCCCCCGACGAA |  |
| PFD_TMD_BlaRev | GCGCGGTACCAAGCAGTGGGTTAGCTCTACCTTCCGGTGGGTTGGATACGCG | Construction of PFD_TMD_Bla |
| PFD_TMD_ extension | GCGCGGTACCCGGACGAATAAGCAGTGGGTTAGCTCTACCTTCCGGTGGGTTGGATACGCG | Construction of PFD_TMD_ extended |
| PFD_TMD_ RRKK | GTGGCCGGTCCGGATCTGGGCAAGAAGCGCCTCCTGGGTGCCGCGGCA | PFD_TMD_ Quikchange |
|  | TGCCGCGGCACCCAGGAGGCGCTTCTTGCCCAGATCCGGACCGGCCAC |  |
| PFD_TMD_ G354L R358L | GGCCTGGTAGTCCTGCCGCTCCTGCTGGTATCCAACCCA | PFD_TMD_ Quikchange |
|  | TGGGTTGGATACCAGCAGGAGCGGCAGGACTACCAGGCC |  |
| PFD_TMD_ G354L P355L R358L | GGCCTGGTAGTCCTGCTGCTCCTGCTGGTATCCAACCCA | PFD_TMD_ Quikchange |
|  | TGGGTTGGATACCAGCAGGAGCAGCAGGACTACCAGGCC |  |

For all Quikchange primers, the forward primer sequence is given first and the reverse second. Restriction enzymes sites are underlined
